# Supplementary material for: Unraveling the Interplay of KRAS, NRAS, BRAF, and Micro-Satellite Instability in Non-Metastatic Colon Cancer: A Systematic Review
Source: Diagnostics (Basel). 2024 May 12;14(10):1001. doi: 10.3390/diagnostics14101001 (PMC11120454; doi:10.3390/diagnostics14101001)
Supplement: Supplementary file 1 [file diagnostics-14-01001-s001.zip › diagnostics-2951976-supplementary.pdf]

Table S1. article excluded with reason and round.

key	Title	reason for exclusion	round of exclusion
rayyan-661154035	Chapter 1 - Special Diagnostic Techniques in Surgical Pathology	wrong population	round I
rayyan-661154036	The natural history of adenomas	wrong population	round I
rayyan-661154037	20 - Molecular pathology	wrong population	round I
rayyan-661154038	Chapter 6 - Gastrointestinal System	wrong population	round I
rayyan-661154039	Chapter 164 - Adenocarcinoma of the Colon and Rectum	wrong population	round I
rayyan-661154040	Chapter Four - Implementing circulating tumor DNA analysis in a clinical laboratory: A user manual	wrong population	round I
rayyan-661154041	13 - Tumours and Tumour-like Lesions of the Liver	wrong population	round I
rayyan-661154042	The therapeutic landscape of hepatocellular carcinoma	wrong population	round I
rayyan-661154043	Chapter 12 - Genetics of Adrenal Tumors	wrong population	round I
rayyan-661154044	86 - Carcinoma of the Ovaries and Fallopian Tubes	wrong population	round I
rayyan-661154046	13 - Neoplasms of the Testis	wrong population	round I
rayyan-661154047	Chapter 4 - Tumor Heterogeneity	wrong population	round I
rayyan-661154048	Glossary	wrong population	round I
rayyan-661154049	5 - Neoplasia	wrong population	round I
rayyan-661154050	The role of cell cycle regulatory proteins in the pathogenesis of melanoma	wrong population	round I
rayyan-661154051	Chapter 3 - Pharmacogenetics of therapeutics	wrong population	round I
rayyan-661154052	"Chapter 6 - Pharmacoepigenetic Processors: Epigenetic Drugs	wrong population	round I
rayyan-661154053	Intraductal Papillary Mucinous Neoplasm: A Clinicopathologic Review	wrong population	round I
rayyan-661154054	Chapter Seven - Synergistic combination of oncolytic virotherapy with CAR T-cell therapy	wrong population	round I
rayyan-661154055	Chapter 57 - Pharmacology and Molecular Mechanisms of Antineoplastic Agents for Hematologic Malignancies	wrong population	round I
rayyan-661154056	Gastroenteropancreatic endocrine tumors	wrong population	round I
rayyan-661154057	Chapter 9 - Hereditary predisposition to uveal melanoma	wrong population	round I
rayyan-661154058	Chapter 4 - Precision Surgery and Surgical Spectroscopy	wrong population	round I
rayyan-661154059	Architects meets Repairers: The interplay between homeobox genes and DNA repair	wrong population	round I

rayyan-661154060	1 - Ovarian Carcinoma: An Introduction	wrong population	round I
rayyan-661154061	7 - Special Studies	wrong population	round I
rayyan-661154062	CHAPTER 7 - Molecular Biology	wrong population	round I
rayyan-661154063	47 - Cholangiocarcinoma	wrong population	round I
rayyan-661154064	Kapitel 5 - Viszeralchirurgie	wrong population	round I
rayyan-661154065	Index	wrong population	round I
rayyan-661154066	Chapter 12 - Therapies II: Specific Non-surgical Treatments	wrong population	round I
rayyan-661154067	Chapter 11 - Clinical Trial Design in Immuno-Oncology	wrong population	round I
rayyan-661154068	Subject Index	wrong population	round I
rayyan-661154069	Chapter 11 - Surface Epithelial Stromal Tumors of the Ovary	wrong population	round I
rayyan-661154070	Molecular techniques in anatomic pathology: An overview	wrong population	round I
rayyan-661154071	Molecular biology of testicular germ cell tumors: Unique features awaiting clinical application	wrong population	round I
rayyan-661154072	Cholangiocarcinoma: New Insights into Disease Pathogenesis and Biology	wrong population	round I
rayyan-661154073	6 - Salivary Glands	wrong population	round I
rayyan-661154074	Some thoughts on what's ahead for GI in 2006 and beyond	wrong population	round I
rayyan-661154075	Explaining the unexplainable: discrepancies in results from the CALGB/SWOG 80405 and FIRE-3 studies	wrong population	round I
rayyan-661154076	CHAPTER 42 - Tumour markers	wrong population	round I
rayyan-661154077	1 - Pancreatic Carcinoma: An Introduction	wrong population	round I
rayyan-661154078	Chapter Three - Advances in Circulating Tumor DNA Analysis	wrong population	round I
rayyan-661154079	The Gastrointestinal Tumor Microenvironment	wrong population	round I
rayyan-661154080	"Chapter 14 - Biomarkers: Discovery	wrong population	round I
rayyan-661154081	Index	wrong population	round I
rayyan-661154082	CHAPTER 23 - Large Intestine (Colon)	wrong population	round I
rayyan-661154083	Mechanisms regulating T-cell infiltration and activity in solid tumors	wrong population	round I
rayyan-661154084	Chapter 99 - Vitamin D and Colon Cancer	wrong population	round I
rayyan-661154086	Index	wrong population	round I

rayyan-661154087	"Chapter 10 - Typing	wrong population	round I
rayyan-661154088	Chapter 13 - Tumours and Tumour-Like Lesions	wrong population	round I
rayyan-661154091	Chapter 7 - Hepatobiliary System	wrong population	round I
rayyan-661154095	Molecular diagnostics in melanoma	wrong population	round I
rayyan-661154098	Subject Index	wrong population	round I
rayyan-661154108	13 - Genetic Factors: Hereditary Cancer Predisposition Syndromes	wrong population	round I
rayyan-661154109	Immune cellular components and signaling pathways in the tumor microenvironment	wrong population	round I
rayyan-661154111	Genetic Changes in Ovarian Cancer	wrong population	round I
rayyan-661154114	Targeting the tumour microenvironment in platinum-resistant ovarian cancer	wrong population	round I
rayyan-661154115	Ovarian cancer: Novel molecular aspects for clinical assessment	wrong population	round I
rayyan-661154116	25 - Nanoparticle and Targeted Systems for Colon Cancer Therapy	wrong population	round I
rayyan-661154117	Predictive markers in early research and companion diagnostic developments in oncology	wrong population	round I
rayyan-661154118	Molecular Biology of Head and Neck Cancer: Risks and Pathways	wrong population	round I
rayyan-661154121	Chapter 5 - Mucinous ovarian carcinomas	wrong population	round I
rayyan-661154122	Chapter 8A - Pancreatic cancer and premalignant tumors: Molecular aspects	wrong population	round I
rayyan-661154123	Turning tumors from cold to inflamed to improve immunotherapy response	wrong population	round I
rayyan-661154125	Genetic profiling of hepatocellular carcinoma using next-generation sequencing	wrong population	round I
rayyan-661154126	"The Dualistic Model of Ovarian Carcinogenesis: Revisited	wrong population	round I
rayyan-661154129	Molecular Genetics of Pancreatic Ductal Adenocarcinomas and Recent Implications for Translational Efforts	wrong population	round I
rayyan-661154131	Personalized biomarker-based treatment strategy for patients with squamous cell carcinoma of the head and neck: EORTC position and approach	wrong population	round I
rayyan-661154132	Pathology of Endometrioid and Clear Cell Carcinoma of the Ovary	wrong population	round I
rayyan-661154133	Combination of Novel Agents with Radiotherapy to Treat Rectal Cancer	wrong population	round I
rayyan-661154134	Nanotechnology-based delivery of CRISPR/Cas9 for cancer treatment	wrong population	round I
rayyan-661154137	Genetics and Molecular Pathogenesis of Gastric Adenocarcinoma	wrong population	round I
rayyan-661154138	Sirtuins' control of autophagy and mitophagy in cancer	wrong population	round I
rayyan-661154140	HOX cluster-embedded micro-RNAs and cancer	wrong population	round I

rayyan-661154141	"Chapter 8 - Microbiota	wrong population	round I
rayyan-661154142	Pancreatic cancer	wrong population	round I
rayyan-661154143	Targeted therapies in germ cell tumors	wrong population	round I
rayyan-661154144	Morphological subtypes of ovarian carcinoma: a review with emphasis on new developments and pathogenesis	wrong population	round I
rayyan-661154145	Liquid Biopsy-Based Biomarkers of Treatment Response and Resistance	wrong population	round I
rayyan-661154146	Shaping functional gut microbiota using dietary bioactives to reduce colon cancer risk	wrong population	round I
rayyan-661154148	The War on Cancer Rages On	wrong population	round I
rayyan-661154149	89 - Cancers Arising in the Ovary	wrong population	round I
rayyan-661154150	13 - Genetic and Epigenetic Alterations in Cancer	wrong population	round I
rayyan-661154151	Chapter 5 - Intracranial metastases	wrong population	round I
rayyan-661154152	Prognostic Factors and Differences in Survival of Right and Left Colon Carcinoma: A STROBE Compliant Retrospective Cohort Study	wrong population	round I
rayyan-661154153	Odyssey of trefoil factors in cancer: Diagnostic and therapeutic implications	wrong population	round I
rayyan-661154156	Pancreatic Carcinogenesis	wrong population	round I
rayyan-661154157	Cytokine chemokine network in tumor microenvironment: Impact on CSC properties and therapeutic applications	wrong population	round I
rayyan-661154161	Molecular and serum markers in hepatocellular carcinoma: Predictive tools for prognosis and recurrence	wrong population	round I
rayyan-661154173	Microenvironment-driven intratumoral heterogeneity in head and neck cancers: clinical challenges and opportunities for precision medicine	wrong population	round I
rayyan-661154180	Cancer-associated genodermatoses: Skin neoplasms as clues to hereditary tumor syndromes	wrong population	round I
rayyan-661154183	Ovarian cancer	wrong population	round I
rayyan-661154184	Genetics and epigenetics of adrenocortical tumors	wrong population	round I
rayyan-661154189	Identification of potential therapeutic targets by molecular profiling of 628 cases of uterine serous carcinoma	wrong population	round I
rayyan-661154191	"Prognostic stratification of resected pancreatic ductal adenocarcinoma: Past	wrong population	round I
rayyan-661154203	"Biologics	wrong population	round I
rayyan-661154204	Integrated Genomic Characterization of Pancreatic Ductal Adenocarcinoma	wrong population	round I
rayyan-661154209	DNA damage response and repair in ovarian cancer: Potential targets for therapeutic strategies	wrong population	round I
rayyan-661154210	Targeting TRK: A fast-tracked application of precision oncology and future directions	wrong population	round I
rayyan-661154211	Immune checkpoint blockade and its combination therapy with small-molecule inhibitors for cancer treatment	wrong population	round I

rayyan-661154212	Aspirin in pancreatic cancer: chemopreventive effects and therapeutic potentials	wrong population	round I
rayyan-661154214	The Changing Face of Percutaneous Image-guided Biopsy: Molecular Profiling and Genomic Analysis in Current Practice	wrong population	round I
rayyan-661154215	Pathology of borderline and invasive cancers	wrong population	round I
rayyan-661154216	Challenges and opportunities of cfDNA analysis implementation in clinical practice: Perspective of the International Society of Liquid Biopsy (ISLB)	wrong population	round I
rayyan-661154217	Stromal PD-1/PD-L1 Expression Predicts Outcome in Colon Cancer Patients	wrong population	round I
rayyan-661154218	Reconciling environment-mediated metabolic heterogeneity with the oncogene-driven cancer paradigm in precision oncology	wrong population	round I
rayyan-661154221	"The biological complexity of urothelial carcinoma: Insights into carcinogenesis	wrong population	round I
rayyan-661154222	Chapter Two - The Role of Neoantigens in Naturally Occurring and Therapeutically Induced Immune Responses to Cancer	wrong population	round I
rayyan-661154225	Dysregulation of ubiquitin ligases in cancer	wrong population	round I
rayyan-661154226	Can integrative biomarker approaches improve prediction of platinum and PARP inhibitor response in ovarian cancer?	wrong population	round I
rayyan-661154232	Liquid biopsy utility for the surveillance of cutaneous malignant melanoma patients	wrong population	round I
rayyan-661154233	Targeted and immunotherapy in the era of personalised gastric cancer treatment	wrong population	round I
rayyan-661154235	Hereditary Ovarian Cancer and Risk Reduction	wrong population	round I
rayyan-661154237	Clinicopathological and prognostic significance of PD-L1 expression in colon adenocarcinoma tumor budding	wrong population	round I
rayyan-661154238	Targeting the DNA damage response: PARP inhibitors and new perspectives in the landscape of cancer treatment	wrong population	round I
rayyan-661154241	Therapy of Primary Liver Cancer	wrong population	round I
rayyan-661154242	Inflammatory bowel disease drastically affects the prognosis of patients treated for peritoneal metastases with combined cytoreductive surgery and hyperthermic intraperitoneal chemotherapy: A multicenter study	wrong population	round I
rayyan-661154248	Endometriosis-associated ovarian cancer: What have we learned so far?	wrong population	round I
rayyan-661154249	microRNAs as pharmacological targets in cancer	wrong population	round I
rayyan-661154251	Prognostic and Predictive Markers in Stage II Colon Cancer: Is There a Role for Gene Expression Profiling?	wrong population	round I
rayyan-661154255	Precision medicine in gastroenteropancreatic neuroendocrine neoplasms: Where are we in 2023?	wrong population	round I
rayyan-661154257	Spatiotemporal heterogeneity and clinical challenge of pancreatic neuroendocrine tumors	wrong population	round I
rayyan-661154261	Biomarkers for predicting the outcome of various cancer immunotherapies	wrong population	round I
rayyan-661154262	Current Controversies in the Management of Colon Cancer	wrong population	round I
rayyan-661154263	"Pathogenesis	wrong population	round I

rayyan-661154266	"27 - Molecular oncology in gynecologic cancer: Immunologic Response	wrong population	round I
rayyan-661154268	6.05 - Ovarian Cancer: Towards Personalizing Ovarian Cancer Treatments Using Patient-Derived Organoids	wrong population	round I
rayyan-661154271	Circulating Methylated Septin 9 Nucleic Acid in the Plasma of Patients with Gastrointestinal Cancer in the Stomach and Colon	wrong population	round I
rayyan-661154273	Retinal toxicities of systemic anticancer drugs	wrong population	round I
rayyan-661154274	Mitogen Inducible Gene-6 Is a Prognostic Marker for Patients with Colorectal Liver Metastases	wrong population	round I
rayyan-661154278	Next generation sequencing for liquid biopsy based testing in non-small cell lung cancer in 2021	wrong population	round I
rayyan-661154280	Molecular diagnostics and biomarkers in cholangiocarcinoma	wrong population	round I
rayyan-661154284	Updates and emerging therapies for rare epithelial ovarian cancers: One size no longer fits all	wrong population	round I
rayyan-661154289	Chapter 35 - Molecular Assessment of Ovarian Cancer and Translation to Clinical Management	wrong population	round I
rayyan-661154292	Ovarian Cancer: Pathology and Genetics	wrong population	round I
rayyan-661154296	Understanding and overcoming the resistance of cancer to PD-1/PD-L1 blockade	wrong population	round I
rayyan-661154300	Targeting specific molecular pathways holds promise for advanced gallbladder cancer therapy	wrong population	round I
rayyan-661154302	Clinical treatment of cholangiocarcinoma: an updated comprehensive review	wrong population	round I
rayyan-661154304	Mucinous Colloid Carcinoma of the Colon Metastatic to the Breast	wrong population	round I
rayyan-661154307	Chapter 14 - Cancer Vaccines	wrong population	round I
rayyan-661154310	Endometrial cancer: Molecular markers and management of advanced stage disease	wrong population	round I
rayyan-661154313	Mechanism and potential predictive biomarkers of immune checkpoint inhibitors in NSCLC	wrong population	round I
rayyan-661154315	Update on current problems in colorectal liver metastasis	wrong population	round I
rayyan-661154317	Deciphering the complexities of cancer cell immune evasion: Mechanisms and therapeutic implications	wrong population	round I
rayyan-661154319	Progress and challenges in gastroesophageal cancer	wrong population	round I
rayyan-661154321	Recent updates on innovative approaches to overcome drug resistance for better outcomes in cancer	wrong population	round I
rayyan-661154323	Genomic insights in gynecologic cancer	wrong population	round I
rayyan-661154324	Decoding the Molecular and Mutational Ambiguities of Gastroenteropancreatic Neuroendocrine Neoplasm Pathobiology	wrong population	round I
rayyan-661154325	Translational genomics of sinonasal cancers	wrong population	round I
rayyan-661154326	TRK fusion positive cancers: From first clinical data of a TRK inhibitor to future directions	wrong population	round I
rayyan-661154327	TGF-Î² Receptor Inactivation and Mutant Kras Induce Intestinal Neoplasms in Mice via a Î²-Catenin-Independent Pathway	wrong population	round I

rayyan-661154328	Looking beyond drivers and passengers in cancer genome sequencing data	wrong population	round I
rayyan-661154331	Emerging avenues linking inflammation and cancer	wrong population	round I
rayyan-661154334	Biomarker discovery and applicationâ€"An opportunity to resolve the challenge of liver cancer diagnosis and treatment	wrong population	round I
rayyan-661154335	Multicohort Retrospective Validation of a Predictive Biomarker for Topoisomerase I Inhibitors	wrong population	round I
rayyan-661154340	Identifying patients with NTRK fusion cancer	wrong population	round I
rayyan-661154346	Emerging biomarkers for cancer immunotherapy in melanoma	wrong population	round I
rayyan-661154348	Application of Immunohistochemistry and Molecular Diagnostics to Clinically Relevant Problems in Endometrial Cancer	wrong population	round I
rayyan-661154350	Endoscopic polypectomy for malignant polyps: Should tumor location (right versus left side) guide clinical decisions?	wrong population	round I
rayyan-661154352	Brain malignancies: Glioblastoma and brain metastases	wrong population	round I
rayyan-661154356	Immune profiling of pancreatic cancer for radiotherapy with immunotherapy and targeted therapy: Biomarker analysis of a randomized phase 2 trial	wrong population	round I
rayyan-661154357	Genetic mutations in gynaecological cancers	wrong population	round I
rayyan-661154358	Oncogenic Signaling Pathways in The Cancer Genome Atlas	wrong population	round I
rayyan-661154359	Modeling Human Digestive Diseases With CRISPR-Cas9â€"Modified Organoids	wrong population	round I
rayyan-661154362	Epigenetics and therapeutic targets in gastrointestinal malignancies	wrong population	round I
rayyan-661154366	Chapter 10 - Overcoming chemotherapy resistance in endometrial cancer	wrong population	round I
rayyan-661154367	Quantitative proteomic analysis of paired colorectal cancer and non-tumorigenic tissues reveals signature proteins and perturbed pathways involved in CRC progression and metastasis	wrong population	round I
rayyan-661154368	A chemoresistance lncRNA signature for recurrence risk stratification of colon cancer patients with chemotherapy	wrong population	round I
rayyan-661154371	Genetics of Pancreatic Cancer and Its Implications on Therapy	wrong population	round I
rayyan-661154372	Novel biomarkers in bladder cancer	wrong population	round I
rayyan-661154373	Distinct genetic changes characterise multifocality and diverse histological subtypes in papillary thyroid carcinoma	wrong population	round I
rayyan-661154374	Chapter 8 - Linking oxidative stress and ovarian cancers	wrong population	round I
rayyan-661154381	Insight updating of the molecular hallmarks in ovarian carcinoma	wrong population	round I
rayyan-661154382	"Identification of CRKL as an oncogenic biomarker for prognosis and immunotherapy in melanoma	wrong population	round I
rayyan-661154383	"Management of Stage IV rectal disease â€" How to incorporate radiation therapy	wrong population	round I
rayyan-661154385	Dealing with NSCLC EGFR mutation testing and treatment: A comprehensive review with an Italian real-world perspective	wrong population	round I

rayyan-661154390	Dysregulated FOXM1 signaling in the regulation of cancer stem cells	wrong population	round I
rayyan-661154391	Evolving role of regorafenib for the treatment of advanced cancers	wrong population	round I
rayyan-661154392	Integrative Clinical Genomics of Advanced Prostate Cancer	wrong population	round I
rayyan-661154393	Cell-free DNA in the blood as a solid tumor biomarkerâ€"A critical appraisal of the literature	wrong population	round I
rayyan-661154395	Chapter 22 - Neurological complications of GI cancers	wrong population	round I
rayyan-661154397	"Obesity	wrong population	round I
rayyan-661154401	Immuno-oncology in GI tumours: Clinical evidence and emerging trials of PD-1/PD-L1 antagonists	wrong population	round I
rayyan-661154402	When should we order a next generation sequencing test in a patient with cancer?	wrong population	round I
rayyan-661154404	Cancer of Unknown Primary in the Molecular Era	wrong population	round I
rayyan-661154405	High expression of meningioma 1 is correlated with reduced survival rates in colorectal cancer patients	wrong population	round I
rayyan-661154409	Advances in the systemic treatment of therapeutic approaches in biliary tract cancer	wrong population	round I
rayyan-661154410	A soluble LAG-3 protein (eftilagimod alpha) and an anti-PD-L1 antibody (avelumab) tested in a phase I trial: a new combination in immuno-oncology	wrong population	round I
rayyan-661154416	Proteome biology of primary colorectal carcinoma and corresponding liver metastases	wrong population	round I
rayyan-661154418	Diagnosis and treatment of cholangiocarcinoma in Italy: A Delphi consensus statement	wrong population	round I
rayyan-661154419	ETS transcription factors: Multifaceted players from cancer progression to tumor immunity	wrong population	round I
rayyan-661154420	"Molecular pathogenesis	wrong population	round I
rayyan-661154422	Proteomics in colorectal cancer translational research: Biomarker discovery for clinical applications	wrong population	round I
rayyan-661154425	Capturing cancer evolution using genetically engineered mouse models (GEMMs)	wrong population	round I
rayyan-661154430	Low frequency of somatic mutations in uterine sarcomas: A molecular analysis and review of the literature	wrong population	round I
rayyan-661154432	Health-Promoting Lifestyle in Colorectal Cancer Survivors: A Qualitative Study on the Experiences and Perspectives of Colorectal Cancer Survivors and Healthcare Providers	wrong population	round I
rayyan-661154438	Clinical Implications and Future Perspectives of Circulating Tumor Cells and Biomarkers in Clinical Outcomes of Colorectal Cancer	wrong population	round I
rayyan-661154448	Molecular mechanistic pathway of colorectal carcinogenesis associated with intestinal microbiota	wrong population	round I
rayyan-661154451	Molecular pathogenesis of gallbladder cancer: An update	wrong population	round I
rayyan-661154452	Intrathyroid thymic carcinoma: A clinicopathological analysis of 22 cases	wrong population	round I
rayyan-661154453	Not all cancers are created equal: Tissue specificity in cancer genes and pathways	wrong population	round I

rayyan-661154454	Use of molecular markers for predicting therapy response in cancer patients	wrong population	round I
rayyan-661154455	Genomic profiling in pancreatic ductal adenocarcinoma and a pathway towards therapy individualization: A scoping review	wrong population	round I
rayyan-661154456	A genome-wide assessment of variations of primary colorectal cancer maintained in metastases	wrong population	round I
rayyan-661154457	A guided tour of selected issues pertaining to metastatic carcinomas involving or originating from the gynecologic tract	wrong population	round I
rayyan-661154459	Current advances in prognostic and diagnostic biomarkers for solid cancers: Detection techniques and future challenges	wrong population	round I
rayyan-661154460	"Clinicopathological differences and survival outcomes with first-line therapy in patients with left-sided colon cancer and rectal cancer: Pooled analysis of 2879 patients from AGITG (MAX)	wrong population	round I
rayyan-661154463	Predicting liver metastases growth patterns: Current status and future possibilities	wrong population	round I
rayyan-661154465	Molecular targets and biological modifiers in gastric cancer	wrong population	round I
rayyan-661154466	Long noncoding RNA profiles identify five distinct molecular subtypes of colorectal cancer with clinical relevance	wrong population	round I
rayyan-661154467	"Clinical relevance of molecular diagnostics in gastrointestinal (GI) cancer: European Society of Digestive Oncology (ESDO) expert discussion and recommendations from the 17th European Society for Medical Oncology (ESMO)/World Congress on Gastrointestinal Cancer	wrong population	round I
rayyan-661154471	Altered RECQ Helicase Expression in Sporadic Primary Colorectal Cancers	wrong population	round I
rayyan-661154475	"Primary tumor type prediction based on US nationwide genomic profiling data in 13	wrong population	round I
rayyan-661154477	The impact of recent next generation sequencing and the need for a new classification in gastric cancer	wrong population	round I
rayyan-661154478	Ibuprofen Inhibits Colitis-Induced Overexpression of TumorRelated Rac1b	wrong population	round I
rayyan-661154480	DNA methylation in breast and colorectal cancers	wrong population	round I
rayyan-661154481	Identification of high-risk human papillomavirus and Rb/E2F pathway genomic alterations in mutually exclusive subsets of colorectal neuroendocrine carcinoma	wrong population	round I
rayyan-661154482	The application of circulating tumor cell and cell-free DNA liquid biopsies in ovarian cancer	wrong population	round I
rayyan-661154483	Survival outcomes for right-versus left-sided colon cancer and rectal cancer in England: A propensity-score matched population-based cohort study	wrong population	round I
rayyan-661154484	Chapter Two - Wnt/Î² Catenin-Mediated Signaling Commonly Altered in Colorectal Cancer	wrong population	round I
rayyan-661154486	"Development of Rationally Designed	wrong population	round I
rayyan-661154487	Oncogenic signaling pathways associated with immune evasion and resistance to immune checkpoint inhibitors in cancer	wrong population	round I
rayyan-661154488	"Methylation analysis of APC	wrong population	round I
rayyan-661154491	Early-onset gastric cancer is a distinct disease with worrisome trends and oncogenic features	wrong population	round I
rayyan-661154495	Molecular Diagnostics in the Neoplasms of Small Intestine and Appendix: 2018Â Update	wrong population	round I

rayyan-661154497	Influence of the primary tumour location in patients undergoing surgery for colorectal liver metastases	wrong population	round I
rayyan-661154499	Targeting c-MYC through Interference with NAMPT and SIRT1 and Their Association to Oncogenic Drivers in Murine Serrated Intestinal Tumorigenesis	wrong population	round I
rayyan-661154506	RAF Suppression Synergizes with MEK Inhibition in KRAS Mutant Cancer Cells	wrong population	round I
rayyan-661154509	Systemic Therapy for Patients With Pancreatic Cancer: Current Approaches and Opportunities for Novel Avenues Toward Precision Medicine	wrong population	round I
rayyan-661154510	Tissue and serum biomarkers as prognostic variables in endometrioid-type endometrial cancer	wrong population	round I
rayyan-661154514	The prognostic role of tumor associated glycoprotein 72 (TAG-72) in stage II and III colorectal adenocarcinoma	wrong population	round I
rayyan-661154518	Lynch syndrome-associated breast cancers do not overexpress chromosome 11-encoded mucins	wrong population	round I
rayyan-661154520	Phase Ib study of atezolizumab combined with cobimetinib in patients with solid tumors	wrong population	round I
rayyan-661154521	Endometrial carcinoma: pathology and genetics	wrong population	round I
rayyan-661154522	Pervasive conditional selection of driver mutations and modular epistasis networks in cancer	wrong population	round I
rayyan-661154527	Broadening the therapeutic horizon of advanced biliary tract cancer through molecular characterisation	wrong population	round I
rayyan-661154528	Does the site of primary colorectal cancer influence the outcome after resection of isolated liver metastases?	wrong population	round I
rayyan-661154531	The role of Fusobacterium nucleatum in colorectal cancer: from carcinogenesis to clinical management	wrong population	round I
rayyan-661154532	Decoding colorectal cancer epigenomics	wrong population	round I
rayyan-661154535	Discovery of Aberrant Alteration of Genome in Colorectal Cancer by Exome Sequencing	wrong population	round I
rayyan-661154536	Is More Not Better?: Combination Therapies in Colorectal Cancer Treatment	wrong population	round I
rayyan-661154544	Impact of molecular markers on treatment selection in advanced colorectal cancer	wrong population	round I
rayyan-661154545	ESMO recommendations on the use of circulating tumour DNA assays for patients with cancer: a report from the ESMO Precision Medicine Working Group	wrong population	round I
rayyan-661154547	Appendiceal goblet cell carcinoids and adenocarcinomas ex-goblet cell carcinoid are genetically distinct from primary colorectal-type adenocarcinoma of the appendix	wrong population	round I
rayyan-661154551	"Mouse models in colon cancer	wrong population	round I
rayyan-661154555	Molecular profiling of longitudinally observed small colorectal polyps: A cohort study	wrong population	round I
rayyan-661154557	Clinicopathologic and genetic characteristics of interval colorectal carcinomas favor origin from missed or incompletely excised precursors	wrong population	round I
rayyan-661154558	Hypoxia induced deregulation of sphingolipids in colon cancer is a prognostic marker for patient outcome	wrong population	round I
rayyan-661154559	MicroRNA-nanoparticles against cancer: Opportunities and challenges for personalized medicine	wrong population	round I
rayyan-661154562	Real-Time Targeted Genome Profile Analysis of Pancreatic Ductal Adenocarcinomas Identifies Genetic Alterations That Might Be Targeted With Existing Drugs or Used as Biomarkers	wrong population	round I

rayyan-661154567	NOTCH1 Mutations Occur Early during Cutaneous Squamous Cell Carcinogenesis	wrong population	round I
rayyan-661154568	"KRAS and aneusomy of chromosomes 4	wrong population	round I
rayyan-661154574	Identification of recurrent mutational events in anorectal melanoma	wrong population	round I
rayyan-661154577	Avelumab and cetuximab as a therapeutic combination: An overview of scientific rationale and current clinical trials in cancer	wrong population	round I
rayyan-661154584	Molecular Carcinogenesis of Endometrial Cancer	wrong population	round I
rayyan-661154586	First-Line Nivolumab Plus Low-Dose Ipilimumab for Microsatellite Instability-High/ Mismatch Repair-Deficient Metastatic Colorectal Cancer: the Phase II CheckMate 142 Study	wrong population	round I
rayyan-661154587	"Nivolumab in patients with metastatic DNA mismatch repair-deficient or microsatellite instability-high colorectal cancer (CheckMate 142): an open-label	wrong population	round I
rayyan-661154589	Cost-effectiveness analysis of pembrolizumab versus chemotherapy for microsatellite instability-high or mismatch repair-deficient metastatic colorectal cancer	wrong population	round I
rayyan-661154590	The role of genetic instability in stimulation of angiogenesis in liver metastases of sporadic colorectal cancer	wrong population	round I
rayyan-661154594	Pembrolizumab in Microsatellite-Instability-High Advanced Colorectal Cancer	wrong population	round I
rayyan-661154597	Meat consumption and BRAF mutation status in colorectal cancer	wrong population	round I
rayyan-661154598	Evaluation of Pembrolizumab (MK-3475) or Co-formulated Pembrolizumab/Quavonlimab (MK-1308A) in Participants With Microsatellite Instability-High (MSI-H) or Mismatch Repair Deficient (dMMR) Stage IV Colorectal Cancer (CRC) (MK-1308A-008)	wrong population	round I
rayyan-661154599	Pembrolizumab in Asian patients with microsatellite-instability-high/mismatch-repair-deficient colorectal cancer	wrong population	round I
rayyan-661154602	Pembrolizumab vs Chemotherapy in Microsatellite Instability-High or Mismatch Repair Deficient Stage IV Colorectal Cancer	wrong population	round I
rayyan-661154603	Radiomics-based prediction of microsatellite instability in colorectal cancer at initial computed tomography evaluation	wrong population	round I
rayyan-661154604	Facilitating informed decisions regarding microsatellite instability testing among high-risk individuals diagnosed with colorectal cancer	wrong population	round I
rayyan-661154605	Development and Interpretation of a Clinicopathological-Based Model for the Identification of Microsatellite Instability in Colorectal Cancer	wrong population	round I
rayyan-661154606	Instability of Non-Standard Microsatellites in Relation to Prognosis in Metastatic Colorectal Cancer Patients	wrong population	round I
rayyan-661154607	Educational CD-ROM Compared With Standard Informed Consent for Patients With Colorectal Cancer or a Family History of Colorectal Cancer	wrong population	round I
rayyan-661154608	Avelumab vs Standard Second-Line Chemotherapy in Patients With Metastatic Colorectal Cancer and Microsatellite Instability: a Randomized Clinical Trial	wrong population	round I
rayyan-661154610	Prognostic value of microsatellite instability and p53 expression in metastatic colorectal cancer treated with oxaliplatin and fluoropyrimidine-based chemotherapy	wrong population	round I
rayyan-661154612	"Tumor mutational load	wrong population	round I
rayyan-661154613	Genotype-environment interactions in microsatellite stable/microsatellite instability-low colorectal cancer: results from a genome-wide association study	wrong population	round I

rayyan-661154614	Evaluation of the Cost-effectiveness of Doublet Therapy in Metastatic BRAF Variant Colorectal Cancer	wrong population	round I
rayyan-661154615	Oncologists' selection of genetic and molecular testing in the evolving landscape of stage II colorectal cancer	wrong population	round I
rayyan-661154616	The Oncology Biomarker Discovery framework reveals cetuximab and bevacizumab response patterns in metastatic colorectal cancer	wrong population	round I
rayyan-661154617	"Pathological Features	wrong population	round I
rayyan-661154618	Cost-effectiveness of pembrolizumab in first-line for microsatellite-instability-high or mismatch-repair-deficient metastatic colorectal cancerF	wrong population	round I
rayyan-661154619	Toripalimab With or Without Celecoxib as Neoadjuvant Therapy in Resectable dMMR/MSI-H Colon Cancer	wrong population	round I
rayyan-661154620	Alcohol intake and colorectal cancer risk by molecularly defined subtypes in a prospective study of older women	wrong population	round I
rayyan-661154621	Deficient mismatch repair system in patients with sporadic advanced colorectal cancer	wrong population	round I
rayyan-661154622	P-35 Zanzalintinib (XL092) in combination with atezolizumab for previously treated metastatic colorectal cancer	wrong population	round I
rayyan-661154623	Pilot study: high dose fish oil in colorectal cancer (CRC) prevention in patients with lynch syndrome	wrong population	round I
rayyan-661154624	"A Study of Nivolumab	wrong population	round I
rayyan-661154625	O-8 Final overall survival for the phase 3 KN177 study: pembrolizumab versus chemotherapy in microsatellite instability-high/mismatch repair deficient metastatic colorectal cancer	wrong population	round I
rayyan-661154627	P-27 Phase 2 study of pembrolizumab-based combination therapy in patients with microsatellite instability-high or mismatch repair-deficient stage IV colorectal cancer	wrong population	round I
rayyan-661154628	"Single-nucleotide variants	wrong population	round I
rayyan-661154629	Pembrolizumab vs chemotherapy in patients with microsatellite instability-high/mismatch repair deficient metastatic colorectal cancer: asia subgroup results of the phase III KEYNOTE-177 study	wrong population	round I
rayyan-661154630	The EMA assessment of pembrolizumab as monotherapy for the first-line treatment of adult patients with metastatic microsatellite instability-high or mismatch repair deficient colorectal cancer	wrong population	round I
rayyan-661154631	Avelumab versus standard second line treatment chemotherapy in metastatic colorectal cancer patients with microsatellite instability: the SAMCO-PRODIGE 54 randomised phase II trial	wrong population	round I
rayyan-661154632	"Comparative Effectiveness of Immune Checkpoint Inhibitors vs Chemotherapy in Patients With Metastatic Colorectal Cancer With Measures of Microsatellite Instability	wrong population	round I
rayyan-661154634	EE416 Cost-Effectiveness of Pembrolizumab Monotherapy for First-Line Treatment in Adult Patients with Microsatellite Instability High/Mismatch Repair-Deficient Metastatic Colorectal Cancer From a French Perspective	wrong population	round I
rayyan-661154635	Perioperative Bevacizumab-based Triplet Chemotherapy in Patients With Potentially Resectable Colorectal Cancer Liver Metastases	wrong population	round I
rayyan-661154637	ESMO Congress 2021: highlights from the EORTC gastrointestinal tract cancer groupâ€™s perspective	wrong population	round I
rayyan-661154639	A Vulnerability of a Subset of Colon Cancers with Potential Clinical Utility	wrong population	round I

rayyan-661154640	Urachal carcinoma: The journey so far and the road ahead	wrong population	round I
rayyan-661154644	Precision Oncology in Pediatric Cancer Surgery	wrong population	round I
rayyan-661154646	Meeting report from the joint IARCâ€“NCI international cancer seminar series: a focus on colorectal cancer	wrong population	round I
rayyan-661154649	Immune Profiling of Deficient Mismatch Repair Colorectal Cancer Tumor Microenvironment Reveals Different Levels of Immune System Activation	wrong population	round I
rayyan-661154650	Chapter 24 - Molecular testing in lung cancer	wrong population	round I
rayyan-661154651	Colorectal carcinomas with submucosal invasion (pT1): analysis of histopathological and molecular factors predicting lymph node metastasis	wrong population	round I
rayyan-661154653	c-MET Overexpression in Colorectal Cancer: A Poor Prognostic Factor for Survival	wrong population	round I
rayyan-661154654	The landscape of genomic copy number alterations in colorectal cancer and their consequences on gene expression levels and disease outcome	wrong population	round I
rayyan-661154655	Unveiling the role of KRAS in tumor immune microenvironment	wrong population	round I
rayyan-661154657	Comprehensive genomic profiling of small bowel adenocarcinoma by tissue and plasma biopsy	wrong population	round I
rayyan-661154658	Relationship Between Thymidine Kinase 1 Expression and Trifluridine/Tipiracil Therapy in Refractory Metastatic Colorectal Cancer: A Pooled Analysis of 2 Randomized Clinical Trials	wrong population	round I
rayyan-661154659	Recommendations for the use of next-generation sequencing (NGS) for patients with metastatic cancers: a report from the ESMO Precision Medicine Working Group	wrong population	round I
rayyan-661154666	Combination therapies with HSP90 inhibitors against colorectal cancer	wrong population	round I
rayyan-661154668	Chemotherapy of metastatic colon cancer in France: A population-based study	wrong population	round I
rayyan-661154669	Integrative molecular analysis of colorectal cancer and gastric cancer: What have we learnt?	wrong population	round I
rayyan-661154670	Curcuma as an adjuvant in colorectal cancer treatment	wrong population	round I
rayyan-661154672	"Relation between expression of hMLH1 and p53 mRNA genes	wrong population	round I
rayyan-661154675	Embryonic origin of primary colon cancer predicts survival in patients undergoing ablation for colorectal liver metastases	wrong population	round I
rayyan-661154676	Current practice of genomic profiling of patients with advanced solid tumours in Italy: the Italian Register of Actionable Mutations (RATIONAL) study	wrong population	round I
rayyan-661154677	Aberrant DNA Methylation in Colorectal Cancer: What Should We Target?	wrong population	round I
rayyan-661154678	Rational development of combination therapies for biliary tract cancers	wrong population	round I
rayyan-661154679	Correlation of polypoid colorectal adenocarcinoma with pre-existing adenomatous polyps and KRAS mutation	wrong population	round I
rayyan-661154680	Temozolomide and irinotecan (TEMIRI regimen) as salvage treatment of irinotecan-sensitive advanced colorectal cancer patients bearing MGMT methylation	wrong population	round I
rayyan-661154683	The pattern of peritoneal colorectal metastasis predicts survival after cytoreductive surgery and hyperthermic intra-peritoneal chemotherapy	wrong population	round I

rayyan-661154684	Chapter 9 - Targeted therapy and drug resistance in gastric and pancreatic cancer	wrong population	round I
rayyan-661154685	LBA23 Avelumab versus standard second-line treatment chemotherapy in metastatic colorectal cancer (mCRC) patients with microsatellite instability (MSI): the SAMCO-PRODIGE 54 randomised phase II trial	wrong population	round I
rayyan-661154686	"Pembrolizumab versus chemotherapy for microsatellite instability-high or mismatch repair-deficient metastatic colorectal cancer (KEYNOTE-177): final analysis of a randomised	wrong population	round I
rayyan-661154687	Exploratory analysis of baseline microsatellite instability (MSI) status in patients with metastatic colorectal cancer (mCRC) treated with regorafenib (REG) or placebo in the phase 3 CORRECT trial	wrong population	round I
rayyan-661154689	Safety and efficacy of cobimetinib (cobi) and atezolizumab (atezo) in a Phase 1b study of metastatic colorectal cancer (mCRC)	wrong population	round I
rayyan-661154690	A Real-World Comparison of Regorafenib and Trifluridine/Tipiracil in Refractory Metastatic Colorectal Cancer in the United States	wrong population	round I
rayyan-661154691	CpG island methylator phenotype is an independent predictor of survival benefit from 5-fluorouracil in stage III colorectal cancer	wrong population	round I
rayyan-661154692	In-depth clinical and biological exploration of DNA damage immune response as a biomarker for oxaliplatin use in colorectal cancer	wrong population	round I
rayyan-661154693	PS1-2 Pembrolizumab vs chemotherapy for MSI-high/dMMR metastatic colorectal cancer: asia subgroup of phase 3 KEYNOTE-177	wrong population	round I
rayyan-661154694	Negative Hyperselection of Patients With RAS and BRAF Wild-Type Metastatic Colorectal Cancer Who Received Panitumumab-Based Maintenance Therapy	wrong population	round I
rayyan-661154695	O7-3 Pembrolizumab vs chemotherapy in MSI-H / dMMR metastatic colorectal cancer: KEYNOTE-177 final analysis Asia subgroup	wrong population	round I
rayyan-661154696	KEYSTEP-008: phase II trial of pembrolizumab-based combination in MSI-H/dMMR metastatic colorectal cancer	wrong population	round I
rayyan-661154697	A phase Ib biomarker trial of naproxen in patients at risk for DNA mismatch repair deficient colorectal cancer	wrong population	round I
rayyan-661154698	Genetic variants of methyl metabolizing enzymes and epigenetic regulators: associations with promoter CpG island hypermethylation in colorectal cancer	wrong population	round I
rayyan-661154700	Pharmacogenomic Study for Individual Response to CPT-11 in Colorectal Cancer Patients (Step 2)	wrong population	round I
rayyan-661154701	Regorafenib in Combination With Pembrolizumab or Pembrolizumab for MSI-H Colorectal Cancer	wrong population	round I
rayyan-661154702	Cost-effectiveness analysis of pembrolizumab versus chemotherapy as first-line treatment for mismatch-repair-deficient (dMMR) or microsatellite-instability-high (MSI-H) advanced or metastatic colorectal cancer from the perspective of the Chinese health-care system	wrong population	round I
rayyan-661154703	Nivo versus Nivo/Ipi or versus other chemotherapies to treat colorectal cancer	wrong population	round I
rayyan-661154704	AtezoTRIBE: a randomised phase II study of FOLFOXIRI plus bevacizumab alone or in combination with atezolizumab as initial therapy for patients with unresectable metastatic colorectal cancer	wrong population	round I
rayyan-661154705	Association of Consensus Molecular Subtypes and Molecular Markers With Clinical Outcomes in Patients With Metastatic Colorectal Cancer: biomarker Analyses From LUME-Colon 1	wrong population	round I
rayyan-661154706	Prognostic association of PTGS2 (COX-2) over-expression according to BRAF mutation status in colorectal cancer: results from two prospective cohorts and CALGB 89803 (Alliance) trial	wrong population	round I

rayyan-661154707	Effect of Combined Immune Checkpoint Inhibition vs Best Supportive Care Alone in Patients With Advanced Colorectal Cancer: the Canadian Cancer Trials Group CO.26 Study	wrong population	round I
rayyan-661154708	Predictive and prognostic value of HER2 gene expression and HER2amplification in patients with metastatic colorectal cancer (mCRC) enrolled in CALGB/SWOG 80405 (Alliance)	wrong population	round I
rayyan-661154709	HCRN GI16-288: a phase II trial of perioperative CV301 vaccination in combination with nivolumab and systemic chemotherapy for resectable hepatic-limited metastatic colorectal cancer	wrong population	round I
rayyan-661154710	"Clinical and exploratory biomarker findings from the MODUL trial (Cohorts 1	wrong population	round I
rayyan-661154712	A study on microsatellite instability status for colorectal serrated lesions	wrong population	round I
rayyan-661154713	Standard Chemotherapy vs Immunotherapie in 2nd Line Treatment of MSI Colorectal Mestastatic Cancer	wrong population	round I
rayyan-661154714	Phase IB study of vemurafenib in combination with irinotecan and cetuximab in patients with metastatic colorectal cancer with BRAFV600E mutation	wrong population	round I
rayyan-661154715	Association of high microsatellite instability (MSI-H) with a high immunoscore (IS) compared to PD-L1 expression and increased survival in patients (pts) with metastatic colorectal cancer (mCRC) treated with oxaliplatin (Ox) and fluoropyrimidine (FP): a pooled analysis of the AIO KRK 0207 and RO91 trials	wrong population	round I
rayyan-661154717	Circulating tumor DNA analysis for predictive and prognostic factors in patients with metastatic colorectal cancer: an exploratory analysis from the phase III PARADIGM study	wrong population	round I
rayyan-661154718	STELLAR-303: a phase 3 study of XL092 in combination with atezolizumab versus regorafenib in patients with previously treated metastatic colorectal cancer (mCRC)	wrong population	round I
rayyan-661154719	"Efficacy and safety results from IMblaze370	wrong population	round I
rayyan-661154720	"A global	wrong population	round I
rayyan-661154722	NRG-GI004/SWOG-S1610: colorectal cancer metastatic dMMR immuno-therapy (COMMIT) study-A randomized phase III study of atezolizumab (atezo) monotherapy versus mFOLFOX6/bevacizumab/atezo in the first-line treatment of patients (pts) with deficient DNA mismatch repair (dMMR) or microsatellite instability high (MSI-H) metastatic colorectal cancer (mCRC)	wrong population	round I
rayyan-661154725	Multicenter phase I/II trial of BBI608 and pembrolizumab combination in patients with metastatic colorectal cancer (SCOOP Study): EPOC1503	wrong population	round I
rayyan-661154728	SEAMARK: randomized phase 2 study of pembrolizumab + encorafenib + cetuximab versus pembrolizumab alone for first-line treatment of BRAF V600E-mutant and microsatellite instability-high (MSI-H)/mismatch repair deficient (dMMR) metastatic colorectal cancer (CRC)	wrong population	round I
rayyan-661154729	"A phase III study of nivolumab (NIVO)	wrong population	round I
rayyan-661154730	"Nivolumab Â± ipilimumab treatment (Tx) efficacy	wrong population	round I
rayyan-661154731	Predictive and prognostic value of microsatellite instability in patients with advanced colorectal cancer treated with a fluoropyrimidine and oxaliplatin containing first-line chemotherapy. A report of the AIO Colorectal Study Group	wrong population	round I
rayyan-661154732	"Microsatellite instability (MSI)	wrong population	round I

rayyan-661154733	"Neoadjuvant PD-1 blockade with toripalimab	wrong population	round I
rayyan-661154734	"Health-related quality of life in patients with microsatellite instability-high or mismatch repair deficient metastatic colorectal cancer treated with first-line pembrolizumab versus chemotherapy (KEYNOTE-177): an open-label	wrong population	round I
rayyan-661154735	LBA32 Pembrolizumab versus chemotherapy in microsatellite instability-high (MSI-H)/mismatch repair-deficient (dMMR) metastatic colorectal cancer (mCRC): 5-year follow-up of the randomized phase III KEYNOTE-177 study	wrong population	round I
rayyan-661154737	Health-related quality of life (HRQoL) in patients (pts) treated with pembrolizumab (pembro) vs chemotherapy as first-line treatment in microsatellite instability-high (MSI-H) and/or deficient mismatch repair (dMMR) metastatic colorectal cancer (mCRC): phase III KEYNOTE-177 study	wrong population	round I
rayyan-661154738	A Study of HX008 Compared to Chemotherapy in the First-Line Treatment of Subjects With MSI-H/dMMR Metastatic Colorectal Cancer	wrong population	round I
rayyan-661154739	BRAF V600E-mutant and MSI-H Metastatic Colorectal Cancer Study of Encorafenib Taken With Cetuximab Plus Pembrolizumab Compared to Pembrolizumab Alone	wrong population	round I
rayyan-661154740	KRAS and BRAF mutations in advanced colorectal cancer are associated with poor prognosis but do not preclude benefit from oxaliplatin or irinotecan: results from the MRC FOCUS trial	wrong population	round I
rayyan-661154741	"Association Between Baseline Circulating Tumor Cells	wrong population	round I
rayyan-661154742	AVEVAC: a phase I-II trial with avelumab plus autologous dendritic cell (ADC) vaccine in pre-treated mismatch repair-proficient (MSS) metastatic colorectal cancer (mCRC) patients (GEMCAD 16-02)	wrong population	round I
rayyan-661154743	A Study of Novel Oncology Therapies in Combination with FOLFOX and Bevacizumab in Metastatic Microsatellite-Stable Colorectal Cancer	wrong population	round I
rayyan-661154744	Hide and Seek with Hereditary Cancer: improving detection of colorectal cancer patients with a high risk of Lynch syndrome	wrong population	round I
rayyan-661154745	506TiP Pembrolizumab plus lenvatinib versus standard of care for previously treated metastatic colorectal cancer (mCRC): phase III LEAP-017 study	wrong population	round I
rayyan-661154747	Microsatellite instability and cytogenetic survey in myeloid leukemias	wrong population	round I
rayyan-661154749	"Atezolizumab with or without cobimetinib versus regorafenib in previously treated metastatic colorectal cancer (IMblaze370): a multicentre	wrong population	round I
rayyan-661154751	Local ablation of liver and lung metastases in patients with colorectal cancer after failure of first- or later-line systemic chemotherapy â€“ a prospective trial	wrong population	round I
rayyan-661154752	A Randomized Phase III Study of Anlotinib Versus Bevacizumab in Combination With CAPEOX as First-Line Therapy for RAS/BRAF Wild-Type Metastatic Colorectal Cancer: a Clinical Trial Protocol	wrong population	round I
rayyan-661154753	Staged analysis of standard of care tumor molecular testing among patients with metastatic colorectal cancer in the community health system setting	wrong population	round I
rayyan-661154755	Avelumab and cetuximab in combination with FOLFOX in patients with previously untreated metastatic colorectal cancer (MCRC): final results of the phase II AVETUX trial (AIO-KRK-0216)	wrong population	round I
rayyan-661154756	BRCA1 genetic variant to predict survival in metastatic colorectal cancer (mCRC) patients (pts) treated with FOLFIRI/bevacizumab (bev): results from phase III TRIBE and FIRE-3 trials	wrong population	round I
rayyan-661154757	"P-228 Randomized phase II trial of encorafenib and cetuximab with or without nivolumab for patients with previously treated	wrong population	round I
rayyan-661154758	P-23 Phase II study (daNIS-3) of the antiâ€“TGF-Î² monoclonal antibody NIS793 and other new investigational drug combinations with standard-of-care therapy	wrong population	round I

	vs standard-of-care alone in patients with second-line metastatic colorectal cancer		
rayyan-661154759	P-81 Phase 3 study of MK4280A (coformulated favezelimab and pembrolizumab) versus standard of care in previously treated PD-L1â€"positive metastatic colorectal cancer (mCRC)	wrong population	round I
rayyan-661154760	Microsatellite instability (MSI-H) is associated with a high immunoscore but not with PD-L1 expression or increased survival in patients (pts.) with metastatic colorectal cancer (mCRC) treated with oxaliplatin (ox) and fluoropyrimidine (FP) with and without bevacizumab (bev): a pooled analysis of the AIO KRK 0207 and RO91 trials	wrong population	round I
rayyan-661154762	"Associations of non-pedunculated T1 colorectal adenocarcinoma outcome with consensus molecular subtypes	wrong population	round I
rayyan-661154764	2020 ASCO Annual Meeting II	wrong population	round I
rayyan-661154765	Abstract Book of the 19th National Congress of Medical Oncology	wrong population	round I
rayyan-661154766	Linked color imaging improves detection rate of sessile serrated adenoma/polyp in the colon: a prospective randomized controlled trial	wrong population	round I
rayyan-661154767	"ENVASARC: a pivotal trial of envafolimab	wrong population	round I
rayyan-661154768	"Upfront FOLFOXIRI plus bevacizumab and reintroduction after progression versus mFOLFOX6 plus bevacizumab followed by FOLFIRI plus bevacizumab in the treatment of patients with metastatic colorectal cancer (TRIBE2): a multicentre	wrong population	round I
rayyan-661154769	Effectiveness of each Bethesda marker in defining microsatellite instability when screening for Lynch syndrome	wrong population	round I
rayyan-661154771	Effects of a decision support intervention on decisional conflict associated with microsatellite instability testing	wrong population	round I
rayyan-661154772	CAVE-2 (Cetuximab-AVElumab) mCRC: a Phase II Randomized Clinical Study of the Combination of Avelumab Plus Cetuximab as a Rechallenge Strategy in Pre-Treated RAS/BRAF Wild-Type mCRC Patients	wrong population	round I
rayyan-661154773	Electronic reminders for pathologists promote recognition of patients at risk for Lynch syndrome: cluster-randomised controlled trial	wrong population	round I
rayyan-661154774	"RELATIVITY-123: a phase 3	wrong population	round I
rayyan-661154775	"Phase 2	wrong population	round I
rayyan-661154776	FDA Approval Summary: pembrolizumab for the First-line Treatment of Patients with MSI-H/dMMR Advanced Unresectable or Metastatic Colorectal Carcinoma	wrong population	round I
rayyan-661154777	"A multicenter randomized phase 2 study to compare the efficacy and safety of avelumab versus standard second line treatment	wrong population	round I
rayyan-661154782	Distinct clinical and genetic mutation characteristics in sporadic and Lynch syndrome-associated endometrial cancer in a Chinese population	wrong population	round I
rayyan-661154785	Shanghai international consensus on diagnosis and comprehensive treatment of colorectal liver metastases (version 2019)	wrong population	round I
rayyan-661154786	Total neoadjuvant therapy in rectal cancer: the evidence and expectations	wrong population	round I
rayyan-661154790	Coiled-coil domain-containing 154 promotes colorectal cancer proliferation and metastasis via interacting with minichromosome maintenance complex component 2	wrong population	round I
rayyan-661154793	Advances in Targeted Treatments for NSCLC (Excluding EGFR/ALK/ROS-1/K-Ras)	wrong population	round I

rayyan-661154794	Racial differences in colorectal cancer survival at a safety net hospital	wrong population	round I
rayyan-661154795	Chapter 9 - Rac1b: An emerging therapeutic target for chemoresistance in colorectal cancer	wrong population	round I
rayyan-661154798	The location of the primary colon cancer has no impact on outcomes in patients undergoing cytoreductive surgery for peritoneal metastasis	wrong population	round I
rayyan-661154805	Biomarkers and microsatellite instability analysis of curettings can predict the behavior of FIGO stage I endometrial endometrioid adenocarcinoma	wrong population	round I
rayyan-661154806	Chapter 46 - Signaling pathways in CRC	wrong population	round I
rayyan-661154807	The genetic factors associated with Wnt signaling pathway in colorectal cancer	wrong population	round I
rayyan-661154808	Chapter 2 - Translational epigenetics in precision medicine of colorectal cancer	wrong population	round I
rayyan-661154809	Chapter 89 - Vitamin D and colorectal cancer	wrong population	round I
rayyan-661154811	Efficacy and Safety of Aflibercept in Combination With Chemotherapy Beyond Second-Line Therapy in Metastatic Colorectal Carcinoma Patients: An AGEO Multicenter Study	wrong population	round I
rayyan-661154815	Genomic and transcriptomic insights into the precision treatment of pulmonary enteric adenocarcinoma	wrong population	round I
rayyan-661154821	An Update on the Current and Emerging Targeted Agents in Metastatic Colorectal Cancer	wrong population	round I
rayyan-661154825	Pan-tropomyosin receptor kinase immunohistochemistry is a feasible routine screening strategy for NTRK fusions in mismatch repair-deficient colorectal carcinomas	wrong population	round I
rayyan-661154826	Wnt/Î²-catenin signaling in colorectal cancer: Is therapeutic targeting even possible?	wrong population	round I
rayyan-661154829	"Outcomes of Patients With Colorectal Liver Metastasis in the Developing World: Is Liver Transplantation for Unresectable Liver Metastasis	wrong population	round I
rayyan-661154831	"Prognosis and oncogenomic profiling of patients with tropomyosin receptor kinase fusion cancer in the 100	wrong population	round I
rayyan-661154832	A perspective on medicinal chemistry approaches towards adenomatous polyposis coli and Wnt signal based colorectal cancer inhibitors	wrong population	round I
rayyan-661154834	Ferroptosis in colorectal cancer: Potential mechanisms and effective therapeutic targets	wrong population	round I
rayyan-661154839	Traditional serrated adenoma with BRAF mutation is associated with synchronous/metachronous BRAF-mutated serrated lesions	wrong population	round I
rayyan-661154840	Prognostic value of desmoplastic reaction characterisation in stage II colon cancer: prospective validation in a Phase 3 study (SACURA Trial)	wrong population	round I
rayyan-661154841	Clinical and Molecular Biologic Characteristics of Early-onset Versus Late-onset Colorectal Carcinoma in Filipinos	wrong population	round I
rayyan-661154842	Assessing tumor mutations to gain insight into base excision repair sequence polymorphisms and smoking in colon cancer	wrong population	round I
rayyan-661154843	Impact of high-risk features for stage II adenocarcinoma of the appendix	wrong population	round I
rayyan-661154844	Camrelizumab and apatinib combined with chemotherapy (mFOLFOX6) as neoadjuvant therapy for locally advanced right-sided colon cancer (ambition)	wrong population	round I
rayyan-661154848	The Drug Rediscovery protocol facilitates the expanded use of existing anticancer drugs	wrong population	round I
rayyan-661154850	Antitumor Activity and Safety of Dostarlimab Monotherapy in Patients With Mismatch Repair Deficient Solid Tumors: a Nonrandomized Controlled Trial	wrong population	round I

rayyan-661154851	Phase 2 Basket study of Pembrolizumab or MK1308A in mCRC	wrong population	round I
rayyan-661154852	A clinical trial to evaluate the mechanism of action and the safety of the cancer vaccine L-BLP25 in rectal cancer subjects undergoing treatment with chemotherapy and radiotherapy	wrong population	round I
rayyan-661154853	The Rome Trial From Histology to Target: the Road to Personalize Target Therapy and Immunotherapy	wrong population	round I
rayyan-661154854	Clinical Trial Evaluating FOLFIRI + Durvalumab vs FOLFIRI + Durvalumab and Tremelimumab in Second-line Treatment of Patients With Advanced Gastric or Gastro-oesophageal Junction Adenocarcinoma	wrong population	round I
rayyan-661154855	Endoscopic Surveillance in Serrated Polyposis Syndrome and Low-risk of Advanced Neoplasia	wrong population	round I
rayyan-661154981	Early screening for colorectal cancer via a simple blood sample	wrong population	round I
rayyan-661154982	Comparative analysis of the protective effect of different screening strategies for colorectal cancer	wrong population	round I
rayyan-661154983	Intestinal cancer and the risk for other cancer in the same person	wrong population	round I
rayyan-661154984	Could supported weight loss reduce bowel cancer surgery complications?	wrong population	round I
rayyan-661154985	Exercise prehabilitation in colorectal cancer	wrong population	round I
rayyan-661154988	Quality of life after surgery and other options to prevent cancer of the lining of the womb (endometrial cancer)	wrong population	round I
rayyan-661155008	COLO-DETECT: Can an artificial intelligence device increase detection of polyps during colonoscopy?	wrong population	round I
rayyan-661155009	The effect of a dietary intervention with rice bran on intestinal health among adults at high risk of colorectal cancer	wrong population	round I
rayyan-661155010	"Scaling Colorectal cancer screening through Outreach	wrong population	round I
rayyan-661155011	Planning using MRI for anal and rectal cancer radiotherapy treatment	wrong population	round I
rayyan-661155012	"A randomised multicenter clinical trial for patients with multi-organ	wrong population	round I
rayyan-661155014	APHRODITE - A phase II trial of higher radiotherapy dose in the eradication of early rectal cancer	wrong population	round I
rayyan-661155015	Participation in colorectal cancer screening among asymptomatic average-risk Chinese aged 50 to 75: A population-based survey	wrong population	round I
rayyan-661155016	Testing the effectiveness of a new screening system for colorectal cancer screening: a community trial	wrong population	round I
rayyan-661155018	Can neurotensin and IL-8 levels in blood be used to identify colorectal (large bowel) cancer and adenomas (polyps)?	wrong population	round I
rayyan-661155019	Detection of bowel cancer using urinary biomarkers	wrong population	round I
rayyan-661155020	Evaluation of colon cancer screening on colon cancer mortality	wrong population	round I
rayyan-661155021	Yorkshire Cancer Research Bowel Cancer Improvement Program	wrong population	round I
rayyan-661155022	Psychological determiners of distincts fatigue trajectories in colorectal patients undergoing chemotherapy	wrong population	round I
rayyan-661155023	A decision aid for helping people to decide about colorectal cancer screening	wrong population	round I

rayyan-661155024	Short term Water-only Fasting prior to chemotherapy Trial (SWiFT)	wrong population	round I
rayyan-661155025	Comparing two optical technologies when screening for bowel cancer: a trial from several UK hospitals	wrong population	round I
rayyan-661155026	Handicraft classes for colorectal cancer patients with chemotherapy-induced neuropathy: improving neuropathic symptoms in hands through exercising the hand muscles	wrong population	round I
rayyan-661155027	Effect of a family-based multimedia program on the utilisation rate of colorectal cancer screening among South Asian older adults in Hong Kong	wrong population	round I
rayyan-661155028	Does intravenous lidocaine speed up gut recovery after large bowel surgery?	wrong population	round I
rayyan-661155029	Factors impacting on prognosis and treatment in patients with liver and lung metastases from colorectal cancer	wrong population	round I
rayyan-661155030	Raman spectroscopy and colorectal cancer	wrong population	round I
rayyan-661155031	FITâ€" Can a Dipstick Test Rule Out Bowel Cancer?	wrong population	round I
rayyan-661155032	Trifluridine/tipiracil (FTD/TPI) quality of life study in mCRC patients	wrong population	round I
rayyan-661155033	Personalised risk information and its impact on informed choice and intention to undergo colonoscopy in the Scottish Bowel Screening Programme	wrong population	round I
rayyan-661155034	Saliva to predict risk of disease using transcriptomics and epigenetics	wrong population	round I
rayyan-661155036	Targeting PI3K Signaling as a Therapeutic Approach for Colorectal Cancer	wrong population	round I
rayyan-661155038	Association of baseline absolute neutrophil counts and survival in patients with metastatic colorectal cancer treated with second-line antiangiogenic therapies: exploratory analyses of the RAISE trial and validation in an electronic medical record data set	wrong population	round I
rayyan-661155041	Preliminary Analysis of Liquid Biopsy after Hepatectomy for Colorectal Liver Metastases	wrong population	round I
rayyan-661155046	External validation of COMPASS and BIOSCOPE prognostic scores in colorectal peritoneal metastases treated with cytoreductive surgery and hyperthermic intraperitoneal chemotherapy (HIPEC)	wrong population	round I
rayyan-661155052	Ataxia Telangiectasia Mutated Protein Loss and Benefit From Oxaliplatin-based Chemotherapy in Colorectal Cancer	wrong population	round I
rayyan-661155054	"Genomic characterization of metastatic patterns from prospective clinical sequencing of 25	wrong population	round I
rayyan-661155055	Primary Tumor Location and Survival in the General Population With Metastatic Colorectal Cancer	wrong population	round I
rayyan-661155057	ESMO consensus guidelines for the management of patients with metastatic colorectal cancer	wrong population	round I
rayyan-661155059	Tumour markers in colorectal cancer: European Group on Tumour Markers (EGTM) guidelines for clinical use	wrong population	round I
rayyan-661155060	Gene heterogeneity in metastasis of colorectal cancer to the lung	wrong population	round I
rayyan-661155062	"Molecular markers and biological targeted therapies in metastatic colorectal cancer: expert opinion and recommendations derived from the 11th ESMO/World Congress on Gastrointestinal Cancer	wrong population	round I
rayyan-661155064	Liver transplantation for non-resectable colorectal liver metastases: the International Hepato-Pancreato-Biliary Association consensus guidelines	wrong population	round I

rayyan-661155065	Acquired resistance to EGFR-targeted therapies inÂ colorectal cancer	wrong population	round I
rayyan-661155066	"TAS-102 plus bevacizumab for patients with metastatic colorectal cancer refractory to standard therapies (C-TASK FORCE): an investigator-initiated	wrong population	round I
rayyan-661155072	Higher prevalence of KRAS mutations in colorectal cancer in Saudi Arabia: Propensity for lung metastasis	wrong population	round I
rayyan-661155076	Lung Metastasis Predicts Better Prognosis in Metastatic Colorectal Cancer With Mutated KRAS	wrong population	round I
rayyan-661155078	Oncologic Outcomes in Metastatic Colorectal Cancer with Regorafenib with FOLFIRI as a Third- or Fourth-Line Setting	wrong population	round I
rayyan-661155080	"Pan-Asian adapted ESMO consensus guidelines for the management of patients with metastatic colorectal cancer: a JSMOâ€“ESMO initiative endorsed by CSCO	wrong population	round I
rayyan-661155082	Emerging paradigms in the treatment of liver metastases in colorectal cancer	wrong population	round I
rayyan-661155083	NTRK Fusion in a Cohort of BRAF p. V600E Wild-Type Papillary Thyroid Carcinomas	wrong population	round I
rayyan-661155084	Treatment of Liver Metastases With Focused Ultrasound and Microbubbles in Patients With Colorectal Cancer Receiving Chemotherapy	wrong population	round I
rayyan-661155085	A Study on the accuracy and development of best practices using fluorescence for precision surgery in colorectal cancer	wrong population	round I
rayyan-661155086	IntAct- IFA to prevent anastomotic leak in rectal cancer surgery	wrong population	round I
rayyan-661155087	Investigating the use of a new fluorescent compound to identify pre cancerous and cancerous lesions in the bowel	wrong population	round I
rayyan-661155088	The CReST2 Trial: Are uncovered or covered stents more effective in relieving bowel obstruction in people with colorectal cancer	wrong population	round I
rayyan-661155089	Cancer detection by analysing non-invasively collected colorectal mucus	wrong population	round I
rayyan-661155090	Study of efficacy and safety of S 95005 (TAS-102) in patients with metastatic colorectal cancer who failed standard chemotherapies	wrong population	round I
rayyan-661155091	Supportive exercise programmes for accelerating recovery after major abdominal cancer surgery	wrong population	round I
rayyan-661155092	Assessing the impact of a new fit test in the context of a population based organized screening programme for colorectal cancer	wrong population	round I
rayyan-661155093	Chemo-immunotherapy before and after surgery for peritoneal metastases of large bowel cancer	wrong population	round I
rayyan-661155094	Phase I study of S 95005 in combination with oxaliplatin in metastatic colorectal cancer	wrong population	round I
rayyan-661155095	Measuring omega-3 fatty acid levels in terminal ileal content following four weeks of omega-3 fatty acid supplementation in patients with a temporary ileostomy	wrong population	round I
rayyan-661155096	A personal electronic health record (PEPA)	wrong population	round I
rayyan-661155097	The MAGENTA trial: The molecular biology of metastatic cancer	wrong population	round I
rayyan-661155100	Assessing the additional benefit of an innovative imaging system for colonoscopy	wrong population	round I
rayyan-661155101	General practice endorsement in the Bowel Cancer Screening Programme	wrong population	round I
rayyan-661155102	A trial to establish whether laser treatment of anal precancer prevents development of anal cancer in HIV-positive men who have sex with men	wrong population	round I

rayyan-661155103	MEK and MET Inhibition in Colorectal Cancer	wrong population	round I
rayyan-661155104	Comparison of precancerous lesions detection between standard colonoscopy and novel balloon colonoscopy	wrong population	round I
rayyan-661155105	PPALM - Palm oil and Pentoxifylline Against Late Morbidity	wrong population	round I
rayyan-661155106	Comparison of intervals between colonoscopic examinations in Familial Colorectal Cancer: The Dutch FAmilial ColorecTal cancer Surveillance study (the FACTS study) Group	wrong population	round I
rayyan-661155107	ACTION: cancer patient involvement in medical decision making	wrong population	round I
rayyan-661155108	eRAPID Electronic patient self-Reporting of Adverse-events: Patient Information and aDvice: randomised controlled trial in systemic cancer treatment	wrong population	round I
rayyan-661155109	A randomised controlled trial examining the effectiveness of a STOMA psychosocial intervention programme on the outcomes of colorectal patients with a stoma	wrong population	round I
rayyan-661155110	Assessing accuracy of PillCam Colon 2 in a screening setting: a prospective study in Italy	wrong population	round I
rayyan-661155111	Effect of exercise on insulin resistance and adiponectin in colorectal cancer survivors	wrong population	round I
rayyan-661155112	Safety and tolerability of Capecitabine and Aflibercept in patients with unresectable metastatic colorectal cancer deemed unsuitable for doublet/ triplet chemotherapy	wrong population	round I
rayyan-661155113	Effect of exercise on fitness in colorectal cancer survivors	wrong population	round I
rayyan-661155114	Clinical trial comparing epidurals to rectus sheath catheters for pain relief following major abdominal surgery	wrong population	round I
rayyan-661155115	Biomarkers Of Colorectal cancer After Bariatric Surgery	wrong population	round I
rayyan-661155116	"A phase II double-blind	wrong population	round I
rayyan-661155118	PARC (Promoting Adhesion to Referral for Colonoscopy) study: to compare different ways to invite subjects with a positive fecal occult blood test to colonoscopy	wrong population	round I
rayyan-661155119	What carcinoembryonic antigen (CEA) level should trigger further investigation during colorectal cancer follow-up?	wrong population	round I
rayyan-661155120	Estimating uptake of colorectal cancer screening tests	wrong population	round I
rayyan-661155122	CRIB (Cancer Rehabilitation In Bowel cancer): the use of cardiac rehabilitation services to aid the recovery of colorectal cancer patients: a pilot randomised controlled trial (RCT) with embedded feasibility study	wrong population	round I
rayyan-661155123	Study on narrow band imaging versus conventional colonoscopy for polyps detection in patients with positive fecal occult blood test undergoing colonoscopy screening	wrong population	round I
rayyan-661155124	Invitation strategies for colorectal (CRC) screening programmes - The impact of an advance notification letter	wrong population	round I
rayyan-661155125	Strategies to reduce the social gradient in bowel cancer screening: the ASCEND project	wrong population	round I
rayyan-661155126	Systemic chemotheRapy and the liVEr-fiRSt approach compared to index colorectal resection for colorectal cancer presenting with synchronous liver metastases (the RVERS trial)	wrong population	round I

rayyan-661155127	Hughes Abdominal Repair Trial - abdominal wall closure techniques to reduce incidence of incisional hernias	wrong population	round I
rayyan-661155128	Streamlining Staging of Colorectal cancer with Whole Body MRI	wrong population	round I
rayyan-661155129	"Adjuvant therapy: colorectal cancer chemotherapy study (LIitÃ¤nnÃ¤ishoito: Paksu- ja PerÃ¤suoliSYÃ¶vÃ¤n SYtostaattiTerapiaTutkimus): comparison of 5-fluorouracil (5-FU) and leucovorin as bolus injection or continuous infusion with special emphasis on toxicity	wrong population	round I
rayyan-661155130	Dexamethasone Reduces Emesis After Major gastrointestinal Surgery (DREAMS trial)	wrong population	round I
rayyan-661155131	Surgical timing after radiotherapy for rectal cancer	wrong population	round I
rayyan-661155132	A Phase II study of chemotherapy given before radiotherapy as treatment for patients with rectal cancer	wrong population	round I
rayyan-661155133	Traditional lateral ileostomy vs percutaneous ileostomy by exclusion probe for the protection of the extraperitoneal colo-rectal anastomosis (Ileostomia laterale tradizionale vs ileostomia percutanea escludente su sonda dedicata a protezione della anastomosi colorettali extraperitoneali)	wrong population	round I
rayyan-661155134	Pre-operative oral supplementation in colorectal cancer patients	wrong population	round I
rayyan-661155137	"A phase I/IIa study combining curcumin (Curcumin C3 Complex	wrong population	round I
rayyan-661155138	Investigating the optimal scheduling of chemotherapy in patients with colorectal and liver cancer	wrong population	round I
rayyan-661155139	Improving the prediction of metastatic disease in primary colorectal cancer	wrong population	round I
rayyan-661155140	Capsule colonoscopy option increases uptake of colorectal cancer screening	wrong population	round I
rayyan-661155141	Study of colonoscopic surveillance intervals after removal of colorectal adenomas	wrong population	round I
rayyan-661155142	"A bowel management intervention for individuals following rectal cancer treatment: assessing feasibility	wrong population	round I
rayyan-661155143	The seAFOod (Systematic Evaluation of Aspirin and Fish Oil) polyp prevention trial and STOP-ADENOMA (STudy Of Prevention by Aspirin anD EPA	wrong population	round I
rayyan-661155144	TroVaxÂ® and cyclophosphamide treatment in colorectal cancer	wrong population	round I
rayyan-661155145	The SYMPTOM Study: factors influencing patient appraisal of symptoms	wrong population	round I
rayyan-661155147	Wnt signalling in colon cancer	wrong population	round I
rayyan-661155148	A pilot study to test the effects of physical activity consultations on the physical activity levels and other health outcomes of people living with colorectal cancer and their partners	wrong population	round I
rayyan-661155149	BeWEL: the impact of a bodyweight and physical activity intervention on adults at risk of developing colorectal adenomas	wrong population	round I
rayyan-661155150	Effects of mutations in KRAS and BRAF gene and histological parameters on the clinical course of disease in patients with metastatic colorectal cancer	wrong population	round I
rayyan-661155151	"The effect of red meat consumption on the formation of N-nitroso compounds	wrong population	round I
rayyan-661155152	Italian multicentre randomised controlled trial of 'once-only sigmoidoscopy'	wrong population	round I

rayyan-661155153	Safety assessment of treatment with bevacizumab in metastatic colorectal cancer	wrong population	round I
rayyan-661155154	"Feasibility of a personalised	wrong population	round I
rayyan-661155155	Monitoring of treatment with bevacizumab in patients with metastatic colorectal cancer	wrong population	round I
rayyan-661155156	Identifying biomarkers to predict clinical benefit in patients with colorectal cancer treated with bevacizumab	wrong population	round I
rayyan-661155157	Diet and exercise and high risk adenomas	wrong population	round I
rayyan-661155158	Public perceptions of bowel cancer screening	wrong population	round I
rayyan-661155159	Detection of bowel disease using cells from the colon	wrong population	round I
rayyan-661155160	Mailing of the Faecal Occult Blood Test (FOBT) to increase compliance to colorectal cancer screening and to reduce costs	wrong population	round I
rayyan-661155161	Erbitux in preoperative chemo-radiotherapy followed by excisional surgery	wrong population	round I
rayyan-661155162	"Radiotherapy	wrong population	round I
rayyan-661155163	"EXCITE: Erbitux	wrong population	round I
rayyan-661155164	Safety assessment of treatment with cetuximab in metastatic colorectal cancer patients	wrong population	round I
rayyan-661155165	In vivo response monitoring of treatment with the epidermal growth factor receptor (EGFR) monoclonal antibody cetuximab in metastatic colorectal cancer	wrong population	round I
rayyan-661155166	Colonic curcumin tissue levels in patients awaiting colorectal endoscopy or patients with colorectal cancer awaiting resection	wrong population	round I
rayyan-661155167	Epidural Analgesic Therapy (EAT) versus IntraVenous patient-controlled Analgesia (IVA)	wrong population	round I
rayyan-661155168	Protein acetylation as a diet-modifiable biomarker of colorectal cancer risk	wrong population	round I
rayyan-661155169	"FOCUS 3 - the feasibility of molecular selection of therapy using KRAS	wrong population	round I
rayyan-661155170	Fluorouracil and folinic acid (FOLFIRI) plus an endothelin receptor antagonist (ERAT) in advanced colorectal cancer patients who have failed on oxaliplatin-containing chemotherapy	wrong population	round I
rayyan-661155171	The influence of two policies of closure of the laparotomy wound in the rate of surgical site infection in colorectal cancer surgery: the New Operation trial	wrong population	round I
rayyan-661155172	ISAAC: a randomised trial for patients with asymptomatic advanced colorectal cancer to look at the benefits of undergoing surgical removal of their primary tumour before receiving chemotherapy for metastatic disease	wrong population	round I
rayyan-661155173	Study on the benefits of fluid restriction on the recovery from surgery	wrong population	round I
rayyan-661155174	Prospective randomised clinical trial phase II: 5-fluorouracil/folinic acid (5-FU/FA) and irinotecan versus combination cepecitabin and irinotecan in patients with metastatic colorectal cancer as first line treatment	wrong population	round I
rayyan-661155175	"FOXFIRE: an open-label randomised phase III trial of 5-Fluorouracil	wrong population	round I
rayyan-661155176	Colon cancer detection by measuring DNA of cells collected from rectum: A case-control study	wrong population	round I

rayyan-661155177	The role of endoluminal stenting in the acute management of obstructing colorectal cancer	wrong population	round I
rayyan-661155179	Traditional Chinese medicine (TCM) for cancer	wrong population	round I
rayyan-661155180	CINATRA: Chromosomal Instability and Anti-Tubulin Response Assessment	wrong population	round I
rayyan-661155181	Early diagnosis of colorectal cancer by faecal occult blood detection - the Goteborg trial	wrong population	round I
rayyan-661155182	Conventional versus laparoscopic surgery for colorectal cancer within an Enhanced Recovery program	wrong population	round I
rayyan-661155183	Screening Or NO Screening: differences in survival during follow-up after random colorectal cancer screening with faecal occult blood test or no screening	wrong population	round I
rayyan-661155184	Implementation of colorectal cancer screening with Faecal Occult Blood Test (FOBT) in the Netherlands	wrong population	round I
rayyan-661155186	"Pre-operative short-course radiotherapy versus neoadjuvant radiochemotherapy in locally advanced rectal cancer (uT2N+	wrong population	round I
rayyan-661155187	Evaluation of the diagnostic quality of manganese chloride tetrahydrate (CMC-001Â©) in liver magnetic resonance imaging in patients with liver metastases in comparison to gadolinium benzyloxypropionictetraacetate (BOPTA): a randomised cross-over phase III trial	wrong population	round I
rayyan-661155188	"A phase II study of up-front red blood cell transfusion before chemotherapy followed by maintenance Erythropoetin-alpha subcutaneous support during chemotherapy of anaemic breast-	wrong population	round I
rayyan-661155189	Video autofluorescence imaging for detection of adenomatous POLyps of the colon in intermediate and high-risk PAtieNts	wrong population	round I
rayyan-661155190	A two-arm phase II randomised trial of intermittent chemotherapy plus continuous cetuximab and of intermittent chemotherapy plus intermittent cetuximab in first line treatment of patients with K-ras-normal (wild-type) metastatic colorectal cancer	wrong population	round I
rayyan-661155191	p53 immunotherapy in patients treated for metastasised colorectal cancer	wrong population	round I
rayyan-661155192	The influence of methylenetetrahydrofolate reductase C677T genotype and folate status on deoxyribonucleic acid methylation and uracil misincorporation in the colon of healthy volunteers	wrong population	round I
rayyan-661155193	"Comparing colonoscopy	wrong population	round I
rayyan-661155194	A prospective randomised open label trial of oxaliplatin/fluoropyrimidine versus oxaliplatin/fluoropyrimidine plus cetuximab pre- and post-operatively in patients with resectable colorectal liver metastasis requiring chemotherapy	wrong population	round I
rayyan-661155195	An evaluation of an Advanced Symptom Management System to monitor and manage chemotherapy-related toxicity	wrong population	round I
rayyan-661155196	"A multicentre study using the chemotherapy combination of bi-monthly Xeloda and Eloxatin	wrong population	round I
rayyan-661155197	Influence of laparoscopy and/or fast-track multimodal management on gastrointestinal motility in comparison to open surgery and/or standard care	wrong population	round I
rayyan-661155198	Relevance of circulating tumour cells in patients undergoing laparoscopic colon resection versus open resection: a randomised trial	wrong population	round I
rayyan-661155199	Influence of MLH1 gene on anti-neoplastic effects of Resistant Starch	wrong population	round I
rayyan-661155200	FOCUS 2: Drug treatment for bowel cancer - making the best choices when a milder treatment is needed	wrong population	round I

rayyan-661155201	Is blinding the endoscopists to bowel preparations in randomised controlled trials a reality?	wrong population	round I
rayyan-661155202	A blended knowledge translation initiative to improve colorectal cancer staging	wrong population	round I
rayyan-661155203	A randomised controlled study into the effect of an audiovisual intervention on patient recruitment to cancer clinical trials	wrong population	round I
rayyan-661155204	Trying to improve the compliance to colorectal cancer screening: type of test provider (GP versus hospital) and type of faecal occult blood test (Guaiac versus immunochemical)	wrong population	round I
rayyan-661155205	"Influence of intraperitoneal application of taurolidine on the perioperative metastases	wrong population	round I
rayyan-661155206	PACT (Patient Preferences in Adjuvant Colorectal Cancer Therapy): a randomised crossover clinical trial comparing bolus fluorouracil/leucovorin to capecitabine as treatment for moderate to high risk resected colorectal cancer	wrong population	round I
rayyan-661155207	Influence of two different resection techniques of liver metastases from colorectal cancer on haematogenous tumour cell dissemination	wrong population	round I
rayyan-661155208	"PICCOLO Trial: Panitumumab	wrong population	round I
rayyan-661155209	A Phase III trial comparing either COntinuous chemotherapy plus cetuximab or INtermittent chemotherapy with standard continuous palliative combination chemotherapy with oxaliplatin and a fluoropyrimidine in first line treatment of metastatic colorectal cancer	wrong population	round I
rayyan-661155210	Cost-effectiveness of intensive versus no scheduled follow-up in patients who have undergone resection for colorectal cancer with curative intent - main trial	wrong population	round I
rayyan-661155211	"Inter-individual variation in susceptibility to colorectal neoplasia: the interaction between diet	wrong population	round I
rayyan-661155212	Evaluation of guidelines for open access flexible sigmoidoscopy	wrong population	round I
rayyan-661155213	Multicentre international study of capecitabine Â± bevacizumab as adjuvant treatment of colorectal cancer	wrong population	round I
rayyan-661155214	Randomised phase III study of the local treatment of liver metastases by radiofrequency combined with chemotherapy versus chemotherapy alone in patients with unresectable colorectal liver metastases	wrong population	round I
rayyan-661155215	Cluster randomised controlled trial of computer decision support for management of familial cancer in primary care	wrong population	round I
rayyan-661155216	A Trial to Establish the Role of Leukocyte Concentration and Tissue Type of Transfused Blood in Immunomodulation of Patients Undergoing Elective Surgery for Colorectal Cancer	wrong population	round I
rayyan-661155217	A randomised trial comparing 8 cycles of CPT11 with CPT11 until disease progression in patients with colorectal cancer resistant to 5-fluorouracil	wrong population	round I
rayyan-661155218	Post-Operative Radiotherapy for Selected High Risk Rectal Adenocarcinoma	wrong population	round I
rayyan-661155220	The effect of rofecoxib on colorectal liver metastases	wrong population	round I
rayyan-661155221	A randomised controlled trial of colorectal polyp and cancer prevention using aspirin and resistant starch in carriers of hereditary nonpolyposis colorectal cancer	wrong population	round I
rayyan-661155222	Adjuvant X-ray & 5-Fluorouracil (5-FU) Infusion Study	wrong population	round I
rayyan-661155223	QUASAR (stands for QUick and Simple And Reliable). UKCCCR randomised study of adjuvant chemotherapy in colon and rectal cancer	wrong population	round I

rayyan-661155224	A randomised controlled trial of faecal occult blood screening for colorectal cancer	wrong population	round I
rayyan-661155225	A randomised trial of intravenous versus intrahepatic arterial 5-Flurouracil (5-FU) and leucovorin for colorectal liver metastases	wrong population	round I
rayyan-661155226	Multicentre randomised trial of 'once only' flexible sigmoidoscopy screening for prevention of bowel cancer morbidity and mortality	wrong population	round I
rayyan-661155227	A randomised controlled trial of laparoscopic surgery for colorectal cancer	wrong population	round I
rayyan-661155228	A randomised trial assessing the role of two new agents in the management of advanced colorectal cancer	wrong population	round I
rayyan-661155229	Chemotherapy choices in advanced colorectal cancer - a randomised trial comparing two durations and three chemotherapy regimens in the palliative treatment of advanced colorectal cancer	wrong population	round I
rayyan-661155231	Colorectal Liver Metastases: Does the Future of Precision Medicine Lie in Genetic Testing?	wrong population	round I
rayyan-661155232	Regorafenib Is Associated With Increased Skeletal Muscle Loss Compared to TAS-102 in Metastatic Colorectal Cancer	wrong population	round I
rayyan-661155233	The prognostic impact of consensus molecular subtypes (CMS) and its predictive effects for bevacizumab benefit in metastatic colorectal cancer: molecular analysis of the AGITG MAX clinical trial	wrong population	round I
rayyan-661155235	Predictive Molecular Classifiers in Colorectal Cancer	wrong population	round I
rayyan-661155239	Thyroid hormones ratio is a major prognostic marker in advanced metastatic colorectal cancer: Results from the phase III randomised CORRECT trial	wrong population	round I
rayyan-661155240	Optimal detection of clinically relevant mutations in colorectal carcinoma: sample pooling overcomes intra-tumoral heterogeneity	wrong population	round I
rayyan-661155241	Treatment guidelines of metastatic colorectal cancer in older patients from the French Society of Geriatric Oncology (SoFOG)	wrong population	round I
rayyan-661155248	The Link between the Multiverse of Immune Microenvironments in Metastases and the Survival of Colorectal Cancer Patients	wrong population	round I
rayyan-661155250	Colorectal cancer screening â€“ Methodology	wrong population	round I
rayyan-661155251	Chinese guidelines for the diagnosis and comprehensive treatment of colorectal liver metastases (V. 2023)	wrong population	round I
rayyan-661155256	"Epidermal growth factor receptor	wrong population	round I
rayyan-661155259	"The Unmet Needs of the Diagnosis	wrong population	round I
rayyan-661155264	Salvage regional therapy using hepatic artery infusion pump in unresectable chemotherapy resistant colorectal liver metastases	wrong population	round I
rayyan-661155266	Activity of temozolomide in patients with advanced chemorefractory colorectal cancer and MGMT promoter methylation	wrong population	round I
rayyan-661155267	The role of molecular biomarkers in outcomes and patient selection for cytoreductive surgery and hyperthermic intraperitoneal chemotherapy for peritoneal metastases of colorectal origin	wrong population	round I
rayyan-661155268	BRAF mutation detection in hairy cell leukaemia from archival haematolymphoid specimens	wrong population	round I
rayyan-661155272	Nonoperative management of the primary tumor in patients with unresectable stage IV colon cancer treated with systemic chemotherapy: Higher complication rates for left-sided colon tumors	wrong population	round I

rayyan-661155277	Prognostic impact of immune-microenvironment in colorectal liver metastases resected after triplets plus a biologic agent: A pooled analysis of five prospective trials	wrong population	round I
rayyan-661155279	Performance of prognostic models incorporating KRAS mutation status to predict survival after resection of colorectal liver metastases	wrong population	round I
rayyan-661155281	Rigosertib promotes anti-tumor immunity via autophagic degradation of PD-L1 in colorectal cancer cells	wrong population	round I
rayyan-661155283	Association Between Height and Clinical Outcome in Metastatic Colorectal Cancer Patients Enrolled Onto a Randomized Phase 3 Clinical Trial: Data From the FIRE-3 Study	wrong population	round I
rayyan-661155284	"Elaboration of NTRK-rearranged colorectal cancer: Integration of immunoreactivity pattern	wrong population	round I
rayyan-661155286	SMAD4 gene mutation predicts poor prognosis in patients undergoing resection for colorectal liver metastases	wrong population	round I
rayyan-661155287	Treatment decisions in metastatic colorectal cancer â€“ Beyond first and second line combination therapies	wrong population	round I
rayyan-661155288	Adnexal masses in patients with colorectal cancer	wrong population	round I
rayyan-661155293	Deciphering Brain Metastasis Stem Cell Properties From Colorectal Cancer Highlights Specific Stemness Signature and Shared Molecular Features	wrong population	round I
rayyan-661155294	Management of metastatic colorectal cancer patients: guidelines of the Italian Medical Oncology Association (AIOM)	wrong population	round I
rayyan-661155295	Prognostic score for synchronous metastatic rectal cancer: A real-world study	wrong population	round I
rayyan-661155297	Right or Left Primary Site of Colorectal Cancer: Outcomes From the Molecular Analysis of the AGITG MAX Trial	wrong population	round I
rayyan-661155302	Sequential Treatment With Trifluridine/Tipiracil and Regorafenib in Refractory Metastatic Colorectal Cancer Patients: An AGEO Prospective â€œReal-World Studyâ€	wrong population	round I
rayyan-661155303	Primary tumor sidedness has an impact on prognosis and treatment outcome in metastatic colorectal cancer: results from two randomized first-line panitumumab studies	wrong population	round I
rayyan-661155304	Long-term Clinical Outcome of Trastuzumab and Lapatinib for HER2-positive Metastatic Colorectal Cancer	wrong population	round I
rayyan-661155305	Colorectal cancer immunotherapy-Recent progress and future directions	wrong population	round I
rayyan-661155309	Mutational analysis of Î²-catenin and the RAS-RAF signalling pathway in early flat-type colorectal tumours	wrong population	round I
rayyan-661155310	"KRAS	wrong population	round I
rayyan-661155314	BRAF in non-small cell lung cancer (NSCLC): Pickaxing another brick in the wall	wrong population	round I
rayyan-661155316	"Chapter 10 - Colorectal cancer heterogeneity and targeted therapy: clinical implications	wrong population	round I
rayyan-661155318	Does the efficacy of regorafenib differ in chemotherapy refractory metastatic colorectal cancer patients who had mucinous pathology compared to those who had non-mucinous pathology?	wrong population	round I
rayyan-661155321	FOLFOXIRI Versus Doublet Regimens in Right-Sided Metastatic Colorectal Cancer: Focus on Subsequent Therapies and Impact on Overall Survival	wrong population	round I
rayyan-661155324	Phase II study of lenvatinib for metastatic colorectal cancer refractory to standard chemotherapy: the LEMON study (NCCH1503)	wrong population	round I

rayyan-661155326	Molecular Profiling of Patients With Advanced Colorectal Cancer: Princess Margaret Cancer Centre Experience	wrong population	round I
rayyan-661155328	Disease Modeling on Tumor Organoids Implicates AURKA as a Therapeutic Target in Liver Metastatic Colorectal Cancer	wrong population	round I
rayyan-661155332	Lack of Caudal-Type Homeobox Transcription Factor 2 Expression as a Prognostic Biomarker in Metastatic Colorectal Cancer	wrong population	round I
rayyan-661155335	Tumor sequencing of African ancestry reveals differences in clinically relevant alterations across common cancers	wrong population	round I
rayyan-661155340	Recommendations for the use of next-generation sequencing in patients with metastatic cancer in the Asia-Pacific region: a report from the APODDC working group	wrong population	round I
rayyan-661155346	"Sotorasib for previously treated colorectal cancers with KRASG12C mutation (CodeBreaK100): a prespecified analysis of a single-arm	wrong population	round I
rayyan-661155351	Primary tumor location and survival among metastatic colorectal cancer patients treated with systemic chemotherapy and biologic therapies: Retrospective analysis	wrong population	round I
rayyan-661155353	Clinical Trials and Progress in Metastatic Colon Cancer	wrong population	round I
rayyan-661155354	Integrated Omics of Metastatic Colorectal Cancer	wrong population	round I
rayyan-661155356	Prognostic and predictive value of primary tumour side in patients with RAS wild-type metastatic colorectal cancer treated with chemotherapy and EGFR directed antibodies in six randomized trialsâ€	wrong population	round I
rayyan-661155359	The relevance of primary tumour location in patients with metastatic colorectal cancer: A meta-analysis of first-line clinical trials	wrong population	round I
rayyan-661155360	Single-cell transcriptome analysis reveals T population heterogeneity and functions in tumor microenvironment of colorectal cancer metastases	wrong population	round I
rayyan-661155363	Genomic profiling of sporadic liver metastatic colorectal cancer	wrong population	round I
rayyan-661155368	"Pertuzumab plus trastuzumab for HER2-amplified metastatic colorectal cancer (MyPathway): an updated report from a multicentre	wrong population	round I
rayyan-661155373	"Melanoma with in-frame deletion of MAP2K1: a distinct molecular subtype of cutaneous melanoma mutually exclusive from BRAF	wrong population	round I
rayyan-661155374	Chapter 19 - Circulating DNA and Protein Biomarkers for the Treatment of Metastatic Colorectal Cancer with Tyrosine Kinase Inhibitors	wrong population	round I
rayyan-661155376	Biomarker-guided implementation of the old drug temozolomide as a novel treatment option for patients with metastatic colorectal cancer	wrong population	round I
rayyan-661155378	Updated Management of Colorectal Cancer Liver Metastases: Scientific Advances Driving Modern Therapeutic Innovations	wrong population	round I
rayyan-661155380	KRAS and BRAF mutations in sinonasal cancer	wrong population	round I
rayyan-661155381	Dichotomous colorectal cancer behaviour	wrong population	round I
rayyan-661155382	Clinical and molecular determinants of extrahepatic disease progression in patients with metastatic colorectal cancer with liver-limited metastases deemed initially unresectable	wrong population	round I
rayyan-661155387	Genetics and Genetic Biomarkers in Sporadic Colorectal Cancer	wrong population	round I
rayyan-661155388	The current value of determining the mismatch repair status of colorectal cancer: A rationale for routine testing	wrong population	round I
rayyan-661155389	Chapter 33 - Molecularly targeted therapy in metastatic CRC	wrong population	round I

rayyan-661155390	Chapter 34 - A roadmap for medical treatment of metastatic CRC	wrong population	round I
rayyan-661155391	Chapter 15 - Immunotherapy and targeted therapies for colorectal liver metastasis	wrong population	round I
rayyan-661155397	The impact of molecular and mismatch repair status on the survival outcomes of surgically treated patients with colorectal peritoneal metastases	wrong population	round I
rayyan-661155401	Clinical and Molecular Features in Patients Undergoing Cytoreductive Surgery and Hyperthermic Intraperitoneal Chemotherapy for Peritoneal Carcinosis from Colorectal Cancer	wrong population	round I
rayyan-661155403	Prognostic impact of performance status on the outcomes of immune checkpoint inhibition strategies in patients with dMMR/MSI-H metastatic colorectal cancer	wrong population	round I
rayyan-661155404	Resistance to anti-epidermal growth factor receptor in metastatic colorectal cancer: What does still need to be addressed?	wrong population	round I
rayyan-661155405	Trifluridine/tipiracil (FTD/TPI) and regorafenib in older patients with metastatic colorectal cancer	wrong population	round I
rayyan-661155406	Colorectal poorly differentiated neuroendocrine carcinomas frequently exhibit BRAF mutations and are associated with poor overall survival	wrong population	round I
rayyan-661155407	Response to Chemotherapy and Prognosis in Metastatic Colorectal Cancer With DNA Deficient Mismatch Repair	wrong population	round I
rayyan-661155410	Prognostic and predictive impact of consensus molecular subtypes and CRCAssigner classifications in metastatic colorectal cancer: a translational analysis of the TRIBE2 study	wrong population	round I
rayyan-661155411	Ramucirumab plus triplet chemotherapy as an alternative salvage treatment for patients with metastatic colorectal cancer	wrong population	round I
rayyan-661155414	Treatment sequencing in metastatic colorectal cancer	wrong population	round I
rayyan-661155417	"Trifluridineâ€“tipiracil plus bevacizumab versus capecitabine plus bevacizumab as first-line treatment for patients with metastatic colorectal cancer ineligible for intensive therapy (SOLSTICE): a randomised	wrong population	round I
rayyan-661155419	Prognostic Impact of Primary Tumor Location on Clinical Outcomes of Metastatic Colorectal Cancer Treated With Cetuximab Plus Oxaliplatin-Based Chemotherapy: A Subgroup Analysis of the JACCRO CC-05/06 Trials	wrong population	round I
rayyan-661155421	Efficacy of immunotherapy in mismatch repair-deficient advanced colorectal cancer in routine clinical practice. An AGEO study	wrong population	round I
rayyan-661155422	NeoRAS wild-type in metastatic colorectal cancer: Myth or truth?â€"Case series and review of the literature	wrong population	round I
rayyan-661155425	Three Rounds of External Quality Assessment in France to Evaluate the Performance of 28 Platforms for Multiparametric Molecular Testing in Metastatic Colorectal and Non-Small Cell Lung Cancer	wrong population	round I
rayyan-661155427	HER2 amplification in colorectal cancer with brain metastasis: A propensity score matching study	wrong population	round I
rayyan-661155428	Clinical utility of comprehensive circulating tumor DNA genotyping compared with standard of care tissue testing in patients with newly diagnosed metastatic colorectal cancer	wrong population	round I
rayyan-661155429	Regorafenib plus toripalimab in patients with metastatic colorectal cancer: a phase Ib/II clinical trial and gut microbiome analysis	wrong population	round I
rayyan-661155431	RAS Mutation Status Confers Prognostic Relevance in Patients Treated With Cytoreductive Surgery and Hyperthermic Intraperitoneal Chemotherapy for Colorectal Cancer	wrong population	round I
rayyan-661155432	Delineating the intra-patient heterogeneity of molecular alterations in treatment-naÃ¯ve colorectal cancer with peritoneal carcinomatosis	wrong population	round I

rayyan-661155433	Encorafenib in Combination With Cetuximab After Systemic Therapy in Patients With BRAFV600E Mutant Metastatic Colorectal Cancer: German Health Technology Assessment-Driven Analyses From the BEACON CRC Study	wrong population	round I
rayyan-661155434	FOLFOXIRI plus bevacizumab versus FOLFOX plus bevacizumab for patients with metastatic colorectal cancer and â‰¥3 circulating tumour cells: the randomised phase III VISNÃš-1 trial	wrong population	round I
rayyan-661155437	Liver Transplantation for Colorectal Liver Metastases	wrong population	round I
rayyan-661155439	Chemotherapy beyond immune checkpoint inhibitors in patients with metastatic colorectal cancer	wrong population	round I
rayyan-661155440	Maintenance treatment with capecitabine and bevacizumab versus observation in metastatic colorectal cancer: updated results and molecular subgroup analyses of the phase 3 CAIRO3 study	wrong population	round I
rayyan-661155441	FOLFOXIRI and bevacizumab in patients with early-onset metastatic colorectal cancer. A pooled analysis of TRIBE and TRIBE2 studies	wrong population	round I
rayyan-661155443	"Unified framework for patient-derived	wrong population	round I
rayyan-661155444	Microsatellite instability status affects gene expression profiles in early onset colorectal cancer patients	wrong population	round I
rayyan-661155445	Prognostic Implications of Mucinous Differentiation in Metastatic Colorectal Carcinoma Can Be Explained by Distinct Molecular and Clinicopathologic Characteristics	wrong population	round I
rayyan-661155446	Prognostic Value and Molecular Landscape of HER2 Low-Expressing Metastatic Colorectal Cancer	wrong population	round I
rayyan-661155447	Tumor mutational burden is predictive of response to immune checkpoint inhibitors in MSI-high metastatic colorectal cancer	wrong population	round I
rayyan-661155448	"Consensus molecular subtypes in metastatic colorectal cancer treated with sequential versus combined fluoropyrimidine	wrong population	round I
rayyan-661155449	"FFCD 1709-SIRTCI phase II trial: Selective internal radiation therapy plus Xelox	wrong population	round I
rayyan-661155451	Negative hyper-selection of metastatic colorectal cancer patients for anti-EGFR monoclonal antibodies: the PRESSING caseâ€“control study	wrong population	round I
rayyan-661155452	Targeting BRAF-mutant non-small cell lung cancer: Current status and future directions	wrong population	round I
rayyan-661155453	"Gender-dependent survival benefit from first-line irinotecan in metastatic colorectal cancer. Subgroup analysis of a phase III trial (XELAVIRI-study	wrong population	round I
rayyan-661155454	"Upfront FOLFOXIRI plus bevacizumab with or without atezolizumab in the treatment of patients with metastatic colorectal cancer (AtezoTRIBE): a multicentre	wrong population	round I
rayyan-661155455	Chapter 4 - Use of molecular markers and other personalized factors in treatment decisions for metastatic colorectal cancer	wrong population	round I
rayyan-661155457	Clinical efficacy of sequential treatments in KRASG12C-mutant metastatic colorectal cancer: findings from a real-life multicenter Italian study (CRC-KR GOIM)	wrong population	round I
rayyan-661155460	Long-Term Clinical Outcomes of Patients with Colorectal Cancer with Metastatic Epidural Spinal Cord Compression Treated with Hybrid Therapy (Surgery Followed by Stereotactic Body Radiation Therapy)	wrong population	round I
rayyan-661155462	Clinical Sequencing Defines the Genomic Landscape of Metastatic Colorectal Cancer	wrong population	round I
rayyan-661155467	Longitudinal change of genetic variations in cetuximab-treated metastatic colorectal cancer	wrong population	round I

rayyan-661155471	Efficacy of anti-epidermal growth factor receptor agents in patients with RAS wild-type metastatic colorectal cancerÂ â‰¥ 70 years	wrong population	round I
rayyan-661155472	Current Options for Third-line and Beyond Treatment of Metastatic Colorectal Cancer. Spanish TTD Group Expert Opinion	wrong population	round I
rayyan-661155473	The EMA assessment of encorafenib in combination with cetuximab for the treatment of adult patients with metastatic colorectal carcinoma harbouring the BRAFV600E mutation who have received prior therapy	wrong population	round I
rayyan-661155474	Predictive and Prognostic Markers in the Treatment of Metastatic Colorectal Cancer (mCRC): Personalized Medicine at Work	wrong population	round I
rayyan-661155475	Consensus statement on essential patient characteristics in systemic treatment trials for metastatic colorectal cancer: Supported by the ARCAD Group	wrong population	round I
rayyan-661155481	How we treat metastatic colorectal cancer	wrong population	round I
rayyan-661155482	"Differences in Pathology and Mutation Status Among Colorectal Cancer Patients Younger Than	wrong population	round I
rayyan-661155484	Chromatin factors: Ready to roll as biomarkers in metastatic colorectal cancer?	wrong population	round I
rayyan-661155485	Advances in Treatment Selection for Patients with Metastatic Colorectal Cancer	wrong population	round I
rayyan-661155488	Recommendations from the EGAPP Working Group: can testing of tumor tissue for mutations in EGFR pathway downstream effector genes in patients with metastatic colorectal cancer improve health outcomes by guiding decisions regarding anti-EGFR therapy?	wrong population	round I
rayyan-661155489	Real-world treatment patterns and clinical outcomes for chemotherapy-based regimens in first-line MSI-H/dMMR metastatic colorectal cancer	wrong population	round I
rayyan-661155490	Impact of BRAF and RAS mutations on first-line efficacy of FOLFIRI plus cetuximab versus FOLFIRI plus bevacizumab: analysis of the FIRE-3 (AIO KRK-0306) study	wrong population	round I
rayyan-661155491	Metastatic colorectal cancer: Advances in the folate-fluoropyrimidine chemotherapy backbone	wrong population	round I
rayyan-661155494	Dual HER2 Targeted Therapy With Pyrotinib and Trastuzumab in Refractory HER2 Positive Metastatic Colorectal Cancer: A Result From HER2-FUSCC-G Study	wrong population	round I
rayyan-661155495	Multi-omics of the gut microbial ecosystem in patients with microsatellite-instability-high gastrointestinal cancer resistant to immunotherapy	wrong population	round I
rayyan-661155496	Molecular markers of prognosis and therapeutic targets in metastatic colorectal cancer	wrong population	round I
rayyan-661155497	Association of Circulating Tumor Cells and Tumor Molecular Profile With Clinical Outcomes in Patients With Previously Untreated Metastatic Colorectal Cancer: A Pooled Analysis of the Phase III VISNÃš-1 and Phase II VISNÃš-2 Randomized Trials	wrong population	round I
rayyan-661155500	Advances in systemic chemotherapy and immunotherapy for metastatic colorectal cancer	wrong population	round I
rayyan-661155501	BRAF V600E mutations in right-side colon cancer: Heterogeneity detected by liquid biopsy	wrong population	round I
rayyan-661155504	Tumour mutational burden as a biomarker in patients with mismatch repair deficient/microsatellite instability-high metastatic colorectal cancer treated with immune checkpoint inhibitors	wrong population	round I
rayyan-661155505	Clinical-Molecular characteristics and Post-Translational modifications of colorectal cancer in north China: Implications for future targeted therapies	wrong population	round I

rayyan-661155506	KRAS inhibition in metastatic colorectal cancer: An update	wrong population	round I
rayyan-661155507	"Clinicopathologic characteristics and gene expression analyses of non-KRAS 12/13	wrong population	round I
rayyan-661155508	Impact of early tumor shrinkage and depth of response on the outcomes of panitumumab-based maintenance in patients with RAS wild-type metastatic colorectal cancer	wrong population	round I
rayyan-661155510	Metastasectomy and BRAF mutation	wrong population	round I
rayyan-661155511	Real-world first-line treatment of patients with BRAFV600E-mutant metastatic colorectal cancer: the CAPSTAN CRC studyâ˜†	wrong population	round I
rayyan-661155513	Immunotherapy in mismatch repair-deficient metastatic colorectal cancer â€“ Outcome and novel predictive markers	wrong population	round I
rayyan-661155514	Multicenter Evaluation of the Idylla NRAS-BRAF Mutation Test in Metastatic Colorectal Cancer	wrong population	round I
rayyan-661155517	Pseudoprogression in patients treated with immune checkpoint inhibitors for microsatellite instability-high/mismatch repair-deficient metastatic colorectal cancer	wrong population	round I
rayyan-661155523	Phase II Trial of Trametinib and Panitumumab in RAS/RAF Wild Type Metastatic Colorectal Cancer	wrong population	round I
rayyan-661155526	BRAF gene as a potential target to attenuate drug resistance and treat cancer	wrong population	round I
rayyan-661155527	Genomic Sequencing and Insight into Clinical Heterogeneity and Prognostic Pathway Genes in Patients with Metastatic ColorectalÂ Cancer	wrong population	round I
rayyan-661155529	BRAF codons 594 and 596 mutations identify a new molecular subtype of metastatic colorectal cancer at favorable prognosis	wrong population	round I
rayyan-661155530	Intratumor morphologic and transcriptomic heterogeneity in V600EBRAF-mutated metastatic colorectal adenocarcinomas	wrong population	round I
rayyan-661155531	Primary tumour side as a driver for treatment choice in RAS wild-type metastatic colorectal cancer patients: a systematic review and pooled analysis of randomised trials	wrong population	round I
rayyan-661155532	European expert panel consensus on the clinical management of BRAFV600E-mutant metastatic colorectal cancer	wrong population	round I
rayyan-661155535	"Sintilimab plus bevacizumab	wrong population	round I
rayyan-661155536	Application of histology-agnostic treatments in metastatic colorectal cancer	wrong population	round I
rayyan-661155538	"Tucatinib plus trastuzumab for chemotherapy-refractory	wrong population	round I
rayyan-661155539	Lack of Benefit From Anti-EGFR Treatment in RAS and BRAF Wild-type Metastatic Colorectal Cancer With Mucinous Histology or Mucinous Component	wrong population	round I
rayyan-661155540	"Cliniciansâ€™ Attitude to Doublet Plus Anti-EGFR Versus Triplet Plus Bevacizumab as First-line Treatment in Left-Sided RAS and BRAF Wild-Type Metastatic Colorectal Cancer Patients: A Multicenter	wrong population	round I
rayyan-661155542	Phase II study of trifluridine/tipiracil plus bevacizumab by RAS mutation status in patients with metastatic colorectal cancer refractory to standard therapies: JFMC51-1702-C7	wrong population	round I
rayyan-661155543	"Regorafenib plus nivolumab in patients with mismatch repair-proficient/microsatellite stable metastatic colorectal cancer: a single-arm	wrong population	round I

rayyan-661155546	Tumor Location Is Associated With the Prevalence of Braf And Pik3ca Mutations in Patients with Wild-Type Ras Colorectal Cancer: A Prospective Multi-Center Cohort Study in Japan	wrong population	round I
rayyan-661155549	Association of PD-L1 and PD-L2 expression and tumor-infiltrating lymphocytes in BRAF V600E-mutated metastatic colorectal cancer: GI-SCREEN post-hoc analysis	wrong population	round I
rayyan-661155551	"CXD101 and nivolumab in patients with metastatic microsatellite-stable colorectal cancer (CAROSELL): a multicentre	wrong population	round I
rayyan-661155552	MAP2K1 Mutations in Advanced Colorectal Cancer Predict Poor Response to Anti-EGFR Therapy and to Vertical Targeting of MAPK Pathway	wrong population	round I
rayyan-661155553	Monitoring tumour resistance to the BRAF inhibitor combination regimen in colorectal cancer patients via circulating tumour DNA	wrong population	round I
rayyan-661155555	Bevacizumab mitigates codon-specific effects of trifluridine/tipiracil on efficacy outcome parameters in metastatic colorectal cancer	wrong population	round I
rayyan-661155556	Benefit from anti-EGFRs in RAS and BRAF wild-type metastatic transverse colon cancer: a clinical and molecular proof of concept study	wrong population	round I
rayyan-661155557	"Fruquintinib versus placebo in patients with refractory metastatic colorectal cancer (FRESCO-2): an international	wrong population	round I
rayyan-661155560	"KRAS	wrong population	round I
rayyan-661155562	The mechanism investigation of mutation genes in liver and lung metastasis of colorectal cancer by using NGS technique	wrong population	round I
rayyan-661155564	Metastatic pattern is a prognostic factor in BRAFV600E mutant colorectal cancer	wrong population	round I
rayyan-661155567	Current challenges in the implementation of precision oncology for the management of metastatic colorectal cancer	wrong population	round I
rayyan-661155568	"Tumor profiling of KRAS	wrong population	round I
rayyan-661155570	Avelumab versus standard second line treatment chemotherapy in metastatic colorectal cancer patients with microsatellite instability: The SAMCO-PRODIGE 54 randomised phase II trial	wrong population	round I
rayyan-661155571	Treatment and Survival Outcome of BRAF-Mutated Metastatic Colorectal Cancer: A Retrospective Matched Case-Control Study	wrong population	round I
rayyan-661155573	The Pan-Immune-Inflammation Value in microsatellite instabilityâ€“high metastatic colorectal cancer patients treated with immune checkpoint inhibitors	wrong population	round I
rayyan-661155574	High Concordance and Negative Prognostic Impact of RAS/BRAF/PIK3CA Mutations in Multiple Resected Colorectal Liver Metastases	wrong population	round I
rayyan-661155577	RAS and BRAF in the foreground for non-small cell lung cancer and colorectal cancer: Similarities and main differences for prognosis and therapies	wrong population	round I
rayyan-661155578	"Metastatic colorectal carcinoma with signet-ring cells: Clinical	wrong population	round I
rayyan-661155582	Immunopeptidomic Analyses of Colorectal Cancers With and Without Microsatellite Instability	wrong population	round I
rayyan-661155584	"Tumour mutational burden	wrong population	round I
rayyan-661155585	Clinical and molecular characterisation of hereditary and sporadic metastatic colorectal cancers harbouring microsatellite instability/DNA mismatch repair deficiency	wrong population	round I
rayyan-661155586	TP53 Gain-of-Function Mutation is a Poor Prognostic Factor in High-Methylated Metastatic Colorectal Cancer	wrong population	round I

rayyan-661155587	Prognosis and chemosensitivity of non-colorectal alimentary tract cancers with microsatellite instability	wrong population	round I
rayyan-661155588	Latest evidence on immune checkpoint inhibitors in metastatic colorectal cancer: A 2022 update	wrong population	round I
rayyan-661155590	Microsatellite instability is associated with worse overall survival in resectable colorectal liver metastases	wrong population	round I
rayyan-661155591	Metabolic interventions to enhance immunotherapy and targeted therapy efficacy in advanced colorectal cancer	wrong population	round I
rayyan-661155592	Negative hyperselection of elderly patients with RAS and BRAF wild-type metastatic colorectal cancer receiving initial panitumumab plus FOLFOX or 5-FU/LV	wrong population	round I
rayyan-661155798	"KRAS	wrong population	round I
rayyan-661155800	RNF43 mutations predict response to anti-BRAF/EGFR combinatory therapies in BRAF(V600E) metastatic colorectal cancer.	wrong population	round I
rayyan-661155802	Fusobacterium nucleatum in colorectal carcinoma tissue and patient prognosis.	wrong population	round I
rayyan-661155803	BRAF V600E/RAS Mutations and Lynch Syndrome in Patients With MSI-H/dMMR Metastatic Colorectal Cancer Treated With Immune Checkpoint Inhibitors.	wrong population	round I
rayyan-661155806	Mutation status and surgical selection.	wrong population	round I
rayyan-661155817	Recent advances in understanding colorectal cancer.	wrong population	round I
rayyan-661155822	Genomic and prognostic heterogeneity among RAS/BRAF(V600E) /TP53 co-mutated resectable colorectal liver metastases.	wrong population	round I
rayyan-661155823	Colorectal cancer: genetics of development and metastasis.	wrong population	round I
rayyan-661155832	Colorectal cancers with a residual adenoma component: Clinicopathologic features and KRAS mutation.	wrong population	round I
rayyan-661155834	"Chromosomal instability in BRAF mutant	wrong population	round I
rayyan-661155835	Colorectal Cancer with BRAF D594G Mutation Is Not Associated with Microsatellite Instability or Poor Prognosis.	wrong population	round I
rayyan-661155840	Poor prognosis of KRAS or BRAF mutant colorectal liver metastasis without microsatellite instability.	wrong population	round I
rayyan-661155843	"Neuroendocrine Differentiation	wrong population	round I
rayyan-661155845	Incorporating traditional and emerging biomarkers in the clinical management of metastatic colorectal cancer: an update.	wrong population	round I
rayyan-661155852	Distinctive patterns of p53 protein expression and microsatellite instability in human colorectal cancer.	wrong population	round I
rayyan-661155857	Understanding the clinical behavior of relapsed colon cancers with microsatellite instability relative to BRAF mutations.	wrong population	round I
rayyan-661155858	Frequent mutations of KRAS in addition to BRAF in colorectal serrated adenocarcinoma.	wrong population	round I
rayyan-661155865	Molecular characteristics of colorectal hyperplastic polyp subgroups.	wrong population	round I
rayyan-661155867	KRAS and BRAF mutations and MSI status in precursor lesions of colorectal cancer detected by colonoscopy.	wrong population	round I
rayyan-661155872	Combined BRAF and MEK Inhibition With Dabrafenib and Trametinib in BRAF V600-Mutant Colorectal Cancer.	wrong population	round I

rayyan-661155874	"Microsatellite Instability and KRAS Mutation in Stage IV Colorectal Cancer: Prevalence	wrong population	round I
rayyan-661155877	Colorectal mixed adenoneuroendocrine carcinomas and neuroendocrine carcinomas are genetically closely related to colorectal adenocarcinomas.	wrong population	round I
rayyan-661155879	Microsatellite instability and survival in rectal cancer.	wrong population	round I
rayyan-661155881	"p53 mutation is common in microsatellite stable	wrong population	round I
rayyan-661155889	Epithelial-mesenchymal transition and somatic alteration in colorectal cancer with and without peritoneal carcinomatosis.	wrong population	round I
rayyan-661155890	The Evolving Treatment Landscape in BRAF-V600E-Mutated Metastatic Colorectal Cancer.	wrong population	round I
rayyan-661155891	Histopathological Growth Patterns and Survival After Resection of Colorectal Liver Metastasis: An External Validation Study.	wrong population	round I
rayyan-661155906	Clinical and Molecular Features of Post-Colonoscopy Colorectal Cancers.	wrong population	round I
rayyan-661155909	BRAF Mutations Are Associated with Poor Survival Outcomes in Advanced-stage Mismatch Repair-deficient/Microsatellite High Colorectal Cancer.	wrong population	round I
rayyan-661155910	Genetic Characteristics of Resectable Colorectal Cancer with Pulmonary Metastasis.	wrong population	round I
rayyan-661155911	Association of BMI and major molecular pathological markers of colorectal cancer in men and women.	wrong population	round I
rayyan-661155917	Marine Ï‰-3 polyunsaturated fatty acids and risk of colorectal cancer according to microsatellite instability.	wrong population	round I
rayyan-661155918	"[Landscape of KRAS	wrong population	round I
rayyan-661155920	Amplicon-based NGS test for assessing MLH1 promoter methylation and its correlation with BRAF mutation in colorectal cancer patients.	wrong population	round I
rayyan-661155929	Improved Detection of Microsatellite Instability in Early Colorectal Lesions.	wrong population	round I
rayyan-661155937	"BRAF	wrong population	round I
rayyan-661155939	"Microsatellite instability	wrong population	round I
rayyan-661155940	Consequences of a high incidence of microsatellite instability and BRAF-mutated tumors: A population-based cohort of metastatic colorectal cancer patients.	wrong population	round I
rayyan-661155948	Impact of Single-organ Metastasis to the Liver or Lung and Genetic Mutation Status on Prognosis in Stage IV Colorectal Cancer.	wrong population	round I
rayyan-661155949	Concurrent BRAFV600E and BRCA Mutations in MSS Metastatic Colorectal Cancer: Prevalence and Case Series of mCRC patients with prolonged OS.	wrong population	round I
rayyan-661155950	Impact of BRAF mutation and microsatellite instability on the pattern of metastatic spread and prognosis in metastatic colorectal cancer.	wrong population	round I
rayyan-661155951	The Evolving Role of Consensus Molecular Subtypes: a Step Beyond Inpatient Selection for Treatment of Colorectal Cancer.	wrong population	round I
rayyan-661155952	Epigenetics could explain some Moroccan population colorectal cancers peculiarities: microsatellite instability pathway exploration.	wrong population	round I
rayyan-661155953	Rational testing for gene fusion in colorectal cancer: MSI and RAS-BRAF wild-type metastatic colorectal cancer as target population for systematic screening.	wrong population	round I
rayyan-661155955	Multicenter retrospective analysis of metastatic colorectal cancer (CRC) with high-level microsatellite instability (MSI-H).	wrong population	round I

rayyan-661155957	KRAS mutation is associated with lung metastasis in patients with curatively resected colorectal cancer.	wrong population	round I
rayyan-661155967	Frequent PIK3CA Mutations in Colorectal and Endometrial Tumors With 2 or More Somatic Mutations in Mismatch Repair Genes.	wrong population	round I
rayyan-661155971	Promoter methylation of specific genes is associated with the phenotype and progression of colorectal adenocarcinomas.	wrong population	round I
rayyan-661155973	KRAS mutations and CDKN2A promoter methylation show an interactive adverse effect on survival and predict recurrence of rectal cancer.	wrong population	round I
rayyan-661155975	Prognostic implication of CD274 (PD-L1) protein expression in tumor-infiltrating immune cells for microsatellite unstable and stable colorectal cancer.	wrong population	round I
rayyan-661155984	Expression Profile of LGR5 and Its Prognostic Significance in Colorectal Cancer Progression.	wrong population	round I
rayyan-661155992	KRAS mutations in colorectal cancer from Tunisia: relationships with clinicopathologic variables and data on TP53 mutations and microsatellite instability.	wrong population	round I
rayyan-661155997	Chr20q Amplification Defines a Distinct Molecular Subtype of Microsatellite Stable Colorectal Cancer.	wrong population	round I
rayyan-661155998	"Specific mutations in KRAS codons 12 and 13	wrong population	round I
rayyan-661156005	Genetic heterogeneity in synchronous colorectal cancers impacts genotyping approaches and therapeutic strategies.	wrong population	round I
rayyan-661156007	Mismatch Repair (MMR) Gene Alteration and BRAF V600E Mutation Are Potential Predictive Biomarkers of Immune Checkpoint Inhibitors in MMR-Deficient Colorectal Cancer.	wrong population	round I
rayyan-661156011	N_LyST: a simple and rapid screening test for Lynch syndrome.	wrong population	round I
rayyan-661156013	DNA content analysis of colorectal cancer defines a distinct 'microsatellite and chromosome stable' group but does not predict response to radiotherapy.	wrong population	round I
rayyan-661156014	"[Clinicopathological characteristics	wrong population	round I
rayyan-661156015	BRAF provides proliferation and survival signals in MSI colorectal carcinoma cells displaying BRAF(V600E) but not KRAS mutations.	wrong population	round I
rayyan-661156017	Association of Aspirin and Nonsteroidal Anti-Inflammatory Drugs With Colorectal Cancer Risk by Molecular Subtypes.	wrong population	round I
rayyan-661156018	A Prospective Study of Smoking and Risk of Synchronous Colorectal Cancers.	wrong population	round I
rayyan-661156020	Traditional serrated adenoma has two distinct genetic pathways for molecular tumorigenesis with potential neoplastic progression.	wrong population	round I
rayyan-661156022	Molecular heterogeneity and prognostic implications of synchronous advanced colorectal neoplasia.	wrong population	round I
rayyan-661156023	Longitudinal molecular characterization of endoscopic specimens from colorectal lesions.	wrong population	round I
rayyan-661156028	Similarities of molecular genetic changes in synchronous and metachronous colorectal cancers are limited and related to the cancers' proximities to each other.	wrong population	round I
rayyan-661156031	Vitamin D receptor expression is associated with PIK3CA and KRAS mutations in colorectal cancer.	wrong population	round I
rayyan-661156032	Microsatellite stable colorectal cancers stratified by the BRAF V600E mutation show distinct patterns of chromosomal instability.	wrong population	round I
rayyan-661156033	"Colorectal serrated adenocarcinoma shows a different profile of oncogene mutations	wrong population	round I

rayyan-661156034	Serrated polyposis associated with a family history of colorectal cancer and/or polyps: The preferential location of polyps in the colon and rectum defines two molecular entities.	wrong population	round I
rayyan-661156035	Clinicopathologic features of synchronous colorectal carcinoma: A distinct subset arising from multiple sessile serrated adenomas and associated with high levels of microsatellite instability and favorable prognosis.	wrong population	round I
rayyan-661156036	Somatic deletions of the polyA tract in the 3' untranslated region of epidermal growth factor receptor are common in microsatellite instability-high endometrial and colorectal carcinomas.	wrong population	round I
rayyan-661156037	"Comprehensive analysis of CpG island methylator phenotype (CIMP)-high	wrong population	round I
rayyan-661156038	"Dietary intake of fiber	wrong population	round I
rayyan-661156039	Molecular markers and pathway analysis of colorectal carcinoma in the Middle East.	wrong population	round I
rayyan-661156041	Characterisation of PD-L1-positive subsets of microsatellite-unstable colorectal cancers.	wrong population	round I
rayyan-661156043	"Adenoma-like adenocarcinoma: a subtype of colorectal carcinoma with good prognosis	wrong population	round I
rayyan-661156048	A longitudinal study of prediagnostic metabolic biomarkers and the risk of molecular subtypes of colorectal cancer.	wrong population	round I
rayyan-661156049	Tumor LINE-1 methylation level and colorectal cancer location in relation to patient survival.	wrong population	round I
rayyan-661156052	"Comparison of microsatellite instability	wrong population	round I
rayyan-661156054	Population-based screening for Lynch syndrome in Western Australia.	wrong population	round I
rayyan-661156056	Age at onset should be a major criterion for subclassification of colorectal cancer.	wrong population	round I
rayyan-661156057	A very low incidence of BRAF mutations in Middle Eastern colorectal carcinoma.	wrong population	round I
rayyan-661156060	A prospective study of dietary folate and vitamin B and colon cancer according to microsatellite instability and KRAS mutational status.	wrong population	round I
rayyan-661156061	Biomarker testing and mutation prevalence in metastatic colorectal cancer patients in five European countries using a large oncology database.	wrong population	round I
rayyan-661156063	Excess of proximal microsatellite-stable colorectal cancer in African Americans from a multiethnic study.	wrong population	round I
rayyan-661156064	Conventional colon adenomas harbor various disturbances in microsatellite stability and contain micro-serrated foci with microsatellite instability.	wrong population	round I
rayyan-661156065	Efficient molecular screening of Lynch syndrome by specific 3' promoter methylation of the MLH1 or BRAF mutation in colorectal cancer with high-frequency microsatellite instability.	wrong population	round I
rayyan-661156066	"Microsatellite instability affecting the T17 repeats in intron 8 of HSP110	wrong population	round I
rayyan-661156069	Prognostic significance and molecular associations of 18q loss of heterozygosity: a cohort study of microsatellite stable colorectal cancers.	wrong population	round I
rayyan-661156073	Peritoneal carcinomatosis of colorectal cancer: novel clinical and molecular outcomes.	wrong population	round I
rayyan-661156076	"Clinicopathologic and molecular features of sporadic early-onset colorectal adenocarcinoma: an adenocarcinoma with frequent signet ring cell differentiation	wrong population	round I

rayyan-661156077	Retrospective Analysis of Treatment Pathways in Patients With BRAF(V600E)-mutant Metastatic Colorectal Carcinoma - MORSE(CRC).	wrong population	round I
rayyan-661156083	Methylation in p14(ARF) is frequently observed in colorectal cancer with low-level microsatellite instability.	wrong population	round I
rayyan-661156084	Patterns of K-ras mutation in colorectal carcinomas from Iran and Italy (a Gruppo Oncologico dell'Italia Meridionale study): influence of microsatellite instability status and country of origin.	wrong population	round I
rayyan-661156094	Role of BRAF-V600E in the serrated pathway of colorectal tumourigenesis.	wrong population	round I
rayyan-661156098	Genetic characteristics of mitochondrial DNA was associated with colorectal carcinogenesis and its prognosis.	wrong population	round I
rayyan-661156099	Double somatic mutations in mismatch repair genes are frequent in colorectal cancer after Hodgkin's lymphoma treatment.	wrong population	round I
rayyan-661156100	Postmenopausal hormone therapy and colorectal cancer risk by molecularly defined subtypes among older women.	wrong population	round I
rayyan-661156105	NDRG4 stratifies the prognostic value of body mass index in colorectal cancer.	wrong population	round I
rayyan-661156107	Lynch syndrome-associated colorectal carcinoma: frequent involvement of the left colon and rectum and late-onset presentation supports a universal screening approach.	wrong population	round I
rayyan-661156109	Successful treatment of metastatic colorectal cancer with synchronous BRAF V600E mutation and dMMR with BGB-A317.	wrong population	round I
rayyan-661156110	Loss of Hes1 expression is associated with poor prognosis in colorectal adenocarcinoma.	wrong population	round I
rayyan-661156111	Molecular profiling and genome-wide analysis based on somatic copy number alterations in advanced colorectal cancers.	wrong population	round I
rayyan-661156116	Clinicopathologic and genetic characterization of traditional serrated adenomas of the colon.	wrong population	round I
rayyan-661156118	Statin use and colorectal cancer risk according to molecular subtypes in two large prospective cohort studies.	wrong population	round I
rayyan-661156124	KRAS gene mutation in colorectal cancer is correlated with increased proliferation and spontaneous apoptosis.	wrong population	round I
rayyan-661156127	Unique characteristics of CpG island methylator phenotype (CIMP) in a Chinese population with colorectal cancer.	wrong population	round I
rayyan-661156128	Molecular Subtypes Are Frequently Discordant Between Lesions in Patients With Synchronous Colorectal Cancer: Molecular Analysis of 59 Patients.	wrong population	round I
rayyan-661156129	Molecular alterations in colitis-associated colorectal neoplasia: study from a low prevalence area using magnifying chromo colonoscopy.	wrong population	round I
rayyan-661156130	A correlation of the endoscopic characteristics of colonic laterally spreading tumours with genetic alterations.	wrong population	round I
rayyan-661156131	"Commentary: Lifestyle factors and colorectal cancer microsatellite instability--molecular pathological epidemiology science	wrong population	round I
rayyan-661156137	Clinical outcomes of patients with microsatellite-unstable colorectal carcinomas depend on L1 methylation level.	wrong population	round I
rayyan-661156141	Mutations in the WTX-gene are found in some high-grade microsatellite instable (MSI-H) colorectal cancers.	wrong population	round I
rayyan-661156143	The clinicopathological features and prognosis of tumor MSI in East Asian colorectal cancer patients using NCI panel.	wrong population	round I
rayyan-661156145	Annexin A10 expression in colorectal cancers with emphasis on the serrated neoplasia pathway.	wrong population	round I
rayyan-661156146	Externalization of saw-tooth architecture in small serrated polyps implies the presence of methylation of IGFBP7.	wrong population	round I

rayyan-661156147	Clinicopathological comparison of colorectal and endometrial carcinomas in patients with Lynch-like syndrome versus patients with Lynch syndrome.	wrong population	round I
rayyan-661156151	"Molecular characterization of a large unselected cohort of metastatic colorectal cancers in relation to primary tumor location	wrong population	round I
rayyan-661156152	Clinicopathological and molecular features of colorectal serrated neoplasias with different mucosal crypt patterns.	wrong population	round I
rayyan-661156159	Relationship of CDX2 loss with molecular features and prognosis in colorectal cancer.	wrong population	round I
rayyan-661156165	The prognostic impact of RAS on overall survival following liver resection in early versus late-onset colorectal cancer patients.	wrong population	round I
rayyan-661156166	A novel pit pattern identifies the precursor of colorectal cancer derived from sessile serrated adenoma.	wrong population	round I
rayyan-661156169	Morphological characterization of colorectal cancers in The Cancer Genome Atlas reveals distinct morphology-molecular associations: clinical and biological implications.	wrong population	round I
rayyan-661156173	A serrated colorectal cancer pathway predominates over the classic WNT pathway in patients with hyperplastic polyposis syndrome.	wrong population	round I
rayyan-661156182	HER2 status in patients with residual rectal cancer after preoperative chemoradiotherapy: the relationship with molecular results and clinicopathologic features.	wrong population	round I
rayyan-661156183	A prospective study of duration of smoking cessation and colorectal cancer risk by epigenetics-related tumor classification.	wrong population	round I
rayyan-661156188	Carcinoma of the colon and rectum with deregulation of insulin-like growth factor 2 signaling: clinical and molecular implications.	wrong population	round I
rayyan-661156189	Evidence for possible non-canonical pathway(s) driven early-onset colorectal cancer in India.	wrong population	round I
rayyan-661156190	Mutant allele-specific imbalance modulates prognostic impact of KRAS mutations in colorectal adenocarcinoma and is associated with worse overall survival.	wrong population	round I
rayyan-661156193	Assessment of Tumor Sequencing as a Replacement for Lynch Syndrome Screening and Current Molecular Tests for Patients With Colorectal Cancer.	wrong population	round I
rayyan-661156195	"Aberrant promoter methylation of beta-1	wrong population	round I
rayyan-661156196	"Small Bowel Carcinomas in Coeliac or Crohn's Disease: Clinico-pathological	wrong population	round I
rayyan-661156197	Optimizing targeted therapeutic development: analysis of a colorectal cancer patient population with the BRAF(V600E) mutation.	wrong population	round I
rayyan-661156198	Screening for Lynch syndrome and referral to clinical genetics by selective mismatch repair protein immunohistochemistry testing: an audit and cost analysis.	wrong population	round I
rayyan-661156199	Increased cancer predisposition in family members of colorectal cancer patients harboring the p.V600E BRAF mutation: a population-based study.	wrong population	round I
rayyan-661156203	Strategies to identify the Lynch syndrome among patients with colorectal cancer: a cost-effectiveness analysis.	wrong population	round I
rayyan-661156206	Influence of pre-diagnostic cigarette smoking on colorectal cancer survival: overall and by tumour molecular phenotype.	wrong population	round I
rayyan-661156210	Insulin-like growth factor 2 messenger RNA binding protein 3 (IGF2BP3) is a marker of unfavourable prognosis in colorectal cancer.	wrong population	round I
rayyan-661156211	Right-sided rhabdoid colorectal tumors might be related to the serrated pathway.	wrong population	round I
rayyan-661156212	Molecular features of colorectal hyperplastic polyps and sessile serrated adenoma/polyps from Korea.	wrong population	round I

rayyan-661156216	Molecular Markers Beyond Microsatellite Instability for Assessing Prognosis in Early-Stage Colorectal Cancer: What Happens at Relapse?	wrong population	round I
rayyan-661156217	A genetic progression model of Braf(V600E)-induced intestinal tumorigenesis reveals targets for therapeutic intervention.	wrong population	round I
rayyan-661156219	"Tumor TP53 expression status	wrong population	round I
rayyan-661156221	Measurement of Telomere Length in Colorectal Cancers for Improved Molecular Diagnosis.	wrong population	round I
rayyan-661156222	Methylation of MGMT and ADAMTS14 in normal colon mucosa: biomarkers of a field defect for cancerization preferentially targeting elder African-Americans.	wrong population	round I
rayyan-661156228	"Correlations of dynamic contrast-enhanced magnetic resonance imaging with morphologic	wrong population	round I
rayyan-661156229	The utility of BRAFV600E mutation-specific antibody for colon cancers with microsatellite instability.	wrong population	round I
rayyan-661156232	"CpG island methylation is frequently present in tubulovillous and villous adenomas and correlates with size	wrong population	round I
rayyan-661156233	Case of a Patient With Pancreatic Cancer With Sporadic Microsatellite Instability Associated With a BRAF Fusion Achieving Excellent Response to Immunotherapy.	wrong population	round I
rayyan-661156235	Cimp-Positive Status is More Representative in Multiple Colorectal Cancers than in Unique Primary Colorectal Cancers.	wrong population	round I
rayyan-661156236	Down-regulation of epidermal growth factor receptor by selective expansion of a 5'-end regulatory dinucleotide repeat in colon cancer with microsatellite instability.	wrong population	round I
rayyan-661156238	APC mutations and other genetic and epigenetic changes in colon cancer.	wrong population	round I
rayyan-661156239	BRAF: a driver of the serrated pathway in colon cancer.	wrong population	round I
rayyan-661156240	Molecular profiling of signet ring cell colorectal cancer provides a strong rationale for genomic targeted and immune checkpoint inhibitor therapies.	wrong population	round I
rayyan-661156242	The Current Molecular Treatment Landscape of Advanced Colorectal Cancer and Need for the COLOMATE Platform.	wrong population	round I
rayyan-661156243	Decreased Tumoral Expression of Colon-Specific Water Channel Aquaporin 8 Is Associated With Reduced Overall Survival in Colon Adenocarcinoma.	wrong population	round I
rayyan-661156244	A high degree of LINE-1 hypomethylation is a unique feature of early-onset colorectal cancer.	wrong population	round I
rayyan-661156248	BRAF V600E mutation analysis simplifies the testing algorithm for Lynch syndrome.	wrong population	round I
rayyan-661156249	Occurrence of Aurora A positive multipolar mitoses in distinct molecular classes of colorectal carcinomas and effect of Aurora A inhibition.	wrong population	round I
rayyan-661156251	A novel case of rhabdoid colon carcinoma associated with a positive CpG island methylator phenotype and BRAF mutation.	wrong population	round I
rayyan-661156252	Divergent oncogenic changes influence survival differences between colon and rectal adenocarcinomas.	wrong population	round I
rayyan-661156253	Optimizing Adjuvant Therapy for Localized Colon Cancer and Treatment Selection in Advanced Colorectal Cancer.	wrong population	round I
rayyan-661156255	IGF2 differentially methylated region hypomethylation in relation to pathological and molecular features of serrated lesions.	wrong population	round I
rayyan-661156259	"CDK8 expression in 470 colorectal cancers in relation to beta-catenin activation	wrong population	round I
rayyan-661156263	The serrated polyp comes of age.	wrong population	round I

rayyan-661156264	Homozygous PMS2 deletion causes a severe colorectal cancer and multiple adenoma phenotype without extraintestinal cancer.	wrong population	round I
rayyan-661156266	CpG Island Methylator Phenotype May Predict Poor Overall Survival of Patients with Stage IV Colorectal Cancer.	wrong population	round I
rayyan-661156267	Metastatic Colorectal Cancer in Young Adults: A Study From the South Australian Population-Based Registry.	wrong population	round I
rayyan-661156272	CD8+ lymphocytes/ tumour-budding index: an independent prognostic factor representing a 'pro-/anti-tumour' approach to tumour host interaction in colorectal cancer.	wrong population	round I
rayyan-661156278	A modified Lynch syndrome screening algorithm in colon cancer: BRAF immunohistochemistry is efficacious and cost beneficial.	wrong population	round I
rayyan-661156279	Incorporation of somatic BRAF mutation testing into an algorithm for the investigation of hereditary non-polyposis colorectal cancer.	wrong population	round I
rayyan-661156282	Genetic and molecular diversity of colon cancer hepatic metastases.	wrong population	round I
rayyan-661156284	SOX2 expression is associated with a cancer stem cell state and down-regulation of CDX2 in colorectal cancer.	wrong population	round I
rayyan-661156286	BRAF mutation is associated with the CpG island methylator phenotype in colorectal cancer from young patients.	wrong population	round I
rayyan-661156287	Identification of patients with (atypical) MUTYH-associated polyposis by KRAS2 c.34G > T prescreening followed by MUTYH hotspot analysis in formalin-fixed paraffin-embedded tissue.	wrong population	round I
rayyan-661156288	Methylation tolerance due to an O6-methylguanine DNA methyltransferase (MGMT) field defect in the colonic mucosa: an initiating step in the development of mismatch repair-deficient colorectal cancers.	wrong population	round I
rayyan-661156290	Performance of clinical guidelines compared with molecular tumour screening methods in identifying possible Lynch syndrome among colorectal cancer patients: a Norwegian population-based study.	wrong population	round I
rayyan-661156291	The non-crosslinking fixative RCL2Â®-CS100 is compatible with both pathology diagnosis and molecular analyses.	wrong population	round I
rayyan-661156298	Mutation-specific antibody detects mutant BRAFV600E protein expression in human colon carcinomas.	wrong population	round I
rayyan-661156300	Functional TP53 mutations have no impact on response to cytotoxic agents in metastatic colon cancer.	wrong population	round I
rayyan-661156301	Molecular Profiling of Appendiceal Adenocarcinoma and Comparison with Right-sided and Left-sided Colorectal Cancer.	wrong population	round I
rayyan-661156302	Distinct Histopathologic and Molecular Alterations in Inflammatory Bowel Disease-Associated Intestinal Adenocarcinoma: c-MYC Amplification is Common and Associated with Mucinous/Signet Ring Cell Differentiation.	wrong population	round I
rayyan-661156303	"The correlation between immune subtypes and consensus molecular subtypes in colorectal cancer identifies novel tumour microenvironment profiles	wrong population	round I
rayyan-661156305	Sporadic microsatellite instability-high colon cancers rarely display immunohistochemical evidence of Wnt signaling activation.	wrong population	round I
rayyan-661156312	The MLH1 -93 G>A promoter polymorphism and genetic and epigenetic alterations in colon cancer.	wrong population	round I
rayyan-661156314	Colon Cancer Tumor Location Defined by Gene Expression May Disagree With Anatomic Tumor Location.	wrong population	round I
rayyan-661156316	[Prognostic biomarkers for metastatic colorectal cancer].	wrong population	round I
rayyan-661156317	Comprehensive mutation analysis in colorectal flat adenomas.	wrong population	round I

rayyan-661156324	Prospective evaluation of molecular screening for Lynch syndrome in patients with endometrial cancer â‰¤ 70 years.	wrong population	round I
rayyan-661156325	The complex intratumoral heterogeneity of colon cancer highlighted by laser microdissection.	wrong population	round I
rayyan-661156326	Targeted gene sequencing of Lynch syndrome-related and sporadic endometrial carcinomas.	wrong population	round I
rayyan-661156327	"Simplified identification of Lynch syndrome: a prospective	wrong population	round I
rayyan-661156328	MLH1 promoter hypermethylation in the analytical algorithm of Lynch syndrome: a cost-effectiveness study.	wrong population	round I
rayyan-661156330	[Hereditary polyposis: update on molecular genetics and clinicopathologic features].	wrong population	round I
rayyan-661156331	Germline mismatch repair gene variants analyzed by universal sequencing in Japanese cancer patients.	wrong population	round I
rayyan-661156332	"Loss of ARID1A expression and its relationship with PI3K-Akt pathway alterations	wrong population	round I
rayyan-661156333	A multifactorial likelihood model for MMR gene variant classification incorporating probabilities based on sequence bioinformatics and tumor characteristics: a report from the Colon Cancer Family Registry.	wrong population	round I
rayyan-661154048	Glossary	wrong pubblication type	round I
rayyan-661154061	7 - Special Studies	wrong pubblication type	round I
rayyan-661154063	47 - Cholangiocarcinoma	wrong pubblication type	round I
rayyan-661154064	Kapitel 5 - Viszeralchirurgie	wrong pubblication type	round I
rayyan-661154065	Index	wrong pubblication type	round I
rayyan-661154066	Chapter 12 - Therapies II: Specific Non-surgical Treatments	wrong pubblication type	round I
rayyan-661154067	Chapter 11 - Clinical Trial Design in Immuno-Oncology	wrong pubblication type	round I
rayyan-661154068	Subject Index	wrong pubblication type	round I
rayyan-661154070	Molecular techniques in anatomic pathology: An overview	wrong pubblication type	round I
rayyan-661154074	Some thoughts on what's ahead for GI in 2006 and beyond	wrong pubblication type	round I
rayyan-661154076	CHAPTER 42 - Tumour markers	wrong pubblication type	round I

rayyan-661154079	The Gastrointestinal Tumor Microenvironment	wrong pubblication type	round I
rayyan-661154080	"Chapter 14 - Biomarkers: Discovery	wrong pubblication type	round I
rayyan-661154082	CHAPTER 23 - Large Intestine (Colon)	wrong pubblication type	round I
rayyan-661154085	A - ERKRANKUNGEN DER VERDAUUNGSORGANE	wrong pubblication type	round I
rayyan-661154087	"Chapter 10 - Typing	wrong pubblication type	round I
rayyan-661154088	Chapter 13 - Tumours and Tumour-Like Lesions	wrong pubblication type	round I
rayyan-661154090	2 - Intracellular Signaling	wrong pubblication type	round I
rayyan-661154092	Chapter 12 - Clinical pharmacogenetics	wrong pubblication type	round I
rayyan-661154093	"XIII National Congress of Digestive Diseases	wrong pubblication type	round I
rayyan-661154094	17 - Tumors of the Brain and Spinal Cord	wrong pubblication type	round I
rayyan-661154096	Chapter Three - A Macro View of MicroRNAs: The Discovery of MicroRNAs and Their Role in Hematopoiesis and Hematologic Disease	wrong pubblication type	round I
rayyan-661154097	Chapter 2 - Nasopharyngeal Carcinoma: Genetics and Genomics	wrong pubblication type	round I
rayyan-661154099	C	wrong pubblication type	round I
rayyan-661154100	Chapter 34 - Novel Therapeutic Approaches and Targets for Ovarian Cancer	wrong pubblication type	round I
rayyan-661154101	Minimizing the risk of small-for-size syndrome after liver surgery	wrong pubblication type	round I
rayyan-661154102	Analysis of Molecular Aberrations in Ovarian Cancer Allows Novel Target Identification	wrong pubblication type	round I
rayyan-661154104	The Future of Precision Medicine in Oncology	wrong pubblication type	round I

rayyan-661154105	"15 - Pathology	wrong pubblication type	round I
rayyan-661154106	Chapter 60 - Pathology of the Lower Gastrointestinal Tract	wrong pubblication type	round I
rayyan-661154107	Chapter 13 - Application of Molecular Diagnostics	wrong pubblication type	round I
rayyan-661154110	Chapter 2 - Immunotherapeutic Approaches in Cancer	wrong pubblication type	round I
rayyan-661154112	Chapter 3 - DNA Methylation Alterations in Human Cancers	wrong pubblication type	round I
rayyan-661154113	Cancer modelling in the NGS era â€“ Part I: Emerging technology and initial modelling	wrong pubblication type	round I
rayyan-661154120	25 - Cancer Pharmacology	wrong pubblication type	round I
rayyan-661154127	Immune checkpoint therapyâ€”current perspectives and future directions	wrong pubblication type	round I
rayyan-661154128	"External Quality Assessment Schemes for Biomarker Testing in Oncology: Comparison of Performance between Formalin-Fixed	wrong pubblication type	round I
rayyan-661154130	Chapter 11 - Prognostic Epigenetics	wrong pubblication type	round I
rayyan-661154135	"Chapter Two - From immune checkpoints to vaccines: The past	wrong pubblication type	round I
rayyan-661154136	Chapter 6 - Which technology performs better? From sample volume to extraction and molecular profiling	wrong pubblication type	round I
rayyan-661154139	Chapter 4 - Tumor immunology	wrong pubblication type	round I
rayyan-661154147	Toward a More Precise Future for Oncology	wrong pubblication type	round I
rayyan-661154154	Chapter Eight - Patient-derived organoids as a model for tumor research	wrong pubblication type	round I
rayyan-661154155	Towards interpretable imaging genomics analysis: Methodological developments and applications	wrong pubblication type	round I
rayyan-661154158	"Molecular pathological epidemiology of epigenetics: emerging integrative science to analyze environment	wrong pubblication type	round I

rayyan-661154159	CHAPTER 34 - New techniques	wrong pubblication type	round I
rayyan-661154264	Diagnostic and prognostic impact of cell-free DNA in human cancers: Systematic review	wrong pubblication type	round I
rayyan-661154265	Early detection of lung cancer biomarkers through biosensor technology: A review	wrong pubblication type	round I
rayyan-661154269	Molecular Profiling of Cancerâ€"The Future of Personalized Cancer Medicine: A Primer on Cancer Biology and the Tools Necessary to Bring Molecular Testing to the Clinic	wrong pubblication type	round I
rayyan-661154384	Adjuvant colon cancer chemotherapy: where we are and where we'll go	wrong pubblication type	round I
rayyan-661154386	Precision Oncology: Current Scenario and Future Promises and Challenges	wrong pubblication type	round I
rayyan-661154388	Longitudinal ctDNA profiling in precision oncology and immunÎ¿-oncology	wrong pubblication type	round I
rayyan-661154399	Nanotechnologies for the treatment of colon cancer: From old drugs to new hope	wrong pubblication type	round I
rayyan-661154406	Interplay between MAPK/ERK signaling pathway and MicroRNAs: A crucial mechanism regulating cancer cell metabolism and tumor progression	wrong pubblication type	round I
rayyan-661154408	KRAS mutations: Analytical considerations	wrong pubblication type	round I
rayyan-661154413	Complete pathological response in rectal cancer utilising novel treatment strategies for neo-adjuvant therapy: A systematic review	wrong pubblication type	round I
rayyan-661154472	"Adjuvant chemotherapy in resected colon cancer: When	wrong pubblication type	round I
rayyan-661154479	Performance of prediction models on survival outcomes of colorectal cancer with surgical resection: A systematic review and meta-analysis	wrong pubblication type	round I
rayyan-661154492	Dietary phytochemicals in colorectal cancer prevention and treatment: A focus on the molecular mechanisms involved	wrong pubblication type	round I
rayyan-661154503	Integration of genetic variants and gene network for drug repurposing in colorectal cancer	wrong pubblication type	round I
rayyan-661154504	Development of circulating tumour DNA analysis for gastrointestinal cancers	wrong pubblication type	round I
rayyan-661154507	"Deciphering the role of KRAS gene in oncogenesis: Focus on signaling pathways	wrong pubblication type	round I

rayyan-661154508	Epigenetic Alterations in Colorectal Cancer: EmergingÂ Biomarkers	wrong pubblication type	round I
rayyan-661154523	Omics technologies for improved diagnosis and treatment of colorectal cancer: Technical advancement and major perspectives	wrong pubblication type	round I
rayyan-661154524	Hypermutated tumours in the era of immunotherapy: The paradigm of personalised medicine	wrong pubblication type	round I
rayyan-661154526	Targeted Therapies as Adjuvant Treatment for Early-Stage Colorectal Cancer: First Impressions and Clinical Questions	wrong pubblication type	round I
rayyan-661154529	Advances on colorectal cancer 3D models: The needed translational technology for nanomedicine screening	wrong pubblication type	round I
rayyan-661154530	Nanotechnology is an important strategy for combinational innovative chemo-immunotherapies against colorectal cancer	wrong pubblication type	round I
rayyan-661154533	Natural language processing of German clinical colorectal cancer notes for guideline-based treatment evaluation	wrong pubblication type	round I
rayyan-661154537	Epigenetic modifications in colorectal cancer: Molecular insights and therapeutic challenges	wrong pubblication type	round I
rayyan-661154538	The significance of gene mutations across eight major cancer types	wrong pubblication type	round I
rayyan-661154540	Predictive and prognostic biomarkers in colorectal cancer: A systematic review of recent advances and challenges	wrong pubblication type	round I
rayyan-661154541	Advances in adjuvant therapy of colon cancer	wrong pubblication type	round I
rayyan-661154542	"Refining adjuvant therapy for non-metastatic colon cancer	wrong pubblication type	round I
rayyan-661154546	Molecular approaches in cancer	wrong pubblication type	round I
rayyan-661154548	Epigenetics of Colorectal Cancer	wrong pubblication type	round I
rayyan-661154549	Molecular Prognostic Markers in Locally Advanced Colon Cancer	wrong pubblication type	round I
rayyan-661154552	"Colorectal cancer peritoneal metastases: Biology	wrong pubblication type	round I
rayyan-661154553	Prospective Derivation of a Living Organoid Biobank of Colorectal Cancer Patients	wrong pubblication type	round I

rayyan-661154560	The role of protein tyrosine phosphatases in colorectal cancer	wrong pubblication type	round I
rayyan-661154563	"Drug resistance	wrong pubblication type	round I
rayyan-661154564	Oncogenic miRNAs and target therapies in colorectal cancer	wrong pubblication type	round I
rayyan-661154566	The primary tumor location impacts survival outcome of colorectal liver metastases after hepatic resection: A systematic review and meta-analysis	wrong pubblication type	round I
rayyan-661154569	"DNA damage response and repair in colorectal cancer: Defects	wrong pubblication type	round I
rayyan-661154570	"5-fluorouracil and other fluoropyrimidines in colorectal cancer: Past	wrong pubblication type	round I
rayyan-661154578	The seen and the unseen: Molecular classification and image based-analysis of gastrointestinal cancers	wrong pubblication type	round I
rayyan-661154580	Primary tumor location and the prognosis of patients after local treatment of colorectal liver metastases: a systematic review and meta-analysis	wrong pubblication type	round I
rayyan-661154581	Subgroups and prognostication in stage III colon cancer: future perspectives for adjuvant therapy	wrong pubblication type	round I
rayyan-661154583	Unraveling molecular pathways of poorly differentiated neuroendocrine carcinomas of the gastroenteropancreatic system: A systematic review	wrong pubblication type	round I
rayyan-661154601	A meta-analysis of microsatellite instability and colorectal cancer prognosis abstract	wrong pubblication type	round I
rayyan-661154638	"Clinical	wrong pubblication type	round I
rayyan-661154641	Probing the colorectal cancer proteome for biomarkers: Current status and perspectives	wrong pubblication type	round I
rayyan-661154642	A Critical Review of the Role of Local Therapy for Oligometastatic Gastrointestinal Cancer	wrong pubblication type	round I
rayyan-661154643	Treatment strategies and drug resistance mechanisms in adenocarcinoma of different organs	wrong pubblication type	round I
rayyan-661154645	"Molecular subtyping of colorectal cancer: Recent progress	wrong pubblication type	round I
rayyan-661154656	Predictive Markers in Colorectal Cancer	wrong pubblication type	round I

rayyan-661154663	Predictive and prognostic biomarkers in gastrointestinal tract tumours	wrong pubblication type	round I
rayyan-661154664	The molecular biology of colorectal carcinoma and its implications: A review	wrong pubblication type	round I
rayyan-661154665	Microbe-based therapies for colorectal cancer: Advantages and limitations	wrong pubblication type	round I
rayyan-661154667	A comprehensive overview of promising biomarkers in stage II colorectal cancer	wrong pubblication type	round I
rayyan-661154673	Intestinal multicellular organoids to study colorectal cancer	wrong pubblication type	round I
rayyan-661154681	The clinical relevance of gene expression based prognostic signatures in colorectal cancer	wrong pubblication type	round I
rayyan-661154682	"Molecular Biomarkers for the Evaluation of Colorectal Cancer: Guideline From the American Society for Clinical Pathology	wrong pubblication type	round I
rayyan-661154784	The most recent advances in immunotherapy with checkpoint inhibitors in gastrointestinal cancers	wrong pubblication type	round I
rayyan-661154787	Molecular subtypes of colorectal cancer: AnÂ emerging therapeutic opportunity for personalized medicine	wrong pubblication type	round I
rayyan-661154788	Portrait of the PI3K/AKT pathway in colorectal cancer	wrong pubblication type	round I
rayyan-661154789	Rectal and colon cancer: Not just a different anatomic site	wrong pubblication type	round I
rayyan-661154790	Coiled-coil domain-containing 154 promotes colorectal cancer proliferation and metastasis via interacting with minichromosome maintenance complex component 2	wrong pubblication type	round I
rayyan-661154792	Simple classifiers for molecular subtypes of colorectal cancer	wrong pubblication type	round I
rayyan-661154796	Mutation Hotspots in the Î²-Catenin Gene: Lessons from the Human Cancer Genome Databases	wrong pubblication type	round I
rayyan-661154797	Evolving molecular classification by genomic and proteomic biomarkers in colorectal cancer: Potential implications for the surgical oncologist	wrong pubblication type	round I
rayyan-661154799	Targeting the human epidermal growth factor receptor 2 (HER2) oncogene in colorectal cancer	wrong pubblication type	round I
rayyan-661154801	Metabolic pathways regulating colorectal cancer initiation and progression	wrong pubblication type	round I

rayyan-661154802	Polymeric nanoparticles approach and identification and characterization of novel biomarkers for colon cancer	wrong pubblication type	round I
rayyan-661154803	Integrating Biomarkers Into Clinical Decision Making for Colorectal Cancer	wrong pubblication type	round I
rayyan-661154804	"Blood-based biomarkers for diagnosis	wrong pubblication type	round I
rayyan-661154808	Chapter 2 - Translational epigenetics in precision medicine of colorectal cancer	wrong pubblication type	round I
rayyan-661154810	An Update on Colorectal Cancer	wrong pubblication type	round I
rayyan-661154812	Exosomal-long non-coding RNAs journey in colorectal cancer: Evil and goodness faces of key players	wrong pubblication type	round I
rayyan-661154813	Association of DNA repair gene polymorphisms with colorectal cancer risk and treatment outcomes	wrong pubblication type	round I
rayyan-661154814	Peritoneal carcinomatosis from colorectal cancer in the pediatric population: Cytoreductive surgery and HIPEC. A systematic review	wrong pubblication type	round I
rayyan-661154817	B-Raf mutation: A key player in molecular biology of cancer	wrong pubblication type	round I
rayyan-661154818	Therapeutic Targeting of the Colorectal Tumor Stroma	wrong pubblication type	round I
rayyan-661154819	Dendritic cell vaccine therapy for colorectal cancer	wrong pubblication type	round I
rayyan-661154822	Precision Treatment and Prevention of Colorectal Cancerâ€”Hope or Hype?	wrong pubblication type	round I
rayyan-661154823	Advances of immune checkpoints in colorectal cancer treatment	wrong pubblication type	round I
rayyan-661154827	Understanding the role of primary tumour localisation in colorectal cancer treatment and outcomes	wrong pubblication type	round I
rayyan-661154830	K-Ras and its inhibitors towards personalized cancer treatment: Pharmacological and structural perspectives	wrong pubblication type	round I
rayyan-661154832	A perspective on medicinal chemistry approaches towards adenomatous polyposis coli and Wnt signal based colorectal cancer inhibitors	wrong pubblication type	round I
rayyan-661154833	Cancers du cÃ´lon : prise en charge molÃ©culaire	wrong pubblication type	round I

rayyan-661154836	Anti-CEACAM5 ADC M9140 in Advanced Solid Tumors	wrong pubblication type	round I
rayyan-661154856		wrong pubblication type	round I
rayyan-661154979	Comparing international data between keyhole and robotic rectal cancer surgery	wrong pubblication type	round I
rayyan-661154980	Short treatment with the drug cyclophosphamide in bowel cancer	wrong pubblication type	round I
rayyan-661154981	Early screening for colorectal cancer via a simple blood sample	wrong pubblication type	round I
rayyan-661154982	Comparative analysis of the protective effect of different screening strategies for colorectal cancer	wrong pubblication type	round I
rayyan-661154983	Intestinal cancer and the risk for other cancer in the same person	wrong pubblication type	round I
rayyan-661154984	Could supported weight loss reduce bowel cancer surgery complications?	wrong pubblication type	round I
rayyan-661154985	Exercise prehabilitation in colorectal cancer	wrong pubblication type	round I
rayyan-661154986	Understanding Raman signatures in colorectal cancer	wrong pubblication type	round I
rayyan-661154987	Patient acceptability of FIT stool test and analysing a colorectal cancer risk tool	wrong pubblication type	round I
rayyan-661154988	Quality of life after surgery and other options to prevent cancer of the lining of the womb (endometrial cancer)	wrong pubblication type	round I
rayyan-661154989	Injection of IP-001 into thermally ablated solid tumors	wrong pubblication type	round I
rayyan-661154990	Evaluation of an intervention co-designed with key stakeholders to improve colorectal cancer screening participation	wrong pubblication type	round I
rayyan-661154991	Can we analyse stool content to improve the prediction of the risk of developing bowel cancer?	wrong pubblication type	round I
rayyan-661154992	The effects of a colorectal cancer screening programme for average-risk older Chinese adults	wrong pubblication type	round I
rayyan-661154993	A study of functional and immunological outcomes in people undergoing robotic and non-robotic rectal cancer surgery	wrong pubblication type	round I

rayyan-661154994	A feasibility study of electron radiotherapy delivered during a surgical operation for rectal cancer	wrong pubblication type	round I
rayyan-661154995	Feasibility study of lidocaine infusion during bowel cancer surgery for cancer outcome	wrong pubblication type	round I
rayyan-661154996	Development of a chart that can be used to aid prediction of hidden cancerous growths in colorectal cancer patients before surgery	wrong pubblication type	round I
rayyan-661154997	A trial to assess the efficacy of a new drug versus standard care for anaemia following colorectal cancer surgery	wrong pubblication type	round I
rayyan-661154998	A study to determine the feasibility and impact of using a remote (app-based) lifestyle change program to reduce the length of stay and complications after colorectal resection surgery	wrong pubblication type	round I
rayyan-661154999	Determining colorectal cancer features using a Rwandan population to lay a foundation and inform precise colorectal cancer clinical management	wrong pubblication type	round I
rayyan-661155000	Can extra tests on cancer samples identify more patients with bowel/colon cancer who should be treated with drugs called anti-EGFR agents?	wrong pubblication type	round I
rayyan-661155001	Is it possible to use home-based prehabilitation with added E-health in patients with colorectal cancer undergoing surgery?	wrong pubblication type	round I
rayyan-661155002	Testing if the SonoTran Platform can enhance drug delivery in metastatic colorectal cancer	wrong pubblication type	round I
rayyan-661155003	Examining the efficacy of faecal immunochemical testing (FIT) in patients with Lynch Syndrome	wrong pubblication type	round I
rayyan-661155004	Can messages based on behavioral economics increase participation in colorectal cancer screening programmes?	wrong pubblication type	round I
rayyan-661155005	The effect of supplementation with an amino acid that helps the body build protein (L-arginine) on change in the immune system of colorectal cancer patients	wrong pubblication type	round I
rayyan-661155006	A pilot randomised controlled trial of an app with brief behavioural support to promote physical activity after a cancer diagnosis	wrong pubblication type	round I
rayyan-661155007	Clinical study to assess the outcomes of a patient-centred survivorship care plan enhanced with big data and artificial intelligence technologies	wrong pubblication type	round I
rayyan-661155013	Colorectal cancer cohort study (COLO-COHORT)	wrong pubblication type	round I
rayyan-661155035	Colorectal cancer subtypes: Translation to routine clinical pathology	wrong pubblication type	round I
rayyan-661155037	Colorectal cancer: epigenetic alterations and their clinical implications	wrong pubblication type	round I

rayyan-661155039	Opportunities on the horizon for the management of early colon cancer	wrong pubblication type	round I
rayyan-661155042	Chasing the personalized medicine dream through biomarker validation in colorectal cancer	wrong pubblication type	round I
rayyan-661155044	"An update on colorectal cancer microenvironment	wrong pubblication type	round I
rayyan-661155047	Could immunoscore improve the prognostic and therapeutic management in patients with solid tumors?	wrong pubblication type	round I
rayyan-661155049	Nanobody-based therapeutics against colorectal cancer: Precision therapies based on the personal mutanome profile and tumor neoantigens	wrong pubblication type	round I
rayyan-661155051	Prognostic and predictive biomarkers for epidermal growth factor receptor-targeted therapy in colorectal cancer: Beyond KRAS mutations	wrong pubblication type	round I
rayyan-661155053	Angiogenesis inhibition in the second-line treatment of metastatic colorectal cancer: A systematic review and pooled analysis	wrong pubblication type	round I
rayyan-661155058	The predictive value of primary tumor location in patients with metastatic colorectal cancer: A systematic review	wrong pubblication type	round I
rayyan-661155061	HER2: An emerging target in colorectal cancer	wrong pubblication type	round I
rayyan-661155063	Current advances in detecting genetic and epigenetic biomarkers of colorectal cancer	wrong pubblication type	round I
rayyan-661155068	BRAF gene: From human cancers to developmental syndromes	wrong pubblication type	round I
rayyan-661155071	Molecular Testing of Colorectal Cancer in the Modern Era: What Are We Doing and Why?	wrong pubblication type	round I
rayyan-661155079	Drivers of cancer metastasis â€“ Arise early and remain present	wrong pubblication type	round I
rayyan-661155135	eRAPID cognitive interview study	wrong pubblication type	round I
rayyan-661155136	Evaluation of an e-health intervention for cancer patients support	wrong pubblication type	round I
rayyan-661155230	Chemotherapy plus bevacizumab as an optimal first-line therapeutic treatment for patients with right-sided metastatic colon cancer: a meta-analysis of first-line clinical trials	wrong pubblication type	round I
rayyan-661155234	A special subtype: Revealing the potential intervention and great value of KRAS wildtype pancreatic cancer	wrong pubblication type	round I

rayyan-661155235	Predictive Molecular Classifiers in Colorectal Cancer	wrong pubblication type	round I
rayyan-661155242	"Impact of polymorphisms within genes involved in regulating DNA methylation in patients with metastatic colorectal cancer enrolled in three independent	wrong pubblication type	round I
rayyan-661155243	Perspectives for tailored chemoprevention and treatment of colorectal cancer in Lynch syndrome	wrong pubblication type	round I
rayyan-661155244	Impact of tissue-agnostic approvals for patients with gastrointestinal malignancies	wrong pubblication type	round I
rayyan-661155253	Clinical update on K-Ras targeted therapy in gastrointestinal cancers	wrong pubblication type	round I
rayyan-661155254	Comprehensive intra-individual genomic and transcriptional heterogeneity: Evidence-based Colorectal Cancer Precision Medicine	wrong pubblication type	round I
rayyan-661155258	Chapter 16 - Systemic treatment of localized colorectal cancer	wrong pubblication type	round I
rayyan-661155262	Clinical presentation and outcome of patients with enteric-type adenocarcinoma of the lung: A pooled analysis of published cases	wrong pubblication type	round I
rayyan-661155263	A Genomic Analysis Workflow for Colorectal Cancer Precision Oncology	wrong pubblication type	round I
rayyan-661155264	Salvage regional therapy using hepatic artery infusion pump in unresectable chemotherapy resistant colorectal liver metastases	wrong pubblication type	round I
rayyan-661155267	The role of molecular biomarkers in outcomes and patient selection for cytoreductive surgery and hyperthermic intraperitoneal chemotherapy for peritoneal metastases of colorectal origin	wrong pubblication type	round I
rayyan-661155269	Development of Novel Targeted Agents in the Treatment of Metastatic Colorectal Cancer	wrong pubblication type	round I
rayyan-661155270	Development of Molecular Biomarkers in Individualized Treatment of Colorectal Cancer	wrong pubblication type	round I
rayyan-661155273	Personalizing Therapy for Colorectal Cancer	wrong pubblication type	round I
rayyan-661155274	KRAS Mutation Screening in Colorectal Cancer: From Paper to Practice	wrong pubblication type	round I
rayyan-661155275	Comprehensive pancancer genomic analysis reveals (RTK)-RAS-RAF-MEK as a key dysregulated pathway in cancer: Its clinical implications	wrong pubblication type	round I
rayyan-661155280	"Colorectal cancer heterogeneity and targeted therapy: Clinical implications	wrong pubblication type	round I

rayyan-661155282	"Recent progress of targeted nanocarriers in diagnostic	wrong pubblication type	round I
rayyan-661155284	"Elaboration of NTRK-rearranged colorectal cancer: Integration of immunoreactivity pattern	wrong pubblication type	round I
rayyan-661155285	A comprehensive framework for early-onset colorectal cancer research	wrong pubblication type	round I
rayyan-661155289	Clinical Implications of Colorectal Cancer Stem Cells in the Age of Single-Cell Omics and Targeted Therapies	wrong pubblication type	round I
rayyan-661155290	Clinical relevance of colorectal cancer molecular subtypes	wrong pubblication type	round I
rayyan-661155291	Advances in radiological staging of colorectal cancer	wrong pubblication type	round I
rayyan-661155292	The DNA damage response pathway as a land of therapeutic opportunities for colorectal cancer	wrong pubblication type	round I
rayyan-661155298	Biomarkers and Targeted Therapeutics in Colorectal Cancer	wrong pubblication type	round I
rayyan-661155299	Diagnostic and prognostic biomarkers in colorectal cancer and the potential role of exosomes in drug delivery	wrong pubblication type	round I
rayyan-661155300	Molecular Insights Into Colorectal Carcinoma	wrong pubblication type	round I
rayyan-661155301	Significance of Liquid Biopsy for Monitoring and Therapy Decision of Colorectal Cancer	wrong pubblication type	round I
rayyan-661155305	Colorectal cancer immunotherapy-Recent progress and future directions	wrong pubblication type	round I
rayyan-661155307	"Methods and biomarkers for early detection	wrong pubblication type	round I
rayyan-661155308	Immunotherapy efficacy on mismatch repair-deficient colorectal cancer: From bench to bedside	wrong pubblication type	round I
rayyan-661155311	"Integrated approaches for precision oncology in colorectal cancer: The more you know	wrong pubblication type	round I
rayyan-661155312	Predictive and Prognostic Biomarkers for Targeted Therapy in Metastatic Colorectal Cancer	wrong pubblication type	round I
rayyan-661155313	The Changing Demographics of Colorectal Cancer: Rising Incidence in Younger Individuals	wrong pubblication type	round I

rayyan-661155315	Clonal evolution and expansion associated with therapy resistance and relapse of colorectal cancer	wrong pubblication type	round I
rayyan-661155316	"Chapter 10 - Colorectal cancer heterogeneity and targeted therapy: clinical implications	wrong pubblication type	round I
rayyan-661155317	Primary and secondary immune checkpoint inhibitors resistance in colorectal cancer: Key mechanisms and ways to overcome resistance	wrong pubblication type	round I
rayyan-661155319	Prognosis in colorectal cancer beyond TNM	wrong pubblication type	round I
rayyan-661155322	HER2 in Colorectal Carcinoma: Are We There yet?	wrong pubblication type	round I
rayyan-661155323	Performance characteristics of next-generation sequencing in clinical mutation detection of colorectal cancers	wrong pubblication type	round I
rayyan-661155327	Treatments after first progression in metastatic colorectal cancer. A literature review and evidence-based algorithm	wrong pubblication type	round I
rayyan-661155331	Efficacy and safety of HER2-targeted inhibitors for metastatic colorectal cancer with HER2-amplified: A meta-analysis	wrong pubblication type	round I
rayyan-661155333	Colorectal cancer cell exosome and cytoplasmic membrane for homotypic delivery of therapeutic molecules	wrong pubblication type	round I
rayyan-661155335	Tumor sequencing of African ancestry reveals differences in clinically relevant alterations across common cancers	wrong pubblication type	round I
rayyan-661155336	Immunotherapy of colorectal cancer: Challenges for therapeutic efficacy	wrong pubblication type	round I
rayyan-661155338	A practical guide to biomarkers for the evaluation of colorectal cancer	wrong pubblication type	round I
rayyan-661155339	"A review of the sensitivity of metastatic colorectal cancer patients with deficient mismatch repair to standard-of-care chemotherapy and monoclonal antibodies	wrong pubblication type	round I
rayyan-661155340	Recommendations for the use of next-generation sequencing in patients with metastatic cancer in the Asia-Pacific region: a report from the APODDC working group	wrong pubblication type	round I
rayyan-661155342	Polymerase proofreading domain mutations: New opportunities for immunotherapy in hypermutated colorectal cancer beyond MMR deficiency	wrong pubblication type	round I
rayyan-661155343	A synopsis of modern - day colorectal cancer: Where we stand	wrong pubblication type	round I
rayyan-661155344	The diverse molecular profiles of lynch syndrome-associated colorectal cancers are (highly) dependent on underlying germline mismatch repair mutations	wrong pubblication type	round I

rayyan-661155345	HER2 as a Predictive Biomarker and Treatment Target in Colorectal Cancer	wrong pubblication type	round I
rayyan-661155347	Overcoming dynamic molecular heterogeneity in metastatic colorectal cancer: Multikinase inhibition with regorafenib and the case of rechallenge with anti-EGFR	wrong pubblication type	round I
rayyan-661155352	Anti-PD-1 combined with targeted therapy: Theory and practice in gastric and colorectal cancer	wrong pubblication type	round I
rayyan-661155355	Oxaliplatin retreatment in metastatic colorectal cancer: Systematic review and future research opportunities	wrong pubblication type	round I
rayyan-661155359	The relevance of primary tumour location in patients with metastatic colorectal cancer: A meta-analysis of first-line clinical trials	wrong pubblication type	round I
rayyan-661155362	Nano-enabled colorectal cancer therapy	wrong pubblication type	round I
rayyan-661155364	MSIplus for Integrated Colorectal Cancer Molecular Testing by Next-Generation Sequencing	wrong pubblication type	round I
rayyan-661155365	HER2 targeted therapy in colorectal cancer: New horizons	wrong pubblication type	round I
rayyan-661155366	Evaluation of immune infiltrate according to the HER2 status in colorectal cancer	wrong pubblication type	round I
rayyan-661155367	BRAF V600E mutation-specific antibody: A review	wrong pubblication type	round I
rayyan-661155370	Recent Advances in the Clinical Development of Immune Checkpoint Blockade Therapy for Mismatch Repair Proficient (pMMR)/non-MSI-H Metastatic Colorectal Cancer	wrong pubblication type	round I
rayyan-661155372	Small-molecule drugs of colorectal cancer: Current status and future directions	wrong pubblication type	round I
rayyan-661155377	Update on Mucoadhesive Approaches to target Drug Delivery in Colorectal Cancer	wrong pubblication type	round I
rayyan-661155383	RET fusions in a small subset of advanced colorectal cancers at risk of being neglected	wrong pubblication type	round I
rayyan-661155384	Immune scores in colorectal cancer: Where are we?	wrong pubblication type	round I
rayyan-661155386	Short interfering RNA in colorectal cancer: is it wise to shoot the messenger?	wrong pubblication type	round I
rayyan-661155392	Biomarker concordance between primary colorectal cancer and its metastases	wrong pubblication type	round I

rayyan-661155394	The prognostic potential of CDX2 in colorectal cancer: Harmonizing biology and clinical practice	wrong pubblication type	round I
rayyan-661155395	Translational research of new developments in targeted therapy of colorectal cancer	wrong pubblication type	round I
rayyan-661155399	Clinicopathological relevance of BRAF mutations in human cancer	wrong pubblication type	round I
rayyan-661155400	The updates on metastatic mechanism and treatment of colorectal cancer	wrong pubblication type	round I
rayyan-661155401	Clinical and Molecular Features in Patients Undergoing Cytoreductive Surgery and Hyperthermic Intraperitoneal Chemotherapy for Peritoneal Carcinosis from Colorectal Cancer	wrong pubblication type	round I
rayyan-661155402	The best strategy for metastatic colorectal cancer (mCRC) patients in second-line treatment: A network meta-analysis	wrong pubblication type	round I
rayyan-661155404	Resistance to anti-epidermal growth factor receptor in metastatic colorectal cancer: What does still need to be addressed?	wrong pubblication type	round I
rayyan-661155408	Liquid biopsy in colorectal cancer: Onward and upward	wrong pubblication type	round I
rayyan-661155412	Treatment sequencing of metastatic colorectal cancer based on primary tumor location	wrong pubblication type	round I
rayyan-661155416	Prognostic and predictive molecular biomarkers in advanced colorectal cancer	wrong pubblication type	round I
rayyan-661155418	"Colorectal cancer: Genetic alterations	wrong pubblication type	round I
rayyan-661155423	Strategic enhancement of immune checkpoint inhibition in refractory Colorectal Cancer: Trends and future prospective	wrong pubblication type	round I
rayyan-661155436	The impact of microsatellite stability status in colorectal cancer	wrong pubblication type	round I
rayyan-661155463	Immunotherapy in colorectal cancer: is the long-awaited revolution finally happening?	wrong pubblication type	round I
rayyan-661155465	Third- or Later-line Therapy for Metastatic Colorectal Cancer: Reviewing Best Practice	wrong pubblication type	round I
rayyan-661155477	Systematic review of tumour budding and association with common mutations in patients with colorectal cancer	wrong pubblication type	round I
rayyan-661155479	23 - Colorectal cancer	wrong pubblication type	round I

rayyan-661155480	"BRAF mutant colorectal cancer: prognosis	wrong pubblication type	round I
rayyan-661155483	Current progress of immune checkpoint inhibitors for advanced colorectal cancer: concentrating on the efficacy improvement	wrong pubblication type	round I
rayyan-661155487	Prognostic and predictive markers in liver limited stage IV colorectal cancer	wrong pubblication type	round I
rayyan-661155502	Metastatic colorectal cancer: mechanisms and emerging therapeutics	wrong pubblication type	round I
rayyan-661155509	An empirical review on the resistance mechanisms of epidermal growth factor receptor inhibitors and predictive molecular biomarkers in colorectal cancer	wrong pubblication type	round I
rayyan-661155518	Cancers colorectaux avec mutation V600E de BRAFÂ : oÃ¹ en sommes-nousÂ ?	wrong pubblication type	round I
rayyan-661155520	"Encorafenib	wrong pubblication type	round I
rayyan-661155522	SET nuclear proto-oncogene gene expression is associated with microsatellite instability in human colorectal cancer identified by co-expression analysis	wrong pubblication type	round I
rayyan-661155525	Colorectal Cancer Modeling with Organoids: Discriminating between Oncogenic RAS and BRAF Variants	wrong pubblication type	round I
rayyan-661155526	BRAF gene as a potential target to attenuate drug resistance and treat cancer	wrong pubblication type	round I
rayyan-661155532	European expert panel consensus on the clinical management of BRAFV600E-mutant metastatic colorectal cancer	wrong pubblication type	round I
rayyan-661155533	Serrated colorectal cancer: preclinical models and molecular pathways	wrong pubblication type	round I
rayyan-661155534	BRAF-mutated colorectal adenocarcinomas: Pathological heterogeneity and clinical implications	wrong pubblication type	round I
rayyan-661155537	High sensitivity and rapid detection of KRAS and BRAF gene mutations in colorectal cancer using YbTixOy electrolyte-insulator-semiconductor biosensors	wrong pubblication type	round I
rayyan-661155538	"Tucatinib plus trastuzumab for chemotherapy-refractory	wrong pubblication type	round I
rayyan-661155541	Mutant RAS and the tumor microenvironment as dual therapeutic targets for advanced colorectal cancer	wrong pubblication type	round I
rayyan-661155542	Phase II study of trifluridine/tipiracil plus bevacizumab by RAS mutation status in patients with metastatic colorectal cancer refractory to standard therapies: JFMC51-1702-C7	wrong pubblication type	round I

rayyan-661155545	Management of BRAF-mutant metastatic colorectal cancer: a review of treatment options and evidence-based guidelines	wrong pubblication type	round I
rayyan-661155550	Systematic review of randomised clinical trials and observational studies for patients with RAS wild-type or BRAFV600E-mutant metastatic and/or unresectable colorectal cancer	wrong pubblication type	round I
rayyan-661155553	Monitoring tumour resistance to the BRAF inhibitor combination regimen in colorectal cancer patients via circulating tumour DNA	wrong pubblication type	round I
rayyan-661155558	Exploring biological heterogeneity and implications on novel treatment paradigm in BRAF-mutant metastatic colorectal cancer	wrong pubblication type	round I
rayyan-661155559	The evolving role of microsatellite instability in colorectal cancer: A review	wrong pubblication type	round I
rayyan-661155561	Beyond EGFR inhibitors in advanced colorectal cancer: Targeting BRAF and HER2	wrong pubblication type	round I
rayyan-661155562	The mechanism investigation of mutation genes in liver and lung metastasis of colorectal cancer by using NGS technique	wrong pubblication type	round I
rayyan-661155565	Multiclonal colorectal cancers with divergent histomorphological features and RAS mutations: one cancer or separate cancers?	wrong pubblication type	round I
rayyan-661155566	BRAF mutations in melanoma and colorectal cancer: A single oncogenic mutation with different tumour phenotypes and clinical implications	wrong pubblication type	round I
rayyan-661155569	A review of targeted therapy and immune checkpoint inhibitors for metastatic colorectal cancer	wrong pubblication type	round I
rayyan-661155576	The predictive role of ERBB2 point mutations in metastatic colorectal cancer: A systematic review	wrong pubblication type	round I
rayyan-661155579	Targeting BRAF in metastatic colorectal cancer: Maximizing molecular approaches	wrong pubblication type	round I
rayyan-661155582	Immunopeptidomic Analyses of Colorectal Cancers With and Without Microsatellite Instability	wrong pubblication type	round I
rayyan-661155583	"Deficient mismatch repair/microsatellite unstable colorectal cancer: Diagnosis	wrong pubblication type	round I
rayyan-661155801	KRAS and BRAF Mutations in Stage II and III Colon Cancer: A Systematic Review and Meta-Analysis.	wrong pubblication type	round I
rayyan-661155804	RAF1 contributes to cell proliferation and STAT3 activation in colorectal cancer independently of microsatellite and KRAS status.	wrong pubblication type	round I
rayyan-661155807	Treatment of Advanced BRAF-Mutated Colorectal Cancer: Where We Are and Where We Are Going.	wrong pubblication type	round I

rayyan-661155808	Microsatellite instability and colorectal cancer.	wrong pubblication type	round I
rayyan-661155809	Targeting Colon Cancers with Mutated BRAF and Microsatellite Instability.	wrong pubblication type	round I
rayyan-661155814	Molecular and prognostic heterogeneity of microsatellite-unstable colorectal cancer.	wrong pubblication type	round I
rayyan-661155817	Recent advances in understanding colorectal cancer.	wrong pubblication type	round I
rayyan-661155818	Serrated pathway in colorectal carcinogenesis.	wrong pubblication type	round I
rayyan-661155823	Colorectal cancer: genetics of development and metastasis.	wrong pubblication type	round I
rayyan-661155825	Tracking the molecular features of nonpolypoid colorectal neoplasms: a systematic review and meta-analysis.	wrong pubblication type	round I
rayyan-661155826	"Smoking and Colorectal Cancer Risk	wrong pubblication type	round I
rayyan-661155833	CpG Island Methylator Phenotype-High Colorectal Cancers and Their Prognostic Implications and Relationships with the Serrated Neoplasia Pathway.	wrong pubblication type	round I
rayyan-661155841	Four molecular subtypes of colorectal cancer and their precursor lesions.	wrong pubblication type	round I
rayyan-661155848	Targeting the serrated pathway of colorectal cancer with mutation in BRAF.	wrong pubblication type	round I
rayyan-661155850	PIK3CA mutation and clinicopathological features of colorectal cancer: a systematic review and Meta-Analysis.	wrong pubblication type	round I
rayyan-661155885	Molecular markers predictive of chemotherapy response in colorectal cancer.	wrong pubblication type	round I
rayyan-661155887	Clinicopathological characteristics of serrated polyps as precursors to colorectal cancer: Current status and management.	wrong pubblication type	round I
rayyan-661155907	Chemotherapeutic effects of MEK kinase inhibitor and BRAF kinase inhibitor on KRAS-mutated human colon cancer cell lines with different microsatellite instability.	wrong pubblication type	round I
rayyan-661155914	The prognostic and predictive impact of BRAF mutations in deficient mismatch repair/microsatellite instability-high colorectal cancer: systematic review/meta-analysis.	wrong pubblication type	round I
rayyan-661155920	Amplicon-based NGS test for assessing MLH1 promoter methylation and its correlation with BRAF mutation in colorectal cancer patients.	wrong pubblication type	round I

rayyan-661155924	"Loss of heterozygosity	wrong pubblication type	round I
rayyan-661155925	Age-dependent performance of BRAF mutation testing in Lynch syndrome diagnostics.	wrong pubblication type	round I
rayyan-661155930	Biology and Therapeutic Targets of Colorectal Serrated Adenocarcinoma	wrong pubblication type	round I
rayyan-661155932	MSI-High RAS-BRAF wild-type colorectal adenocarcinomas with MLH1 loss have a high frequency of targetable oncogenic gene fusions whose diagnoses are feasible using methods easy-to-implement in pathology laboratories.	wrong pubblication type	round I
rayyan-661155943	The role of autophagy in the treatment of BRAF mutant colorectal carcinomas differs based on microsatellite instability status.	wrong pubblication type	round I
rayyan-661155944	[Colorectal Carcinoma with Suspected Lynch Syndrome: A Multidisciplinary Algorithm].	wrong pubblication type	round I
rayyan-661155945	Exome sequencing revealed comparable frequencies of RNF43 and BRAF mutations in Middle Eastern colorectal cancer.	wrong pubblication type	round I
rayyan-661155946	Unmasking the role of KRAS and BRAF pathways in MSI colorectal tumors.	wrong pubblication type	round I
rayyan-661155951	The Evolving Role of Consensus Molecular Subtypes: a Step Beyond Inpatient Selection for Treatment of Colorectal Cancer.	wrong pubblication type	round I
rayyan-661155954	"Association between methylation in mismatch repair genes	wrong pubblication type	round I
rayyan-661155956	Molecular Biomarkers according to Primary Tumor Location in Colorectal Cancer: Current Standard and New Insights.	wrong pubblication type	round I
rayyan-661155958	Adjuvant Chemotherapy With or Without Biologics Including Antiangiogenics and Monoclonal Antibodies Targeting EGFR and EpCAM in Colorectal Cancer: A Systematic Review and Meta-analysis.	wrong pubblication type	round I
rayyan-661155963	Meta-analysis of the molecular associations of mucinous colorectal cancer.	wrong pubblication type	round I
rayyan-661155969	MSI status is associated with distinct clinicopathological features in BRAF mutation colorectal cancer: A systematic review and meta-analysis.	wrong pubblication type	round I
rayyan-661155970	"[A New Era of Colorectal Cancer Treatment Being Led by a Precise Analysis of Somatic Mutational Profiles Including RAS	wrong pubblication type	round I
rayyan-661155993	"Pathological complete response with anti-PD-1 therapy in a patient with microsatellite instable high	wrong pubblication type	round I
rayyan-661155999	"Molecular biomarkers for an early diagnosis	wrong pubblication type	round I

rayyan-661156004	Molecular classification of colorectal cancer: a dream that can become a reality.	wrong pubblication type	round I
rayyan-661156016	Association between KRAS G13D mutations and anastomotic recurrence in colorectal cancer: Two case reports.	wrong pubblication type	round I
rayyan-661156047	Genetic prognostic and predictive markers in colorectal cancer.	wrong pubblication type	round I
rayyan-661156050	"Genetic alterations of APC	wrong pubblication type	round I
rayyan-661156051	Pathologic features and biologic importance of colorectal serrated polyps.	wrong pubblication type	round I
rayyan-661156072	"Serrated colorectal cancer: Molecular classification	wrong pubblication type	round I
rayyan-661156079	Primary signet ring cell carcinoma of the colon and rectum.	wrong pubblication type	round I
rayyan-661156082	The role of molecular markers in predicting response to therapy in patients with colorectal cancer.	wrong pubblication type	round I
rayyan-661156123	Prognostic role of PIK3CA mutation in colorectal cancer: cohort study and literature review.	wrong pubblication type	round I
rayyan-661156125	Clinical features and molecular alterations of traditional serrated adenoma in sporadic colorectal carcinogenesis.	wrong pubblication type	round I
rayyan-661156139	POLE somatic mutations in advanced colorectal cancer.	wrong pubblication type	round I
rayyan-661156144	Molecular subtyping of colorectal cancer: time to explore both intertumoral and intratumoral heterogeneity to evaluate patient outcome.	wrong pubblication type	round I
rayyan-661156148	Prognostic and Predictive Biomarkers in Colorectal Cancer: Implications for the Clinical Surgeon.	wrong pubblication type	round I
rayyan-661156163	Genetic aspects of non-polypoid colorectal neoplasms.	wrong pubblication type	round I
rayyan-661156167	Prognostic and predictive biomarkers in resected colon cancer: current status and future perspectives for integrating genomics into biomarker discovery.	wrong pubblication type	round I
rayyan-661156171	Biomarkers in early-stage colorectal cancer: ready for prime time?	wrong pubblication type	round I
rayyan-661156172	Filiform serrated adenomas: a clinicopathologic and immunophenotypic study of 18 cases.	wrong pubblication type	round I

rayyan-661156186	Is Adjuvant Chemotherapy Efficient in Colon Cancer with High Microsatellite Instability? A Look Towards the Future.	wrong pubblication type	round I
rayyan-661156194	Genetic and epigenetic changes in colon cancer.	wrong pubblication type	round I
rayyan-661156204	A state-wide population-based program for detection of lynch syndrome based upon immunohistochemical and molecular testing of colorectal tumours.	wrong pubblication type	round I
rayyan-661156205	Identification of individuals at risk for Lynch syndrome using targeted evaluations and genetic testing: National Society of Genetic Counselors and the Collaborative Group of the Americas on Inherited Colorectal Cancer joint practice guideline.	wrong pubblication type	round I
rayyan-661156213	[Colorectal carcinoma in consideration of the new German S3 guideline 2013].	wrong pubblication type	round I
rayyan-661156230	Surgical Resection for Local and Lateral Lymph Node Recurrence of MSI-high Cecal Cancer with the BRAF V600E Mutation.	wrong pubblication type	round I
rayyan-661156231	Positive association of PIK3CA mutation with KRAS mutation but not BRAF mutation in colorectal cancer suggests co-selection is gene specific but not pathway specific.	wrong pubblication type	round I
rayyan-661156233	Case of a Patient With Pancreatic Cancer With Sporadic Microsatellite Instability Associated With a BRAF Fusion Achieving Excellent Response to Immunotherapy.	wrong pubblication type	round I
rayyan-661156234	[Pathological diagnosis for individualized therapy of colorectal cancer].	wrong pubblication type	round I
rayyan-661156245	Development of invasive colon cancer with microsatellite instability in a patient with hyperplastic polyposis syndrome.	wrong pubblication type	round I
rayyan-661156246	ASCO 2006 update of recommendations for the use of tumor markers in gastrointestinal cancer.	wrong pubblication type	round I
rayyan-661156247	Combining molecular markers with the TNM staging system to improve prognostication in stage II and III colon cancer: are we ready yet?	wrong pubblication type	round I
rayyan-661156250	What can molecular pathology offer for optimal decision making?	wrong pubblication type	round I
rayyan-661156251	A novel case of rhabdoid colon carcinoma associated with a positive CpG island methylator phenotype and BRAF mutation.	wrong pubblication type	round I
rayyan-661156256	[Insights on colorectal carcinoma based on the biological differences between left-sided and right- sided colon cancers].	wrong pubblication type	round I
rayyan-661156262	Adjuvant Therapy for Colon Cancer: Small Steps Toward Precision Medicine.	wrong pubblication type	round I
rayyan-661156264	Homozygous PMS2 deletion causes a severe colorectal cancer and multiple adenoma phenotype without extraintestinal cancer.	wrong pubblication type	round I

rayyan-661156270	"De novo constitutional MLH1 epimutations confer early-onset colorectal cancer in two new sporadic Lynch syndrome cases	wrong pubblication type	round I
rayyan-661156273	"[The alternative way of colorectal cancer developing. The histogenetic and molecular features of serrated lesions (review	wrong pubblication type	round I
rayyan-661156275	"Population-based universal screening for Lynch syndrome: ready	wrong pubblication type	round I
rayyan-661156276	[Pathologic diagnosis of colorectal cancer in the era of personalized therapy].	wrong pubblication type	round I
rayyan-661156281	Molecular Characterization of Sessile Serrated Adenoma/Polyps From a Large African American Cohort.	wrong pubblication type	round I
rayyan-661156289	[Biomarker for colorectal cancer].	wrong pubblication type	round I
rayyan-661156292	[Gene mutation and DNA methylation in non-hereditary colon neoplasms].	wrong pubblication type	round I
rayyan-661156293	[Serrated lesions and carcinoma of colorectum].	wrong pubblication type	round I
rayyan-661156294	[Ways of handling surgical material from patients with colon cancer - standard and additional highly specialized research with colorectal cancer].	wrong pubblication type	round I
rayyan-661156295	[Present conditions and problems in gene diagnosis of colon neoplasms].	wrong pubblication type	round I
rayyan-661156297	Precision Therapy in RAS Mutant Colorectal Cancer.	wrong pubblication type	round I
rayyan-661156307	[Prognostic marker profiles for risk of distant metastases in colorectal cancer].	wrong pubblication type	round I
rayyan-661156313	Filiform polyposis: A benign entity? Case report and literature review.	wrong pubblication type	round I
rayyan-661156316	[Prognostic biomarkers for metastatic colorectal cancer].	wrong pubblication type	round I
rayyan-661156329	Molecular Testing for the Treatment of Advanced Colorectal Cancer: An Overview.	wrong pubblication type	round I
rayyan-661154035	Chapter 1 - Special Diagnostic Techniques in Surgical Pathology	background article	round I
rayyan-661154036	The natural history of adenomas	background article	round I
rayyan-661154037	20 - Molecular pathology	background article	round I

rayyan-661154038	Chapter 6 - Gastrointestinal System	background article	round I
rayyan-661154039	Chapter 164 - Adenocarcinoma of the Colon and Rectum	background article	round I
rayyan-661154040	Chapter Four - Implementing circulating tumor DNA analysis in a clinical laboratory: A user manual	background article	round I
rayyan-661154041	13 - Tumours and Tumour-like Lesions of the Liver	background article	round I
rayyan-661154042	The therapeutic landscape of hepatocellular carcinoma	background article	round I
rayyan-661154043	Chapter 12 - Genetics of Adrenal Tumors	background article	round I
rayyan-661154044	86 - Carcinoma of the Ovaries and Fallopian Tubes	background article	round I
rayyan-661154045	Chapitre 2 - Anatomie pathologique	background article	round I
rayyan-661154046	13 - Neoplasms of the Testis	background article	round I
rayyan-661154047	Chapter 4 - Tumor Heterogeneity	background article	round I
rayyan-661154049	5 - Neoplasia	background article	round I
rayyan-661154051	Chapter 3 - Pharmacogenetics of therapeutics	background article	round I
rayyan-661154052	"Chapter 6 - Pharmacoepigenetic Processors: Epigenetic Drugs	background article	round I
rayyan-661154053	Intraductal Papillary Mucinous Neoplasm: A Clinicopathologic Review	background article	round I
rayyan-661154054	Chapter Seven - Synergistic combination of oncolytic virotherapy with CAR T-cell therapy	background article	round I
rayyan-661154055	Chapter 57 - Pharmacology and Molecular Mechanisms of Antineoplastic Agents for Hematologic Malignancies	background article	round I
rayyan-661154056	Gastroenteropancreatic endocrine tumors	background article	round I
rayyan-661154057	Chapter 9 - Hereditary predisposition to uveal melanoma	background article	round I
rayyan-661154059	Architects meets Repairers: The interplay between homeobox genes and DNA repair	background article	round I
rayyan-661154062	CHAPTER 7 - Molecular Biology	background article	round I
rayyan-661154077	1 - Pancreatic Carcinoma: An Introduction	background article	round I
rayyan-661154089	Chapter 8 - Prognostic epigenetics	background article	round I
rayyan-661154103	"17 - Pathology	background article	round I
rayyan-661154119	14 - Genetic and Epigenetic Alterations in Cancer	background article	round I
rayyan-661154162	Chapter 4 - Targeted therapy: small molecules	background article	round I
rayyan-661154163	"The pathophysiology	background article	round I

rayyan-661154164	Chapter 8 - Oncologyâ€"Acquired	background article	round I
rayyan-661154165	Mechanisms of MTH1 inhibition-induced DNA strand breaks: The slippery slope from the oxidized nucleotide pool to genotoxic damage	background article	round I
rayyan-661154166	3 - Molecular Basis of Human Malignancy	background article	round I
rayyan-661154167	Human height genes and cancer	background article	round I
rayyan-661154168	Understanding preanalytical variables and their effects on clinical biomarkers of oncology and immunotherapy	background article	round I
rayyan-661154169	Chapter 11 - Predictive Value of Epigenetic Signatures	background article	round I
rayyan-661154170	51 - Biology of Lung Cancer	background article	round I
rayyan-661154171	Chapter 17 - The Molecular Biology of Cancer	background article	round I
rayyan-661154172	CpG Methylation Analysisâ€"Current Status of Clinical Assays and Potential Applications in Molecular Diagnostics: A Report of the Association for Molecular Pathology	background article	round I
rayyan-661154174	Cancer gene discovery in mouse and man	background article	round I
rayyan-661154175	Chapter 3 - Biomarkers in Oncology and Nephrology	background article	round I
rayyan-661154176	"Chapter 7 - Development	background article	round I
rayyan-661154177	The contribution of targeted therapy to the neoadjuvant chemoradiation of rectal cancer	background article	round I
rayyan-661154178	Genetic Diversity of Pancreatic Ductal Adenocarcinoma and Opportunities for Precision Medicine	background article	round I
rayyan-661154179	Chapter 5 - State of the Art and Future Direction for the Analysis of Cell-Free Circulating DNA	background article	round I
rayyan-661154181	Chapter Five - T Lymphocyteâ€"Based Cancer Immunotherapeutics	background article	round I
rayyan-661154182	Chapter 13 - Biomarkers of malignancy	background article	round I
rayyan-661154185	Chapter 11 - Genetics and Molecular Biology of Pancreatic and Biliary Cancers	background article	round I
rayyan-661154186	CHAPTER 3 - Genetics and Epigenetics in Cancer Biology	background article	round I
rayyan-661154187	Pancreatic Cancer: Pathology and Genetics	background article	round I
rayyan-661154188	Chapter 1 - Immune Checkpoint Inhibitors: Mechanisms and Emerging Therapeutic Opportunities	background article	round I
rayyan-661154190	Chapter 66 - Mechanisms of Gastrointestinal Malignancies	background article	round I
rayyan-661154192	"Chapter One - The Evolving	background article	round I
rayyan-661154193	Discovering novel valid biomarkers and drugs in patient-centric genomic trials: the new epoch of precision surgical oncology	background article	round I
rayyan-661154194	Molecular Pathologyâ€"Translating Research into Clinical Practice: An Expanding Frontier in Surgical Oncology	background article	round I

rayyan-661154195	Tackling the implementation gap for the uptake of NGS and advanced molecular diagnostics into healthcare systems	background article	round I
rayyan-661154196	"From Colonic Polyps to Colon Cancer: Pathophysiology	background article	round I
rayyan-661154197	Chapter 9B - Molecular pathology of pancreatic cancer and premalignant tumors	background article	round I
rayyan-661154198	Histology-agnostic approvals for antibodyâ€"drug conjugates in solid tumours: is the time ripe?	background article	round I
rayyan-661154199	Dissecting the genetic alterations involved in lung carcinogenesis	background article	round I
rayyan-661154200	Chapter 4 - Clinical decision support and molecular tumor boards	background article	round I
rayyan-661154201	"Tumour-agnostic efficacy and safety of selpercatinib in patients with RET fusion-positive solid tumours other than lung or thyroid tumours (LIBRETTO-001): a phase 1/2	background article	round I
rayyan-661154202	"Cancer immunotherapy resistance based on immune checkpoints inhibitors: Targets	background article	round I
rayyan-661154205	The current status and problems confronted in delivering precision medicine in Japan and Europe	background article	round I
rayyan-661154206	Overcoming malignant cell-based mechanisms of resistance to immune checkpoint blockade antibodies	background article	round I
rayyan-661154208	Invasive apocrine carcinoma of the breast: clinicopathologic features and comprehensive genomic profiling of 18 pure triple-negative apocrine carcinomas	background article	round I
rayyan-661154213	Combinatorial therapy in tumor microenvironment: Where do we stand?	background article	round I
rayyan-661154219	Disentangling the relationship between tumor genetic programs and immune responsiveness	background article	round I
rayyan-661154223	Chapter 2 - Cancer Epigenetics	background article	round I
rayyan-661154227	The crosstalk of the human microbiome in breast and colon cancer: A metabolomics analysis	background article	round I
rayyan-661154228	Chapter 4 - Immune Checkpoint Inhibitors in Gastrointestinal Malignancies	background article	round I
rayyan-661154229	Lessons from the Cancer Genome	background article	round I
rayyan-661154230	Prediction in Rectal Cancer	background article	round I
rayyan-661154231	RNA sequencing for research and diagnostics in clinical oncology	background article	round I
rayyan-661154234	Gastric Cancer in the Era of Precision Medicine	background article	round I
rayyan-661154239	The Case for Laboratory Developed Procedures: Quality and Positive Impact on Patient Care	background article	round I
rayyan-661154240	Complexity of genome sequencing and reporting: Next generation sequencing (NGS) technologies and implementation of precision medicine in real life	background article	round I
rayyan-661154243	Chapter Four - Immune checkpoint therapy and response biomarkers in non-small-cell lung cancer: Serum NY-ESO-1 and XAGE1 antibody as predictive and monitoring markers	background article	round I
rayyan-661154244	"Chapter 1 - Biology	background article	round I

rayyan-661154245	NTRK insights: best practices for pathologists	background article	round I
rayyan-661154246	Chapter 2 - Pharmacogenomics and Personalized Medicines in Cancer Treatment	background article	round I
rayyan-661154247	Current Challenges in Cancer Treatment	background article	round I
rayyan-661154250	The 2022 revision of the World Health Organization classification of tumors of the urinary system and male genital organs: advances and challenges	background article	round I
rayyan-661154252	Chapter 12 - Genomics and Molecular Profiling of Lung Cancer	background article	round I
rayyan-661154253	Gastric Cancer	background article	round I
rayyan-661154254	How Can Gastric Cancer Molecular Profiling Guide Future Therapies?	background article	round I
rayyan-661154256	Chapter 20 - Applications of molecular techniques in the clinical laboratory	background article	round I
rayyan-661154258	New agents on the horizon in gastric cancer	background article	round I
rayyan-661154259	Pancreatic ductal adenocarcinoma in the era of precision medicine	background article	round I
rayyan-661154260	Chapter 70 - Cancer of the Colon and Gastrointestinal Tract	background article	round I
rayyan-661154272	Chapter 34 - Cancer Epigenetics	background article	round I
rayyan-661154276	Chapter Two - Circulating Biomarkers in Malignant Melanoma	background article	round I
rayyan-661154277	Chapter 24 - Genetic Intratumor Heterogeneity	background article	round I
rayyan-661154279	"Clinical Next-Generation Sequencing Assays for Solid Tumors: Current Practices	background article	round I
rayyan-661154281	Chapter 2 - Antiangiogenic drugs: Chemosensitizers for combination cancer therapy	background article	round I
rayyan-661154282	Cyclin-Dependent Kinase Inhibitors and the Treatment of Gastrointestinal Cancers	background article	round I
rayyan-661154283	Mitochondria: The metabolic switch of cellular oncogenic transformation	background article	round I
rayyan-661154285	Belgian expert consensus for tumor-agnostic treatment of NTRK gene fusion-driven solid tumors with larotrectinib	background article	round I
rayyan-661154286	"Pathophysiology	background article	round I
rayyan-661154290	Targeted Therapies: The Rare Cancer Paradigm	background article	round I
rayyan-661154291	Towards Precision Medicine in the Clinic: From Biomarker Discovery to Novel Therapeutics	background article	round I
rayyan-661154293	Colon cancer	background article	round I
rayyan-661154294	Chapter Three - Canine models of human cancer: Bridging the gap to improve precision medicine	background article	round I
rayyan-661154297	Lack of predictive tools for conventional and targeted cancer therapy: Barriers to biomarker development and clinical translation	background article	round I
rayyan-661154299	Roles of fusion genes in digestive system cancers: Dawn for cancer precision therapy	background article	round I

rayyan-661154301	Anti-cancer drug resistance: Understanding the mechanisms through the use of integrative genomics and functional RNA interference	background article	round I
rayyan-661154303	Sebaceous lesions and their associated syndromes: Part II	background article	round I
rayyan-661154305	Chapter 1 - Epithelial ovarian cancer: Genomic landscape and evolving precision treatment	background article	round I
rayyan-661154306	Enhancing personalized immune checkpoint therapy by immune archetyping and pharmacological targeting	background article	round I
rayyan-661154307	Chapter 14 - Cancer Vaccines	background article	round I
rayyan-661154308	Chapter 1 - Current Trends in Cancer Therapy	background article	round I
rayyan-661154309	Tumor-on-chip modeling of organ-specific cancer and metastasis	background article	round I
rayyan-661154311	Chapter 2 - Prognosis and follow-up of CRC patients: Role of diagnostic and therapeutic delay	background article	round I
rayyan-661154312	CHAPTER 11 - Alteration in major cell signaling pathways in cancer	background article	round I
rayyan-661154314	"Clinical Update: Colon	background article	round I
rayyan-661154316	Application of PD-1 Blockade in Cancer Immunotherapy	background article	round I
rayyan-661154318	Adapting Clinical Paradigms to the Challenges of Cancer Clonal Evolution	background article	round I
rayyan-661154320	The emerging roles of NGS in clinical oncology and personalized medicine	background article	round I
rayyan-661154322	tRNA Deregulation and Its Consequences inÂ Cancer	background article	round I
rayyan-661154329	Mutations: Driver Versus Passenger	background article	round I
rayyan-661154330	Chapter 29 - Targeted molecular therapy: the cancer paradigm	background article	round I
rayyan-661154332	Personalized Colon Cancer Care in 2010	background article	round I
rayyan-661154333	Cancer molecular markers: A guide to cancer detection and management	background article	round I
rayyan-661154336	ESMO recommendations on the standard methods to detect RET fusions and mutations in daily practice and clinical research	background article	round I
rayyan-661154337	Targeted Therapy: Attacking Cancer with Molecular and Immunological Targeted Agents	background article	round I
rayyan-661154338	Basket trials: From tumour gnostic to tumour agnostic drug development	background article	round I
rayyan-661154339	"Small molecules	background article	round I
rayyan-661154341	Chapter Eleven - Anti-cancer drug molecules targeting cancer cell cycle and proliferation	background article	round I
rayyan-661154342	3 - Mechanisms of multidrug resistance in cancer	background article	round I
rayyan-661154343	Chapter 4 - Cancer Genetic Screening and Ethical Considerations for Precision Medicine	background article	round I
rayyan-661154344	15 - Systemic Therapy for Colon Cancer	background article	round I

rayyan-661154345	microRNAs in colon cancer: A roadmap for discovery	background article	round I
rayyan-661154347	Chapter 3 - Liquid biopsy: new challenges in the era of immunotherapy and precision oncology NGS and the other faces of molecular biology	background article	round I
rayyan-661154353	piRNA: A promising biomarker in early detection of gastrointestinal cancer	background article	round I
rayyan-661154354	"The Interaction of Genomics	background article	round I
rayyan-661154355	The current evidence for a biomarker-based approach in cancer of unknown primary	background article	round I
rayyan-661154360	Neuroendocrine Neoplasms: Genetics and Epigenetics	background article	round I
rayyan-661154361	"How do changes in the mtDNA and mitochondrial dysfunction influence cancer and cancer therapy? Challenges	background article	round I
rayyan-661154363	Chapter 36 - Oral administration of cytostatic drugs in the treatment of CRC	background article	round I
rayyan-661154364	Chapter 4 - Current clinically validated applications of liquid biopsy	background article	round I
rayyan-661154365	Chapter 40 - Immunology and immunotherapy in CRC	background article	round I
rayyan-661154370	Re-Evaluating Clonal Dominance in Cancer Evolution	background article	round I
rayyan-661154375	CHAPTER 3 - Etiology of cancer	background article	round I
rayyan-661154376	The clinical role of circulating free tumor DNA in gastrointestinal malignancy	background article	round I
rayyan-661154377	"Chapter Four - Immune Contexture	background article	round I
rayyan-661154378	A perspective on the role of autophagy in cancer	background article	round I
rayyan-661154379	75 - Cancer of the Rectum	background article	round I
rayyan-661154380	Large-scale pharmacogenomic studies and drug response prediction for personalized cancer medicine	background article	round I
rayyan-661154387	Chapter 163 - Colorectal Polyps and Polyposis Syndromes	background article	round I
rayyan-661154398	Lynch Syndromeâ˜†	background article	round I
rayyan-661154400	Age-related biological differences in childrenâ€™s and adolescentsâ€™ very rare tumors	background article	round I
rayyan-661154407	Dysregulation and crosstalk of cellular signaling pathways in colon carcinogenesis	background article	round I
rayyan-661154411	24 - MOLECULAR BIOLOGY OF COLORECTAL CANCER	background article	round I
rayyan-661154412	ERK2-Dependent Phosphorylation of CSN6 Is Critical in Colorectal Cancer Development	background article	round I
rayyan-661154414	Chapter 35 - Cancer Epigenetics	background article	round I
rayyan-661154415	Gut eukaryotic virome in colorectal carcinogenesis: Is that a trigger?	background article	round I
rayyan-661154417	Microbiome-driven carcinogenesis in colorectal cancer: Models and mechanisms	background article	round I

rayyan-661154421	Druggable targets meet oncogenic drivers: opportunities and limitations of target-based classification of tumors and the role of Molecular Tumor Boards	background article	round I
rayyan-661154423	Target therapy in cancer treatment	background article	round I
rayyan-661154424	Chapter 36 - Colorectal Cancer	background article	round I
rayyan-661154426	Contributions of molecular analysis to the diagnosis and treatment of gastrointestinal neoplasms	background article	round I
rayyan-661154427	Rectal cancer genomics	background article	round I
rayyan-661154428	Chapter 4 - Cancer prognosis and immune system	background article	round I
rayyan-661154434	Expression and genomic profiling of colorectal cancer	background article	round I
rayyan-661154435	Chapter 46 - Colorectal Cancer and Colon Cancer Screening	background article	round I
rayyan-661154436	The burgeoning role of cytochrome P450-mediated vitamin D metabolites against colorectal cancer	background article	round I
rayyan-661154437	Chapter 62 - Colorectal Cancer	background article	round I
rayyan-661154439	Universal encoding of pan-cancer histology by deep texture representations	background article	round I
rayyan-661154440	NTRK fusions in solid tumours: what every pathologist needs to know	background article	round I
rayyan-661154441	Colorectal Cancer Therapeutics and the Challenges of Applied Pharmacogenomics	background article	round I
rayyan-661154442	Chapter 8 - Predictive biomarkers of drug resistance in colorectal cancerâ€”Recent updates	background article	round I
rayyan-661154444	13 - Molecular Testing of Gastrointestinal Neoplasms	background article	round I
rayyan-661154446	Biomarkers for personalized medicine in GI cancers	background article	round I
rayyan-661154448	Molecular mechanistic pathway of colorectal carcinogenesis associated with intestinal microbiota	background article	round I
rayyan-661154450	77 - Colorectal Cancer	background article	round I
rayyan-661154461	ctDNA as a cancer biomarker: A broad overview	background article	round I
rayyan-661154462	Chapter 2 - Drug transporters in the development of multidrug resistance in colorectal cancer	background article	round I
rayyan-661154464	"Chapter 5 - Cancer screening and prevention: Sex and gender evidence in lung	background article	round I
rayyan-661154468	Precision diagnostics: integration of tissue pathology and genomics in cancer	background article	round I
rayyan-661154469	Molecular Pathology of Gastrointestinal Cancer	background article	round I
rayyan-661154470	Treatment-driven tumour heterogeneity and drug resistance: Lessons from solid tumours	background article	round I
rayyan-661154473	Targeting ERK1/2 protein-serine/threonine kinases in human cancers	background article	round I
rayyan-661154485	"Chapter 12 - Genetic and genomic medicine relevance to cancer prevention	background article	round I

rayyan-661154489	"The cell of cancer origin provides the most reliable roadmap to its diagnosis	background article	round I
rayyan-661154490	"Chapter 25 - Epigenetic profiling in cancer: triage	background article	round I
rayyan-661154494	"Oral tumor heterogeneity	background article	round I
rayyan-661154496	Chapter 7 - PDX Models of Colorectal Tumors	background article	round I
rayyan-661154500	Clinical Updates for Colon Cancer Care in 2022	background article	round I
rayyan-661154501	The role of epigenetic therapies in colorectal cancer	background article	round I
rayyan-661154502	The long road to colorectal cancer therapy: Searching for the right signals	background article	round I
rayyan-661154511	Prognostic markers in colorectal pathology: is morphology enough?	background article	round I
rayyan-661154512	Molecular Testing in Colorectal Carcinoma	background article	round I
rayyan-661154513	"Do more targets allow more cancer treatments	background article	round I
rayyan-661154515	Promising Targets and Drugs in Development for Colorectal Cancer	background article	round I
rayyan-661154517	Chapter Nine - Modeling colorectal cancers using multidimensional organoids	background article	round I
rayyan-661154525	Chapter 15 - Precision Nutrition and Cancer	background article	round I
rayyan-661154534	Chapter 6 - Colorectal Cancer Metastasis	background article	round I
rayyan-661154539	CHAPTER 3 - Colorectal Cancer: Pathogenesis and Risk Factors	background article	round I
rayyan-661154543	New insights into the molecular pathogenesis of colorectal cancer	background article	round I
rayyan-661154550	The management of colorectal liver metastases	background article	round I
rayyan-661154554	Hereditary Cancer and Cancer Predisposition Syndromes	background article	round I
rayyan-661154561	74 - Colorectal Cancer	background article	round I
rayyan-661154565	Chapter 4 - The Genetics and Epigenetics of Colorectal Cancer Health Disparity	background article	round I
rayyan-661154571	Chapter 17 - Pathophysiology roles and translational opportunities of miRNAs in colorectal cancer	background article	round I
rayyan-661154572	The pathology of colorectal polyps and cancers (including biopsy)	background article	round I
rayyan-661154573	Colorectal cancer	background article	round I
rayyan-661154575	Genome-guided discovery of cancer therapeutic targets	background article	round I
rayyan-661154576	Towards personalized medicine of colorectal cancer	background article	round I
rayyan-661154647	Clinicopathologic features and treatment advances in cancers with HER2 alterations	background article	round I

rayyan-661154652	Chapter Seven - Genomic and epigenomic biomarkers in colorectal cancer: From diagnosis to therapy	background article	round I
rayyan-661154660	The Chromosomal Instability Pathway in Colon Cancer	background article	round I
rayyan-661154661	Precision Medicine in Colorectal Surgery	background article	round I
rayyan-661154662	Genetic and Epigenetic Biomarkers of Colorectal Cancer	background article	round I
rayyan-661154671	Serum and tissue markers in colorectal cancer: State of art	background article	round I
rayyan-661154674	"Colorectal Cancer: Epidemiology	background article	round I
rayyan-661154780	Non-steroidal anti-inflammatory drugs and molecular carcinogenesis of colorectal carcinomas	background article	round I
rayyan-661154783	Translational lung cancer research	background article	round I
rayyan-661154791	Improving outcomes in colorectal cancer: Where do we go from here?	background article	round I
rayyan-661154800	Context mattersâ€"consensus molecular subtypes of colorectal cancer as biomarkers for clinical trials	background article	round I
rayyan-661154806	Chapter 46 - Signaling pathways in CRC	background article	round I
rayyan-661154809	Chapter 89 - Vitamin D and colorectal cancer	background article	round I
rayyan-661154820	Chapter 18 - Resistance to EGFR Targeting Treatments in Colorectal Cancer	background article	round I
rayyan-661154824	Poorly Differentiated Clusters: Clinical Impact in Colorectal Cancer	background article	round I
rayyan-661154828	"Immunotherapy for GI Cancers: Who Benefits	background article	round I
rayyan-661155040	DNA methylation and chromatin modifiers in colorectal cancer	background article	round I
rayyan-661155045	From Molecular Biology to Clinical Trials: Toward Personalized Colorectal Cancer Therapy	background article	round I
rayyan-661155048	Molecular Markers for Colorectal Cancer	background article	round I
rayyan-661155050	Colorectal cancer	background article	round I
rayyan-661155056	Assessing colorectal cancer heterogeneity: one step closer to tailored medicine	background article	round I
rayyan-661155059	Tumour markers in colorectal cancer: European Group on Tumour Markers (EGTM) guidelines for clinical use	background article	round I
rayyan-661155063	Current advances in detecting genetic and epigenetic biomarkers of colorectal cancer	background article	round I
rayyan-661155067	Systemic Therapy for Colon Cancer	background article	round I
rayyan-661155068	BRAF gene: From human cancers to developmental syndromes	background article	round I
rayyan-661155070	Chapter 12 - Targeting the altered tyrosine kinases in colorectal cancer: From inhibitors to drugs	background article	round I
rayyan-661155073	"Overview of biomarkers in metastatic colorectal cancer: Tumour	background article	round I

rayyan-661155074	Chapter Five - Immunotherapy in colorectal cancer	background article	round I
rayyan-661155081	Therapeutic modulation of k-ras signaling in colorectal cancer	background article	round I
rayyan-661155099	Improving the management of pain from advanced cancer in the community	background article	round I
rayyan-661155117	FOCUS4: Molecular selection of therapy in colorectal cancer	background article	round I
rayyan-661155121	5-ALA in bowel cancer surgery	background article	round I
rayyan-661155236	Chapter 49 - Colorectal carcinoma: From molecular pathology to clinical practice	background article	round I
rayyan-661155237	Molecular Biomarkers in the Personalized Treatment of Colorectal Cancer	background article	round I
rayyan-661155238	Proteomic characterization of the colorectal cancer response to chemoradiation and targeted therapies reveals potential therapeutic strategies	background article	round I
rayyan-661155245	Molecular Determinants of Gastrointestinal Cancers	background article	round I
rayyan-661155247	40 - Colorectal cancer	background article	round I
rayyan-661155249	Chapter 10 - Predictive â€œomicâ€ biomarkers of drug response: Colorectal cancer as a model	background article	round I
rayyan-661155250	Colorectal cancer screening â€“ Methodology	background article	round I
rayyan-661155252	Genomic heterogeneity in primary colorectal carcinomas and their metastases: born bad or brought up a villain?	background article	round I
rayyan-661155255	Genetic alterations in colorectal cancers with demethylation of insulin-like growth factor II	background article	round I
rayyan-661155257	Clinical relevance of EGFR- and KRAS-status in colorectal cancer patients treated with monoclonal antibodies directed against the EGFR	background article	round I
rayyan-661155258	Chapter 16 - Systemic treatment of localized colorectal cancer	background article	round I
rayyan-661155259	"The Unmet Needs of the Diagnosis	background article	round I
rayyan-661155260	Chapter 176 - Adjuvant and Neoadjuvant Therapy for Colorectal Cancer: Molecular-Based Therapy	background article	round I
rayyan-661155261	Overview of Colorectal Cancer Genetics	background article	round I
rayyan-661155271	Genotypic and phenotypic signatures to predict immune checkpoint blockade therapy response in patients with colorectal cancer	background article	round I
rayyan-661155276	Genomic and Transcriptomic Determinants of Therapy Resistance and Immune Landscape Evolution during Anti-EGFR Treatment in Colorectal Cancer	background article	round I
rayyan-661155280	"Colorectal cancer heterogeneity and targeted therapy: Clinical implications	background article	round I
rayyan-661155282	"Recent progress of targeted nanocarriers in diagnostic	background article	round I
rayyan-661155289	Clinical Implications of Colorectal Cancer Stem Cells in the Age of Single-Cell Omics and Targeted Therapies	background article	round I
rayyan-661155320	Challenging chemoresistant metastatic colorectal cancer: therapeutic strategies from the clinic and from the laboratory	background article	round I
rayyan-661155321	FOLFOXIRI Versus Doublet Regimens in Right-Sided Metastatic Colorectal Cancer: Focus on Subsequent Therapies and Impact on Overall Survival	background article	round I

rayyan-661155325	Molecular Taxonomy and Tumourigenesis of Colorectal Cancer	background article	round I
rayyan-661155330	Molecular Evaluation of Colorectal Adenocarcinoma: Current Practice and Emerging Concepts	background article	round I
rayyan-661155337	Early onset sporadic colorectal cancer: Worrisome trends and oncogenic features	background article	round I
rayyan-661155341	Molecular Diagnostics in Colorectal Carcinoma	background article	round I
rayyan-661155350	Colorectal Cancer: Pathology and Genetics	background article	round I
rayyan-661155369	KRAS insertions in colorectal cancer: What do we know about unusual KRAS mutations?	background article	round I
rayyan-661155374	Chapter 19 - Circulating DNA and Protein Biomarkers for the Treatment of Metastatic Colorectal Cancer with Tyrosine Kinase Inhibitors	background article	round I
rayyan-661155378	Updated Management of Colorectal Cancer Liver Metastases: Scientific Advances Driving Modern Therapeutic Innovations	background article	round I
rayyan-661155379	Molecular Diagnostics in Colorectal Carcinoma: Advances and Applications for 2018	background article	round I
rayyan-661155385	BRAF inhibitors in cancer therapy	background article	round I
rayyan-661155387	Genetics and Genetic Biomarkers in Sporadic Colorectal Cancer	background article	round I
rayyan-661155388	The current value of determining the mismatch repair status of colorectal cancer: A rationale for routine testing	background article	round I
rayyan-661155389	Chapter 33 - Molecularly targeted therapy in metastatic CRC	background article	round I
rayyan-661155390	Chapter 34 - A roadmap for medical treatment of metastatic CRC	background article	round I
rayyan-661155396	Sex and gender perspectives in colorectal cancer	background article	round I
rayyan-661155398	Molecular typing of colorectal cancer: applications in diagnosis and treatment	background article	round I
rayyan-661155409	Chapter 25 - Molecular testing in colorectal cancer	background article	round I
rayyan-661155413	Chapter 12 - Colorectal cancer	background article	round I
rayyan-661155415	Biomarker-driven and molecular targeted therapies for colorectal cancers	background article	round I
rayyan-661155420	Comprehensive genomic characterization of sporadic synchronous colorectal cancer: Implications for treatment optimization and clinical outcome	background article	round I
rayyan-661155424	Immunotherapy for Colorectal Cancer	background article	round I
rayyan-661155435	Clinical application of circulating tumour DNA in colorectal cancer	background article	round I
rayyan-661155436	The impact of microsatellite stability status in colorectal cancer	background article	round I
rayyan-661155450	Precision Oncology in Gastrointestinal and Colorectal Cancer Surgery	background article	round I
rayyan-661155455	Chapter 4 - Use of molecular markers and other personalized factors in treatment decisions for metastatic colorectal cancer	background article	round I
rayyan-661155456	RAS Mutations as Predictive Biomarkers in Clinical Management of Metastatic Colorectal Cancer	background article	round I

rayyan-661155458	Molecular Approach to Colorectal Carcinoma: Current Evidence and Clinical Application	background article	round I
rayyan-661155464	"RAS Signaling in Colorectal Carcinomas through Alteration of RAS	background article	round I
rayyan-661155479	23 - Colorectal cancer	background article	round I
rayyan-661155480	"BRAF mutant colorectal cancer: prognosis	background article	round I
rayyan-661155481	How we treat metastatic colorectal cancer	background article	round I
rayyan-661155492	Colorectal cancer	background article	round I
rayyan-661155516	Pathologic Evaluation of Therapeutic Biomarkers in Colorectal Adenocarcinoma	background article	round I
rayyan-661155528	Targeted Therapy for Colorectal Cancer	background article	round I
rayyan-661155581	Precision oncology for BRAF-mutant cancers with BRAF and MEK inhibitors: from melanoma to tissue-agnostic therapy	background article	round I
rayyan-661155820	Colorectal Cancer: Genetics is Changing Everything.	background article	round I
rayyan-661155827	Molecular diagnostics of colorectal cancer.	background article	round I
rayyan-661155831	Genetic unraveling of colorectal cancer.	background article	round I
rayyan-661155851	[Molecular pathology of colorectal cancer].	background article	round I
rayyan-661155931	BRAF mutation and microsatellite instability status in colonic and rectal carcinoma: context really does matter.	background article	round I
rayyan-661155933	Serrated adenoma: a distinct form of non-polypoid colorectal neoplasia?	background article	round I
rayyan-661155942	Predictive factors to targeted treatment in gastrointestinal carcinomas.	background article	round I
rayyan-661156042	K-Ras and MSI: potential markers of both patient prognosis and treatment efficacy.	background article	round I
rayyan-661156096	[Research progress of serrated polyposis syndrome].	background article	round I
rayyan-661156102	[Standardized and structured histopathological evaluation of colorectal polyps: a practical checklist against the background of the new WHO classification].	background article	round I
rayyan-661156103	[Evaluation of BRAF V600E Mutations in High-Level Microsatellite Instability(MSI-H)Colon Cancer - Comparison Between Genetic Testing and Immunohistochemical Staining].	background article	round I
rayyan-661156202	Genotyping of colorectal cancer for cancer precision medicine: Results from the IPH Center for Molecular Pathology.	background article	round I
rayyan-661156218	[How and when to search for microsatellite instability in colorectal cancer in 2008?].	background article	round I
rayyan-661156315	Genetic mechanisms in interval colon cancers.	background article	round I
rayyan-661154084	Chapter 99 - Vitamin D and Colon Cancer	wrong outcome	round I
rayyan-661154124	Pharmacogenetic screening for drug therapy: From single gene markers to decision making in the next generation sequencing era	wrong outcome	round I

rayyan-661154160	Non-tumor adjacent tissue of advanced stage from CRC shows activated antioxidant response	wrong outcome	round I
rayyan-661154220	"Circulating tumor DNA	wrong outcome	round I
rayyan-661154224	Peripheral Neutrophil to Lymphocyte Ratio Improves Prognostication in Colon Cancer	wrong outcome	round I
rayyan-661154236	Massively parallel sequencing analysis of mucinous ovarian carcinomas: genomic profiling and differential diagnoses	wrong outcome	round I
rayyan-661154270	Altered cancer metabolism in mechanisms of immunotherapy resistance	wrong outcome	round I
rayyan-661154275	Rectal cancer with synchronous liver metastases: Do we have a clear direction?	wrong outcome	round I
rayyan-661154287	Biomarker-Driven and Molecular Targeted Therapies for Hepatobiliary Cancers	wrong outcome	round I
rayyan-661154288	Available technologies and clinical applications of targeted chemotherapy in pancreatic cancer	wrong outcome	round I
rayyan-661154298	Precursors of urinary bladder cancer: molecular alterations and biomarkers	wrong outcome	round I
rayyan-661154349	Deregulation of cell signaling in cancer	wrong outcome	round I
rayyan-661154351	Medicinal plants of the genuses Salvia and Hypericum are sources of anticolon cancer compounds: Effects on PI3K/Akt and MAP kinases pathways	wrong outcome	round I
rayyan-661154389	Looking into the toxicity potential and clinical benefits of tyrosine kinase inhibitors (TKIs)	wrong outcome	round I
rayyan-661154433	Predictors of Survival after Yttrium-90 Radioembolization for Colorectal Cancer Liver Metastases	wrong outcome	round I
rayyan-661154579	Mismatch repairâ€“deficient colorectal cancer: a model of immunogenic and immune cellâ€“rich tumor despite nonsignificant programmed cell death ligand-1 expression in tumor cells	wrong outcome	round I
rayyan-661154592	Clonal and subclonal mutational landscapes in circulating tumor DNA in metastatic colorectal cancer: an exploratory analysis from the phase III PARADIGM study	wrong outcome	round I
rayyan-661154636	A Colorectal Tumor Organoid Library Demonstrates Progressive Loss of Niche Factor Requirements during Tumorigenesis	wrong outcome	round I
rayyan-661154781	A Study of Subcutaneous Nivolumab Monotherapy With or Without Recombinant Human Hyaluronidase PH20 (rHuPH20)	wrong outcome	round I
rayyan-661154849	Circulating Tumour DNA Based Decision for Adjuvant Treatment in Colon Cancer Stage II Evaluation	wrong outcome	round I
rayyan-661155069	Intratumoral morphological heterogeneity can be an indicator of genetic heterogeneity in colorectal cancer	wrong outcome	round I
rayyan-661155098	Effects of gene expression pattern and RAS/BRAF mutations on the course of colorectal cancer	wrong outcome	round I
rayyan-661155219	Phase III randomised double-blind placebo controlled study of rofecoxib (VIOXX) in colorectal cancer patients following potentially curable therapy	wrong outcome	round I
rayyan-661155240	Optimal detection of clinically relevant mutations in colorectal carcinoma: sample pooling overcomes intra-tumoral heterogeneity	wrong outcome	round I
rayyan-661155246	"AD80	wrong outcome	round I
rayyan-661155263	A Genomic Analysis Workflow for Colorectal Cancer Precision Oncology	wrong outcome	round I
rayyan-661155326	Molecular Profiling of Patients With Advanced Colorectal Cancer: Princess Margaret Cancer Centre Experience	wrong outcome	round I

rayyan-661155349	Mapping clinicopathological entities within colorectal mucinous adenocarcinomas: a hierarchical clustering approach	wrong outcome	round I
rayyan-661155371	KRAS and PIK3CA mutations in colorectal adenocarcinomas correlate with aggressive histological features and behavior	wrong outcome	round I
rayyan-661155375	Combined Analysis of COX-2 and p53 Expressions Reveals Synergistic Inverse Correlations with Microsatellite Instability and CpG Island Methylator Phenotype in Colorectal Cancer	wrong outcome	round I
rayyan-661155385	BRAF inhibitors in cancer therapy	wrong outcome	round I
rayyan-661155426	Transformer-based biomarker prediction from colorectal cancer histology: A large-scale multicentric study	wrong outcome	round I
rayyan-661155444	Microsatellite instability status affects gene expression profiles in early onset colorectal cancer patients	wrong outcome	round I
rayyan-661155459	Prognostic vs predictive molecular biomarkers in colorectal cancer: is KRAS and BRAF wild type status required for anti-EGFR therapy?	wrong outcome	round I
rayyan-661155464	"RAS Signaling in Colorectal Carcinomas through Alteration of RAS	wrong outcome	round I
rayyan-661155466	"KRAS	wrong outcome	round I
rayyan-661155470	Fast and label-free automated detection of microsatellite status in early colon cancer using artificial intelligence integrated infrared imaging	wrong outcome	round I
rayyan-661155478	Immunohistochemical detection of BRAF V600E mutant protein using the VE1 antibody in colorectal carcinoma is highly concordant with molecular testing but requires rigorous antibody optimization	wrong outcome	round I
rayyan-661155486	Analysis of DNA Mismatch Repair Proteins Expression and BRAF V600E Mutation in a Subset of Early- and Late-onset Colorectal Carcinoma Patients in Mexico	wrong outcome	round I
rayyan-661155501	BRAF V600E mutations in right-side colon cancer: Heterogeneity detected by liquid biopsy	wrong outcome	round I
rayyan-661155503	Molecular Profiling Provides Clinical Insights Into TargetedÂ andÂ Immunotherapies as Well as Colorectal CancerÂ Prognosis	wrong outcome	round I
rayyan-661155546	Tumor Location Is Associated With the Prevalence of Braf And Pik3ca Mutations in Patients with Wild-Type Ras Colorectal Cancer: A Prospective Multi-Center Cohort Study in Japan	wrong outcome	round I
rayyan-661155547	Impact of Institutional Universal Microsatellite-Instability (MSI) Reflex Testing on Molecular Profiling Differences Between Younger and Older Patients with Colorectal Cancer	wrong outcome	round I
rayyan-661155565	Multiclonal colorectal cancers with divergent histomorphological features and RAS mutations: one cancer or separate cancers?	wrong outcome	round I
rayyan-661155589	Association between clinicopathological characteristics and RAS mutation in colorectal cancer	wrong outcome	round I
rayyan-661155797	The consensus molecular subtypes of colorectal cancer.	wrong outcome	round I
rayyan-661155799	Microbial Community Heterogeneity Within Colorectal Neoplasia and its Correlation With Colorectal Carcinogenesis.	wrong outcome	round I
rayyan-661155810	Subtyping of microsatellite instability-high colorectal cancer.	wrong outcome	round I
rayyan-661155811	Fusobacterium nucleatum Load Correlates with KRAS Mutation and Sessile Serrated Pathogenesis in Colorectal Adenocarcinoma.	wrong outcome	round I
rayyan-661155812	Irinotecan or Oxaliplatin: Which is the First Move for the Mate?	wrong outcome	round I

rayyan-661155813	KRAS mutated colorectal cancers with or without PIK3CA mutations: Clinical and molecular profiles inform current and future therapeutics.	wrong outcome	round I
rayyan-661155815	"Correlations among KRAS Mutation	wrong outcome	round I
rayyan-661155816	Microsatellite Instability in Greek Colorectal Carcinoma Patients: Clinicopathological and Molecular Correlations.	wrong outcome	round I
rayyan-661155819	"KRAS	wrong outcome	round I
rayyan-661155821	Colorectal serrated adenocarcinoma.	wrong outcome	round I
rayyan-661155824	"Epidemiological and molecular evaluation of BRAF	wrong outcome	round I
rayyan-661155829	Frequency and Clinicopathological Characteristics of Patients With KRAS/BRAF Double-Mutant Colorectal Cancer: An In Silico Study.	wrong outcome	round I
rayyan-661155836	"Molecular Profiling Based on KRAS/BRAF Mutation	wrong outcome	round I
rayyan-661155839	Metabolic factors and the risk of colorectal cancer by KRAS and BRAF mutation status.	wrong outcome	round I
rayyan-661155844	Postmenopausal hormone replacement therapy and colorectal cancer risk by molecular subtypes and pathways.	wrong outcome	round I
rayyan-661155853	BRAF and NRAS Locus-Specific Variants Have Different Outcomes on Survival to Colorectal Cancer.	wrong outcome	round I
rayyan-661155855	BRAF mutation in sporadic colorectal cancer and Lynch syndrome.	wrong outcome	round I
rayyan-661155856	Mutational profiling of colorectal cancers with microsatellite instability.	wrong outcome	round I
rayyan-661155863	KRAS and BRAF gene mutations and DNA mismatch repair status in Chinese colorectal carcinoma patients.	wrong outcome	round I
rayyan-661155866	BRAF-mutated microsatellite stable colorectal carcinoma: an aggressive adenocarcinoma with reduced CDX2 and increased cytokeratin 7 immunohistochemical expression.	wrong outcome	round I
rayyan-661155869	Opposing roles by KRAS and BRAF mutation on immune cell infiltration in colorectal cancer - possible implications for immunotherapy.	wrong outcome	round I
rayyan-661155871	Distinct BRAF (V600E) and KRAS mutations in high microsatellite instability sporadic colorectal cancer in African Americans.	wrong outcome	round I
rayyan-661155880	Mucinous adenocarcinoma of the colon and rectum: A genomic analysis.	wrong outcome	round I
rayyan-661155881	"p53 mutation is common in microsatellite stable	wrong outcome	round I
rayyan-661155882	Differential expression of CD133 based on microsatellite instability status in human colorectal cancer.	wrong outcome	round I
rayyan-661155884	Colorectal carcinomas with KRAS mutation are associated with distinctive morphological and molecular features.	wrong outcome	round I
rayyan-661155886	The role of heavy metals in the development of colorectal cancer.	wrong outcome	round I
rayyan-661155896	Microsatellite instability-low colorectal cancer acquires a KRAS mutation during the progression from Dukes' A to Dukes' B.	wrong outcome	round I
rayyan-661155897	Dietary Patterns and Risk of Colorectal Cancer: Analysis by Tumor Location and Molecular Subtypes.	wrong outcome	round I
rayyan-661155898	Association Between Smoking and Molecular Subtypes of Colorectal Cancer.	wrong outcome	round I

rayyan-661155902	Does neoadjuvant therapy alter KRAS and/or MSI results in rectal adenocarcinoma testing?	wrong outcome	round I
rayyan-661155903	"CpG island methylator phenotype	wrong outcome	round I
rayyan-661155904	Influence of microsatellite instability and KRAS and BRAF mutations on lymph node harvest in stage I-III colon cancers.	wrong outcome	round I
rayyan-661155905	Risk Factors and Incidence of Colorectal Cancer According to Major Molecular Subtypes.	wrong outcome	round I
rayyan-661155915	RNF43 germline and somatic mutation in serrated neoplasia pathway and its association with BRAF mutation.	wrong outcome	round I
rayyan-661155916	Predictive model for high-frequency microsatellite instability in colorectal cancer patients over 50Â years of age.	wrong outcome	round I
rayyan-661155919	Comprehensive Genomic Landscapes in Early and Later Onset Colorectal Cancer.	wrong outcome	round I
rayyan-661155922	Clinicopathological features of mismatch repair protein expression patterns in colorectal cancer.	wrong outcome	round I
rayyan-661155927	Mutations in both KRAS and BRAF may contribute to the methylator phenotype in colon cancer.	wrong outcome	round I
rayyan-661155934	Mutation profiling of cancer drivers in Brazilian colorectal cancer.	wrong outcome	round I
rayyan-661155935	"Microsatellite instability	wrong outcome	round I
rayyan-661155938	"Exclusive KRAS mutation in microsatellite-unstable human colorectal carcinomas with sequence alterations in the DNA mismatch repair gene	wrong outcome	round I
rayyan-661155943	The role of autophagy in the treatment of BRAF mutant colorectal carcinomas differs based on microsatellite instability status.	wrong outcome	round I
rayyan-661155947	"Intake of Dietary Fruit	wrong outcome	round I
rayyan-661155961	Development of sporadic microsatellite instability in colorectal tumors involves hypermethylation at methylated-in-tumor loci in adenoma.	wrong outcome	round I
rayyan-661155964	"Genetic instability	wrong outcome	round I
rayyan-661155967	Frequent PIK3CA Mutations in Colorectal and Endometrial Tumors With 2 or More Somatic Mutations in Mismatch Repair Genes.	wrong outcome	round I
rayyan-661155972	MLH1-deficient Colorectal Carcinoma With Wild-type BRAF and MLH1 Promoter Hypermethylation Harbor KRAS Mutations and Arise From Conventional Adenomas.	wrong outcome	round I
rayyan-661155977	"Gene methylation of SFRP2	wrong outcome	round I
rayyan-661155979	"Impact of BRAF	wrong outcome	round I
rayyan-661155982	The impact of CpG island methylator phenotype and microsatellite instability on tumour budding in colorectal cancer.	wrong outcome	round I
rayyan-661155983	Differences in histological features and PD-L1 expression between sporadic microsatellite instability and Lynch-syndrome-associated disease in Japanese patients with colorectal cancer.	wrong outcome	round I
rayyan-661155985	Chk1 frameshift mutation in sporadic and hereditary non-polyposis colorectal cancers with microsatellite instability.	wrong outcome	round I
rayyan-661155986	Mutations in BRAF and KRAS differentially distinguish serrated versus non-serrated hyperplastic aberrant crypt foci in humans.	wrong outcome	round I
rayyan-661155987	Clinicopathologic characteristics of FBXW7-mutated colorectal adenocarcinoma and association with aberrant beta-catenin localization.	wrong outcome	round I

rayyan-661155990	"TP53 mutations in colorectal cancer from Tunisia: relationships with site of tumor origin	wrong outcome	round I
rayyan-661155991	A distinct DNA methylation profile associated with microsatellite and chromosomal stable sporadic colorectal cancers.	wrong outcome	round I
rayyan-661155994	Unique clinicopathologic and genetic alteration features in early onset colorectal carcinoma compared with age-related colorectal carcinoma: a large cohort next generation sequence analysis.	wrong outcome	round I
rayyan-661155996	MUC5AC hypomethylation is a predictor of microsatellite instability independently of clinical factors associated with colorectal cancer.	wrong outcome	round I
rayyan-661156000	Molecular subtypes of colorectal cancers determined by PCR-based analysis.	wrong outcome	round I
rayyan-661156001	AKT1 E17K in Colorectal Carcinoma Is Associated with BRAF V600E but Not MSI-H Status: A Clinicopathologic Comparison to PIK3CA Helical and Kinase Domain Mutants.	wrong outcome	round I
rayyan-661156002	Multivariate analysis as a method for evaluating the pathogenicity of novel genetic MLH1 variants in patients with colorectal cancer and microsatellite instability.	wrong outcome	round I
rayyan-661156003	"Relationship between Fusobacterium nucleatum	wrong outcome	round I
rayyan-661156006	"MicroRNA-31 expression in relation to BRAF mutation	wrong outcome	round I
rayyan-661156009	Loss of nuclear p27 (CDKN1B/KIP1) in colorectal cancer is correlated with microsatellite instability and CIMP.	wrong outcome	round I
rayyan-661156012	Immunohistochemical staining for p16 and BRAFV600E is useful to distinguish between sporadic and hereditary (Lynch syndrome-related) microsatellite instable colorectal carcinomas.	wrong outcome	round I
rayyan-661156019	Clinical significance of fibroblast growth factor receptor 2 expression in patients with residual rectal cancer after preoperative chemoradiotherapy: relationship with KRAS or BRAF mutations and MSI status.	wrong outcome	round I
rayyan-661156021	DNA methylation alterations of AXIN2 in serrated adenomas and colon carcinomas with microsatellite instability.	wrong outcome	round I
rayyan-661156025	A Changing Spectrum of Colorectal Cancer Biology With Age: Implications for the Young Patient.	wrong outcome	round I
rayyan-661156026	Somatic molecular changes and histo-pathological features of colorectal cancer in Tunisia.	wrong outcome	round I
rayyan-661156030	Clinicopathologic and molecular characteristics of synchronous colorectal cancers: heterogeneity of clinical outcome depending on microsatellite instability status of individual tumors.	wrong outcome	round I
rayyan-661156040	Expression of the MAP kinase phosphatase DUSP4 is associated with microsatellite instability in colorectal cancer (CRC) and causes increased cell proliferation.	wrong outcome	round I
rayyan-661156041	Characterisation of PD-L1-positive subsets of microsatellite-unstable colorectal cancers.	wrong outcome	round I
rayyan-661156046	"P53 mutations in colorectal cancer from northern Iran: Relationships with site of tumor origin	wrong outcome	round I
rayyan-661156050	"Genetic alterations of APC	wrong outcome	round I
rayyan-661156053	Distinct molecular features of colorectal cancer in Ghana.	wrong outcome	round I
rayyan-661156055	MLH1-silenced and non-silenced subgroups of hypermutated colorectal carcinomas have distinct mutational landscapes.	wrong outcome	round I

rayyan-661156058	DNA sequence profiles of the colorectal cancer critical gene set KRAS-BRAF-PIK3CA-PTEN-TP53 related to age at disease onset.	wrong outcome	round I
rayyan-661156061	Biomarker testing and mutation prevalence in metastatic colorectal cancer patients in five European countries using a large oncology database.	wrong outcome	round I
rayyan-661156062	Is tumor testing efficiency for Lynch syndrome different in rectal and colon cancer?	wrong outcome	round I
rayyan-661156067	Down-regulation of p21 (CDKN1A/CIP1) is inversely associated with microsatellite instability and CpG island methylator phenotype (CIMP) in colorectal cancer.	wrong outcome	round I
rayyan-661156068	Prevalence and prognostic relevance of BrafV600E mutation in colorectal carcinomas from Kashmir (North India) valley.	wrong outcome	round I
rayyan-661156070	Cigarette smoking and colorectal cancer risk by molecularly defined subtypes.	wrong outcome	round I
rayyan-661156074	Rectal Aberrant Crypt Foci in Humans Are Not Surrogate Markers for Colorectal Cancer Risk.	wrong outcome	round I
rayyan-661156075	NRAS mutations are rare in colorectal cancer.	wrong outcome	round I
rayyan-661156078	Proximal colon cancers and the serrated pathway: a systematic analysis of precursor histology and BRAF mutation status.	wrong outcome	round I
rayyan-661156080	Molecular profile and copy number analysis of sporadic colorectal cancer in Taiwan.	wrong outcome	round I
rayyan-661156085	Distinct molecular features of different macroscopic subtypes of colorectal neoplasms.	wrong outcome	round I
rayyan-661156087	"Left-sided early-onset vs late-onset colorectal carcinoma: histologic	wrong outcome	round I
rayyan-661156088	BRAFV600E immunohistochemistry facilitates universal screening of colorectal cancers for Lynch syndrome.	wrong outcome	round I
rayyan-661156089	"Smoking	wrong outcome	round I
rayyan-661156090	Tumor and Patient Characteristics of Individuals with Mismatch Repair Deficient Colorectal Cancer.	wrong outcome	round I
rayyan-661156092	Validation microsatellite path score in a population-based cohort of patients with colorectal cancer.	wrong outcome	round I
rayyan-661156093	The role of the CpG island methylator phenotype in colorectal cancer prognosis depends on microsatellite instability screening status.	wrong outcome	round I
rayyan-661156095	"SMO expression in colorectal cancer: associations with clinical	wrong outcome	round I
rayyan-661156097	"Fatty acid synthase overexpression in colorectal cancer is associated with microsatellite instability	wrong outcome	round I
rayyan-661156100	Postmenopausal hormone therapy and colorectal cancer risk by molecularly defined subtypes among older women.	wrong outcome	round I
rayyan-661156101	[Molecular analysis of sporadic colon cancer].	wrong outcome	round I
rayyan-661156104	Differences in K-ras and mitochondrial DNA mutations and microsatellite instability between colorectal cancers of Vietnamese and Japanese patients.	wrong outcome	round I
rayyan-661156105	NDRG4 stratifies the prognostic value of body mass index in colorectal cancer.	wrong outcome	round I
rayyan-661156106	Association of HPV with genetic and epigenetic alterations in colorectal adenocarcinoma from Indian population.	wrong outcome	round I
rayyan-661156108	Annexin A10 expression correlates with serrated pathway features in colorectal carcinoma with microsatellite instability.	wrong outcome	round I

rayyan-661156112	Somatic BRAF-V600E mutations in familial colorectal cancer.	wrong outcome	round I
rayyan-661156113	"Filiform serrated adenoma is an unusual	wrong outcome	round I
rayyan-661156114	"An integrated analysis of lymphocytic reaction	wrong outcome	round I
rayyan-661156120	Whole-genome methylation analysis of benign and malignant colorectal tumours.	wrong outcome	round I
rayyan-661156121	Oncogenetic tree model of somatic mutations and DNA methylation in colon tumors.	wrong outcome	round I
rayyan-661156122	"Traditional Serrated Pathway-associated Colorectal Carcinoma: Morphologic Reappraisal of Serrated Morphology	wrong outcome	round I
rayyan-661156124	KRAS gene mutation in colorectal cancer is correlated with increased proliferation and spontaneous apoptosis.	wrong outcome	round I
rayyan-661156125	Clinical features and molecular alterations of traditional serrated adenoma in sporadic colorectal carcinogenesis.	wrong outcome	round I
rayyan-661156126	"Influence of anatomical subsite on the incidence of microsatellite instability	wrong outcome	round I
rayyan-661156132	RNF43 mutation is associated with aggressive tumor biology along with BRAF V600E mutation in right-sided colorectal cancer.	wrong outcome	round I
rayyan-661156133	"Association between risk factors	wrong outcome	round I
rayyan-661156140	Clinically important molecular features of Peruvian colorectal tumours: high prevalence of DNA mismatch repair deficiency and low incidence of KRAS mutations.	wrong outcome	round I
rayyan-661156143	The clinicopathological features and prognosis of tumor MSI in East Asian colorectal cancer patients using NCI panel.	wrong outcome	round I
rayyan-661156149	Pattern of clinically relevant mutations in consecutive series of Russian colorectal cancer patients.	wrong outcome	round I
rayyan-661156150	Frequency and application of the hot spot BRAF gene mutation (p.V600E) in the diagnostic strategy for Hereditary Nonpolyposis Colorectal Cancer.	wrong outcome	round I
rayyan-661156153	Van-Gogh-like 2 antagonises the canonical WNT pathway and is methylated in colorectal cancers.	wrong outcome	round I
rayyan-661156155	Prevalence of somatic mutl homolog 1 promoter hypermethylation in Lynch syndrome colorectal cancer.	wrong outcome	round I
rayyan-661156156	Wild-type APC predicts poor prognosis in microsatellite-stable proximal colon cancer.	wrong outcome	round I
rayyan-661156157	The genetic basis of colorectal cancer in a population-based incident cohort with a high rate of familial disease.	wrong outcome	round I
rayyan-661156158	Lower prevalence of Lynch syndrome in colorectal cancer patients in a Japanese hospital-based population.	wrong outcome	round I
rayyan-661156161	Population-based molecular screening for Lynch syndrome: implications for personalized medicine.	wrong outcome	round I
rayyan-661156162	Aberrant pattern of the cytokeratin 7/cytokeratin 20 immunophenotype in colorectal adenocarcinomas with BRAF mutations.	wrong outcome	round I
rayyan-661156164	Non-CpG island promoter hypomethylation and miR-149 regulate the expression of SRPX2 in colorectal cancer.	wrong outcome	round I
rayyan-661156175	Molecular genetic changes associated with colorectal carcinogenesis are not prognostic for tumor regression following preoperative chemoradiation of rectal carcinoma.	wrong outcome	round I
rayyan-661156176	"Cytoplasmic localization of p27 (cyclin-dependent kinase inhibitor 1B/KIP1) in colorectal cancer: inverse correlations with nuclear p27 loss	wrong outcome	round I

rayyan-661156177	Correlation between hypermethylation of the RASSF2A promoter and K-ras/BRAF mutations in microsatellite-stable colorectal cancers.	wrong outcome	round I
rayyan-661156178	Involvement of the serrated neoplasia pathway in inflammatory bowel disease-related colorectal oncogenesis.	wrong outcome	round I
rayyan-661156179	Copy number of the Adenomatous Polyposis Coli gene is not always neutral in sporadic colorectal cancers with loss of heterozygosity for the gene.	wrong outcome	round I
rayyan-661156180	Assessment of colorectal cancer molecular features along bowel subsites challenges the conception of distinct dichotomy of proximal versus distal colorectum.	wrong outcome	round I
rayyan-661156181	Transcriptional profiles underpin microsatellite status and associated features in colon cancer.	wrong outcome	round I
rayyan-661156187	"Cyclin D1 is frequently overexpressed in microsatellite unstable colorectal cancer	wrong outcome	round I
rayyan-661156200	Associations between intake of folate and related micronutrients with molecularly defined colorectal cancer risks in the Iowa Women's Health Study.	wrong outcome	round I
rayyan-661156207	Hereditary non-polyposis colorectal cancer/Lynch syndrome in three dimensions.	wrong outcome	round I
rayyan-661156209	Comprehensive profiling of DNA methylation in colorectal cancer reveals subgroups with distinct clinicopathological and molecular features.	wrong outcome	round I
rayyan-661156214	TGFBR2 mutation is correlated with CpG island methylator phenotype in microsatellite instability-high colorectal cancer.	wrong outcome	round I
rayyan-661156215	The methylator phenotype in microsatellite stable colorectal cancers is characterized by a distinct gene expression profile.	wrong outcome	round I
rayyan-661156220	Comprehensive biostatistical analysis of CpG island methylator phenotype in colorectal cancer using a large population-based sample.	wrong outcome	round I
rayyan-661156224	Novel application of structural equation modeling to correlation structure analysis of CpG island methylation in colorectal cancer.	wrong outcome	round I
rayyan-661156227	Correlation of beta-catenin localization with cyclooxygenase-2 expression and CpG island methylator phenotype (CIMP) in colorectal cancer.	wrong outcome	round I
rayyan-661156234	[Pathological diagnosis for individualized therapy of colorectal cancer].	wrong outcome	round I
rayyan-661156237	Tumor Budding in Colorectal Carcinoma: Confirmation of Prognostic Significance and Histologic Cutoff in a Population-based Cohort.	wrong outcome	round I
rayyan-661156257	The utility of diagnostic biopsy specimens for predictive molecular testing in colorectal cancer.	wrong outcome	round I
rayyan-661156258	Mutational analysis of the BRAF gene in colorectal mucinous carcinoma in association with histological configuration.	wrong outcome	round I
rayyan-661156260	[Molecular characterization of hereditary colorectal cancer in Peru].	wrong outcome	round I
rayyan-661156268	Taiwan hospital-based detection of Lynch syndrome distinguishes 2 types of microsatellite instabilities in colorectal cancers.	wrong outcome	round I
rayyan-661156269	"Association of smoking	wrong outcome	round I
rayyan-661156271	Frequency of defective DNA mismatch repair in colorectal cancer among the Alaska Native people.	wrong outcome	round I
rayyan-661156274	Molecular biology from bench-to-bedside - which colorectal cancer patients should be referred for genetic counselling and risk assessment.	wrong outcome	round I
rayyan-661156280	Evaluation of predictive models in daily practice for the identification of patients with Lynch syndrome.	wrong outcome	round I
rayyan-661156283	"A population-based study of age-related variation in clinicopathological features	wrong outcome	round I

rayyan-661156299	BRAF V600E-specific immunohistochemistry for the exclusion of Lynch syndrome in MSI-H colorectal cancer.	wrong outcome	round I
rayyan-661156304	Implementation of universal microsatellite instability and immunohistochemistry screening for diagnosing lynch syndrome in a large academic medical center.	wrong outcome	round I
rayyan-661156306	Diet and lifestyle factor associations with CpG island methylator phenotype and BRAF mutations in colon cancer.	wrong outcome	round I
rayyan-661156308	"Early onset MSI-H colon cancer with MLH1 promoter methylation	wrong outcome	round I
rayyan-661156309	Molecular genetic analysis of 103 sporadic colorectal tumours in Czech patients.	wrong outcome	round I
rayyan-661156310	"Somatic alterations	wrong outcome	round I
rayyan-661156311	BRAF mutation analysis is a valid tool to implement in Lynch syndrome diagnosis in patients classified according to the Bethesda guidelines.	wrong outcome	round I
rayyan-661156312	The MLH1 -93 G>A promoter polymorphism and genetic and epigenetic alterations in colon cancer.	wrong outcome	round I
rayyan-661156318	Lynch syndrome (hereditary nonpolyposis colorectal cancer) diagnostics.	wrong outcome	round I
rayyan-661156319	"KRAS	wrong outcome	round I
rayyan-661156320	Cost-Effectiveness Analysis of Different Genetic Testing Strategies for Lynch Syndrome in Taiwan.	wrong outcome	round I
rayyan-661156321	"Expression of MUC2	wrong outcome	round I
rayyan-661156322	Tissue biomarker development in a multicentre trial context: a feasibility study on the PETACC3 stage II and III colon cancer adjuvant treatment trial.	wrong outcome	round I
rayyan-661156323	Lynch-like syndrome is as frequent as Lynch syndrome in early-onset nonfamilial nonpolyposis colorectal cancer.	wrong outcome	round I
rayyan-661156327	"Simplified identification of Lynch syndrome: a prospective	wrong outcome	round I
rayyan-661154697	A phase Ib biomarker trial of naproxen in patients at risk for DNA mismatch repair deficient colorectal cancer	wrong study desing	round I
rayyan-661154698	Genetic variants of methyl metabolizing enzymes and epigenetic regulators: associations with promoter CpG island hypermethylation in colorectal cancer	wrong study desing	round I
rayyan-661154712	A study on microsatellite instability status for colorectal serrated lesions	wrong study desing	round I
rayyan-661154744	Hide and Seek with Hereditary Cancer: improving detection of colorectal cancer patients with a high risk of Lynch syndrome	wrong study desing	round I
rayyan-661154769	Effectiveness of each Bethesda marker in defining microsatellite instability when screening for Lynch syndrome	wrong study desing	round I
rayyan-661154771	Effects of a decision support intervention on decisional conflict associated with microsatellite instability testing	wrong study desing	round I
rayyan-661154773	Electronic reminders for pathologists promote recognition of patients at risk for Lynch syndrome: cluster-randomised controlled trial	wrong study desing	round I
rayyan-661154780	Non-steroidal anti-inflammatory drugs and molecular carcinogenesis of colorectal carcinomas	wrong study desing	round I
rayyan-661154791	Improving outcomes in colorectal cancer: Where do we go from here?	wrong study desing	round I
rayyan-661154802	Polymeric nanoparticles approach and identification and characterization of novel biomarkers for colon cancer	wrong study desing	round I

rayyan-661154810	An Update on Colorectal Cancer	wrong study desing	round I
rayyan-661154822	Precision Treatment and Prevention of Colorectal Cancerâ€"Hope or Hype?	wrong study desing	round I
rayyan-661154825	Pan-tropomyosin receptor kinase immunohistochemistry is a feasible routine screening strategy for NTRK fusions in mismatch repair-deficient colorectal carcinomas	wrong study desing	round I
rayyan-661154828	"Immunotherapy for GI Cancers: Who Benefits	wrong study desing	round I
rayyan-661154839	Traditional serrated adenoma with BRAF mutation is associated with synchronous/metachronous BRAF-mutated serrated lesions	wrong study desing	round I
rayyan-661154841	Clinical and Molecular Biologic Characteristics of Early-onset Versus Late-onset Colorectal Carcinoma in Filipinos	wrong study desing	round I
rayyan-661154842	Assessing tumor mutations to gain insight into base excision repair sequence polymorphisms and smoking in colon cancer	wrong study desing	round I
rayyan-661154849	Circulating Tumour DNA Based Decision for Adjuvant Treatment in Colon Cancer Stage II Evaluation	wrong study desing	round I
rayyan-661154979	Comparing international data between keyhole and robotic rectal cancer surgery	wrong study desing	round I
rayyan-661155274	KRAS Mutation Screening in Colorectal Cancer: From Paper to Practice	wrong study desing	round I
rayyan-661155314	BRAF in non-small cell lung cancer (NSCLC): Pickaxing another brick in the wall	wrong study desing	round I
rayyan-661155315	Clonal evolution and expansion associated with therapy resistance and relapse of colorectal cancer	wrong study desing	round I
rayyan-661155319	Prognosis in colorectal cancer beyond TNM	wrong study desing	round I
rayyan-661155320	Challenging chemoresistant metastatic colorectal cancer: therapeutic strategies from the clinic and from the laboratory	wrong study desing	round I
rayyan-661155327	Treatments after first progression in metastatic colorectal cancer. A literature review and evidence-based algorithm	wrong study desing	round I
rayyan-661155333	Colorectal cancer cell exosome and cytoplasmic membrane for homotypic delivery of therapeutic molecules	wrong study desing	round I
rayyan-661155336	Immunotherapy of colorectal cancer: Challenges for therapeutic efficacy	wrong study desing	round I
rayyan-661155338	A practical guide to biomarkers for the evaluation of colorectal cancer	wrong study desing	round I
rayyan-661155339	"A review of the sensitivity of metastatic colorectal cancer patients with deficient mismatch repair to standard-of-care chemotherapy and monoclonal antibodies	wrong study desing	round I
rayyan-661155342	Polymerase proofreading domain mutations: New opportunities for immunotherapy in hypermutated colorectal cancer beyond MMR deficiency	wrong study desing	round I
rayyan-661155344	The diverse molecular profiles of lynch syndrome-associated colorectal cancers are (highly) dependent on underlying germline mismatch repair mutations	wrong study desing	round I
rayyan-661155345	HER2 as a Predictive Biomarker and Treatment Target in Colorectal Cancer	wrong study desing	round I
rayyan-661155347	Overcoming dynamic molecular heterogeneity in metastatic colorectal cancer: Multikinase inhibition with regorafenib and the case of rechallenge with anti-EGFR	wrong study desing	round I
rayyan-661155362	Nano-enabled colorectal cancer therapy	wrong study desing	round I

rayyan-661155363	Genomic profiling of sporadic liver metastatic colorectal cancer	wrong study desing	round I
rayyan-661155365	HER2 targeted therapy in colorectal cancer: New horizons	wrong study desing	round I
rayyan-661155367	BRAF V600E mutation-specific antibody: A review	wrong study desing	round I
rayyan-661155370	Recent Advances in the Clinical Development of Immune Checkpoint Blockade Therapy for Mismatch Repair Proficient (pMMR)/non-MSI-H Metastatic Colorectal Cancer	wrong study desing	round I
rayyan-661155372	Small-molecule drugs of colorectal cancer: Current status and future directions	wrong study desing	round I
rayyan-661155373	"Melanoma with in-frame deletion of MAP2K1: a distinct molecular subtype of cutaneous melanoma mutually exclusive from BRAF	wrong study desing	round I
rayyan-661155377	Update on Mucoadhesive Approaches to target Drug Delivery in Colorectal Cancer	wrong study desing	round I
rayyan-661155381	Dichotomous colorectal cancer behaviour	wrong study desing	round I
rayyan-661155384	Immune scores in colorectal cancer: Where are we?	wrong study desing	round I
rayyan-661155394	The prognostic potential of CDX2 in colorectal cancer: Harmonizing biology and clinical practice	wrong study desing	round I
rayyan-661155395	Translational research of new developments in targeted therapy of colorectal cancer	wrong study desing	round I
rayyan-661155399	Clinicopathological relevance of BRAF mutations in human cancer	wrong study desing	round I
rayyan-661155400	The updates on metastatic mechanism and treatment of colorectal cancer	wrong study desing	round I
rayyan-661155405	Trifluridine/tipiracil (FTD/TPI) and regorafenib in older patients with metastatic colorectal cancer	wrong study desing	round I
rayyan-661155408	Liquid biopsy in colorectal cancer: Onward and upward	wrong study desing	round I
rayyan-661155412	Treatment sequencing of metastatic colorectal cancer based on primary tumor location	wrong study desing	round I
rayyan-661155414	Treatment sequencing in metastatic colorectal cancer	wrong study desing	round I
rayyan-661155415	Biomarker-driven and molecular targeted therapies for colorectal cancers	wrong study desing	round I
rayyan-661155416	Prognostic and predictive molecular biomarkers in advanced colorectal cancer	wrong study desing	round I
rayyan-661155418	"Colorectal cancer: Genetic alterations	wrong study desing	round I
rayyan-661155422	NeoRAS wild-type in metastatic colorectal cancer: Myth or truth?â€”Case series and review of the literature	wrong study desing	round I
rayyan-661155423	Strategic enhancement of immune checkpoint inhibition in refractory Colorectal Cancer: Trends and future prospective	wrong study desing	round I
rayyan-661155452	Targeting BRAF-mutant non-small cell lung cancer: Current status and future directions	wrong study desing	round I
rayyan-661155456	RAS Mutations as Predictive Biomarkers in Clinical Management of Metastatic Colorectal Cancer	wrong study desing	round I
rayyan-661155459	Prognostic vs predictive molecular biomarkers in colorectal cancer: is KRAS and BRAF wild type status required for anti-EGFR therapy?	wrong study desing	round I

rayyan-661155463	Immunotherapy in colorectal cancer: is the long-awaited revolution finally happening?	wrong study desing	round I
rayyan-661155465	Third- or Later-line Therapy for Metastatic Colorectal Cancer: Reviewing Best Practice	wrong study desing	round I
rayyan-661155466	"KRAS	wrong study desing	round I
rayyan-661155473	The EMA assessment of encorafenib in combination with cetuximab for the treatment of adult patients with metastatic colorectal carcinoma harbouring the BRAFV600E mutation who have received prior therapy	wrong study desing	round I
rayyan-661155478	Immunohistochemical detection of BRAF V600E mutant protein using the VE1 antibody in colorectal carcinoma is highly concordant with molecular testing but requires rigorous antibody optimization	wrong study desing	round I
rayyan-661155482	"Differences in Pathology and Mutation Status Among Colorectal Cancer Patients Younger Than	wrong study desing	round I
rayyan-661155483	Current progress of immune checkpoint inhibitors for advanced colorectal cancer: concentrating on the efficacy improvement	wrong study desing	round I
rayyan-661155484	Chromatin factors: Ready to roll as biomarkers in metastatic colorectal cancer?	wrong study desing	round I
rayyan-661155487	Prognostic and predictive markers in liver limited stage IV colorectal cancer	wrong study desing	round I
rayyan-661155488	Recommendations from the EGAPP Working Group: can testing of tumor tissue for mutations in EGFR pathway downstream effector genes in patients with metastatic colorectal cancer improve health outcomes by guiding decisions regarding anti-EGFR therapy?	wrong study desing	round I
rayyan-661155491	Metastatic colorectal cancer: Advances in the folate-fluoropyrimidine chemotherapy backbone	wrong study desing	round I
rayyan-661155492	Colorectal cancer	wrong study desing	round I
rayyan-661155500	Advances in systemic chemotherapy and immunotherapy for metastatic colorectal cancer	wrong study desing	round I
rayyan-661155503	Molecular Profiling Provides Clinical Insights Into TargetedÂ andÂ Immunotherapies as Well as Colorectal CancerÂ Prognosis	wrong study desing	round I
rayyan-661155534	BRAF-mutated colorectal adenocarcinomas: Pathological heterogeneity and clinical implications	wrong study desing	round I
rayyan-661155537	High sensitivity and rapid detection of KRAS and BRAF gene mutations in colorectal cancer using YbTixOy electrolyte-insulator-semiconductor biosensors	wrong study desing	round I
rayyan-661155824	"Epidemiological and molecular evaluation of BRAF	wrong study desing	round I
rayyan-661155852	Distinctive patterns of p53 protein expression and microsatellite instability in human colorectal cancer.	wrong study desing	round I
rayyan-661155862	Comprehensive characterization of RAS mutations in colon and rectal cancers in old and young patients.	wrong study desing	round I
rayyan-661155867	KRAS and BRAF mutations and MSI status in precursor lesions of colorectal cancer detected by colonoscopy.	wrong study desing	round I
rayyan-661155912	Efficacy and safety of neoadjuvant immunotherapy in patients with microsatellite instability-high gastrointestinal malignancies: A case series.	wrong study desing	round I
rayyan-661155913	BRAF(V600E) efficient transformation and induction of microsatellite instability versus KRAS(G12V) induction of senescence markers in human colon cancer cells.	wrong study desing	round I

rayyan-661155914	The prognostic and predictive impact of BRAF mutations in deficient mismatch repair/microsatellite instability-high colorectal cancer: systematic review/meta-analysis.	wrong study desing	round I
rayyan-661155916	Predictive model for high-frequency microsatellite instability in colorectal cancer patients over 50Â years of age.	wrong study desing	round I
rayyan-661155920	Amplicon-based NGS test for assessing MLH1 promoter methylation and its correlation with BRAF mutation in colorectal cancer patients.	wrong study desing	round I
rayyan-661155922	Clinicopathological features of mismatch repair protein expression patterns in colorectal cancer.	wrong study desing	round I
rayyan-661155929	Improved Detection of Microsatellite Instability in Early Colorectal Lesions.	wrong study desing	round I
rayyan-661155934	Mutation profiling of cancer drivers in Brazilian colorectal cancer.	wrong study desing	round I
rayyan-661155960	Colorectal Cancers with the Uncommon Findings of KRAS Mutation and Microsatellite Instability.	wrong study desing	round I
rayyan-661155978	Oncogenic BRAF mutation induces DNA methylation changes in a murine model for human serrated colorectal neoplasia.	wrong study desing	round I
rayyan-661155986	Mutations in BRAF and KRAS differentially distinguish serrated versus non-serrated hyperplastic aberrant crypt foci in humans.	wrong study desing	round I
rayyan-661155992	KRAS mutations in colorectal cancer from Tunisia: relationships with clinicopathologic variables and data on TP53 mutations and microsatellite instability.	wrong study desing	round I
rayyan-661156001	AKT1 E17K in Colorectal Carcinoma Is Associated with BRAF V600E but Not MSI-H Status: A Clinicopathologic Comparison to PIK3CA Helical and Kinase Domain Mutants.	wrong study desing	round I
rayyan-661156037	"Comprehensive analysis of CpG island methylator phenotype (CIMP)-high	wrong study desing	round I
rayyan-661156043	"Adenoma-like adenocarcinoma: a subtype of colorectal carcinoma with good prognosis	wrong study desing	round I
rayyan-661156052	"Comparison of microsatellite instability	wrong study desing	round I
rayyan-661156068	Prevalence and prognostic relevance of BrafV600E mutation in colorectal carcinomas from Kashmir (North India) valley.	wrong study desing	round I
rayyan-661156162	Aberrant pattern of the cytokeratin 7/cytokeratin 20 immunophenotype in colorectal adenocarcinomas with BRAF mutations.	wrong study desing	round I
rayyan-661156217	A genetic progression model of Braf(V600E)-induced intestinal tumorigenesis reveals targets for therapeutic intervention.	wrong study desing	round I
rayyan-661156219	"Tumor TP53 expression status	wrong study desing	round I
rayyan-661156229	The utility of BRAFV600E mutation-specific antibody for colon cancers with microsatellite instability.	wrong study desing	round I
rayyan-661156230	Surgical Resection for Local and Lateral Lymph Node Recurrence of MSI-high Cecal Cancer with the BRAF V600E Mutation.	wrong study desing	round I
rayyan-661156237	Tumor Budding in Colorectal Carcinoma: Confirmation of Prognostic Significance and Histologic Cutoff in a Population-based Cohort.	wrong study desing	round I
rayyan-661156243	Decreased Tumoral Expression of Colon-Specific Water Channel Aquaporin 8 Is Associated With Reduced Overall Survival in Colon Adenocarcinoma.	wrong study desing	round I
rayyan-661156272	CD8+ lymphocytes/ tumour-budding index: an independent prognostic factor representing a 'pro-/anti-tumour' approach to tumour host interaction in colorectal cancer.	wrong study desing	round I
rayyan-661156286	BRAF mutation is associated with the CpG island methylator phenotype in colorectal cancer from young patients.	wrong study desing	round I

rayyan-661156296	[Search for molecular marker/target molecule useful for colorectal cancer treatment].	wrong study desing	round I
rayyan-661156307	[Prognostic marker profiles for risk of distant metastases in colorectal cancer].	wrong study desing	round I
rayyan-661154045	Chapitre 2 - Anatomie pathologique	foreing language	round I
rayyan-661154085	A - ERKRANKUNGEN DER VERDAUUNGSORGANE	foreing language	round I
rayyan-661154833	Cancers du cÃ´lon : prise en charge molÃ©culaire	foreing language	round I
rayyan-661155518	Cancers colorectaux avec mutation V600E de BRAFÂ : oÃ¹ en sommes-nousÂ ?	foreing language	round I
rayyan-661156059	"[Impacts of microsatellite status	foreing language	round I
rayyan-661155888	Body size and risk for colorectal cancers showing BRAF mutations or microsatellite instability: a pooled analysis.	wrong drug	round I
rayyan-661154267	Oxaliplatin, fluorouracil, and leucovorin with or without cetuximab in patients with resected stage III colon cancer (PETACC-8): an open-label, randomised phase 3 trial	subsequent publication with the same patients	round II
rayyan-661154295	Comparison of 627 patients with right- and left-sided colon cancer in China: Differences in clinicopathology, recurrence, and survival	wrong outcomes not reported survival and recurrence data for primary outcome	round II
rayyan-661154369	Enhanced expression of GABRD predicts poor prognosis in patients with colon adenocarcinoma	wrong outcomes not reported survival and recurrence data for primary outcome	round II
rayyan-661154394	Dilemma of Stage II Colon Cancer and Decision Making for Adjuvant Chemotherapy	wrong article type including book chapter, review or molecoular phase I and II study	round II
rayyan-661154403	Effects of postoperative adjuvant chemotherapy and palliative chemotherapy on the gut microbiome in colorectal cancer	wrong outcomes not reported survival and recurrence data for primary outcome	round II
rayyan-661154429	Prognostic role of the LCS6 KRAS variant in locally advanced rectal cancer: results of the EXPERT-C trial	wrong outcomes not reported survival and	round II

		recurrence data for primary outcome	
rayyan-661154443	KRAS-IRF2 Axis Drives Immune Suppression and Immune Therapy Resistance in Colorectal Cancer	wrong article type including book chapter, review or molecoular phase I and II study	round II
rayyan-661154445	Progression inference for somatic mutations in cancer	wrong article type including book chapter, review or molecoular phase I and II study	round II
rayyan-661154447	RNA biomarkers in colorectal cancer	wrong article type including book chapter, review or molecoular phase I and II study	round II
rayyan-661154458	Villin Expression Is Frequently Lost in Poorly Differentiated Colon Cancer	wrong outcomes not reported survival and recurrence data for primary outcome	round II
rayyan-661154476	Location of colon cancer (right-sided versus left-sided) as a prognostic factor and a predictor of benefit from cetuximab in NCIC CO.17	wrong population including stage IV, I or other neoplasia diagnosis	round II
rayyan-661154498	Genetic Alterations in Locally Advanced Stage II/III Colon Cancer: A Search for Prognostic Markers	wrong article type including book chapter, review or molecoular phase I and II study	round II
rayyan-661154505	Association of Clinicopathologic and Molecular Markers on Stage-specific Survival of Right Versus Left Colon Cancer	wrong population including stage IV, I or other neoplasia diagnosis	round II
rayyan-661154516	SCOT: Tumor Sidedness and the Influence of Adjuvant Chemotherapy Duration on Disease Free Survival (DFS)	wrong outcomes not	round II

		reported survival and recurrence data for primary outcome	
rayyan-661154519	Distal and proximal colon cancers differ in terms of molecular, pathological, and clinical features	wrong outcomes not reported survival and recurrence data for primary outcome	round II
rayyan-661154556	EGFR and KRAS in Colorectal Cancer	wrong article type including book chapter, review or molecoular phase I and II study	round II
rayyan-661154582	Feasibility of next-generation sequencing in clinical practice: results of a pilot study in the Department of Precision Medicine at the University of Campania â€˜Luigi Vanvitelliâ€™	wrong outcomes not reported survival and recurrence data for primary outcome	round II
rayyan-661154585	Predictive and prognostic analysis of PIK3CA mutation in stage III colon cancer intergroup trial	wrong outcomes not reported survival and recurrence data for primary outcome	round II
rayyan-661154591	Effects of mFOLFOX6 regimen combined with carrelizumab on immune function and prognosis in patients with microsatellite instability colorectal cancer	wrong outcomes not reported survival and recurrence data for primary outcome	round II
rayyan-661154593	Risk assessment in Stage II colorectal cancer	wrong outcomes not reported survival and recurrence data for primary outcome	round II
rayyan-661154595	Prognostic and predictive relevance of microsatellite instability in colorectal cancer	wrong outcomes not reported survival and recurrence data	round II

		for primary outcome	
rayyan-661154596	Evaluating immunotherapy in nonmetastatic colorectal cancer	study in recruiting stage with not available results	round II
rayyan-661154600	Pembrolizumab therapy for microsatellite instability high (MSI-H) colorectal cancer (CRC) and non-CRC	wrong population including stage IV, I or other neoplasia diagnosis	round II
rayyan-661154626	A prospective blinded study of microsatellite instability (MSI) as a marker of overall survival (OS) in the adjuvant treatment of colorectal cancer (CRC) patients	study not retrival	round II
rayyan-661154648	Tumor Sidedness, Recurrence, and Survival After Curative Resection of Localized Colon Cancer	wrong outcomes not reported survival and recurrence data for primary outcome	round II
rayyan-661154688	Impact of RAS mutations on immunologic characteristics of the tumor microenvironment (TME) in patients with microsatellite instability-high (MSI-H) or mismatch-repair-deficient (dMMR) colorectal cancer (CRC)	study not retrival	round II
rayyan-661154726	Prediction of relapse-free survival according to adjuvant chemotherapy and regulator of chromosome condensation 2 (RCC2) expression in colorectal cancer	wrong outcomes not reported survival and recurrence data for primary outcome	round II
rayyan-661154748	Randomized phase III clinical trial comparing the combination of capecitabine and oxaliplatin (CAPOX) with the combination of 5-fluorouracil, leucovorin and oxaliplatin (modified FOLFOX6) as adjuvant therapy in patients with operated high-risk stage II or stage III colorectal cancer	wrong outcomes not reported survival and recurrence data for primary outcome	round II
rayyan-661154750	Efficacy of adjuvant chemotherapy in colon cancer with microsatellite instability: a large multicenter AGEO study	wrong outcomes not reported survival and recurrence data for primary outcome	round II
rayyan-661154761	Association of family history with cancer recurrence and survival among patients with stage III colon cancer	wrong outcomes not reported survival and recurrence data	round II

		for primary outcome	
rayyan-661154763	Postdiagnosis Marine Omega-3 Fatty Acid Intake and Survival of Stage III Colon Cancer in North Center Cancer Treatment Group (NCCTG) Phase III Trial N0147 (Alliance)	wrong outcomes not reported survival and recurrence data for primary outcome	round II
rayyan-661154778	Perioperative/Adjuvant atezolizumab with or without the immunomodulatory IMM-101 in patients with MSI-high or MMR-deficient stage III colorectal cancer ineligible for oxaliplatin-based chemotherapyâ€" a randomized Phase II study	study not retrival	round II
rayyan-661154779	Microsatellite instability and loss of heterozygosity at chromosomal location 18q: prospective evaluation of biomarkers for stages II and III colon cancer--a study of CALGB 9581 and 89803	wrong outcomes not reported survival and recurrence data for primary outcome	round II
rayyan-661154835	Prognosis of microsatellite instability and/or mismatch repair deficiency stage III colon cancer patients after disease recurrence following adjuvant treatment: results of an ACCENT pooled analysis of seven studies	wrong outcomes not reported survival and recurrence data for primary outcome	round II
rayyan-661154837	Randomized trial of FOLFOX alone or combined with atezolizumab as adjuvant therapy for patients with stage III colon cancer and deficient DNA mismatch repair or microsatellite instability (ATOMIC, Alliance A021502)	study in recruiting stage with not available results	round II
rayyan-661154838	p27Kip1 in stage III colon cancer: implications for outcome following adjuvant chemotherapy in cancer and leukemia group B protocol 89803	wrong outcomes not reported survival and recurrence data for primary outcome	round II
rayyan-661154845	KRAS mutation in stage III colon cancer and clinical outcome following intergroup trial CALGB 89803	wrong outcomes not reported survival and recurrence data for primary outcome	round II
rayyan-661154846	Predictive and prognostic roles of BRAF mutation in stage III colon cancer: results from intergroup trial CALGB 89803	subsequent publication with the same patients	round II
rayyan-661154847	Prognostic role of KRAS and BRAF in stage II and III resected colon cancer: results of the translational study on the PETACC-3, EORTC 40993, SAKK 60-00 trial	wrong outcomes not reported survival and	round II

		recurrence data for primary outcome	
rayyan-661155017	A phase II trial to find the durable clinical benefit of nivolumab in class II expressing microsatellite colorectal cancer	wrong outcomes not reported survival and recurrence data for primary outcome	round II
rayyan-661155043	Prognostic value of disease-free interval in colorectal cancer: Is it time?	wrong population including stage IV, I or other neoplasia diagnosis	round II
rayyan-661155146	Follow-up to the MOSAIC study (multicentre international study of oxaliplatin/5-fluorouracil/leucovorin in the adjuvant treatment of colon cancer)	wrong outcomes not reported survival and recurrence data for primary outcome	round II
rayyan-661155185	SCOT - Short Course Oncology Therapy: a study of adjuvant chemotherapy in colorectal cancer	wrong outcomes not reported survival and recurrence data for primary outcome	round II
rayyan-661155278	Novel molecular classification of colorectal cancer and correlation with survival	wrong population including stage IV, I or other neoplasia diagnosis	round II
rayyan-661155306	MTOR/4EBP1 signaling and MMR status in colorectal cancer: New correlations and arising perspectives	wrong population including stage IV, I or other neoplasia diagnosis	round II
rayyan-661155329	Prognostic Impact of Primary Tumor Sidedness in Stage III Colorectal Cancer: Real-World Evidence from a Brazilian Cohort	wrong outcomes not reported survival and recurrence data for primary outcome	round II
rayyan-661155348	Prevalence of recurrent oncogenic fusion in mismatch repair-deficient colorectal carcinoma with hypermethylated MLH1 and wild-type BRAF and KRAS	wrong population including stage	round II

		IV, I or other neoplasia diagnosis	
rayyan-661155357	The Impact of KRAS Mutation on the Presentation and Prognosis of Non-Metastatic Colon Cancer: an Analysis from the National Cancer Database	wrong outcomes not reported survival and recurrence data for primary outcome	round II
rayyan-661155393	Efficacy of PD-1 inhibitors for colorectal cancer and polyps in Lynch syndrome patients	wrong population including stage IV, I or other neoplasia diagnosis	round II
rayyan-661155438	The inhibitory receptor CD94/NKG2A on CD8+ tumor-infiltrating lymphocytes in colorectal cancer: a promising new druggable immune checkpoint in the context of HLAE/Î²2m overexpression	wrong outcomes not reported survival and recurrence data for primary outcome	round II
rayyan-661155461	Prognosis and molecular characteristics of IBD-associated colorectal cancer: Experience from a French tertiary-care center	wrong population including stage IV, I or other neoplasia diagnosis	round II
rayyan-661155515	A comparison of performance of 6-mononucleotide site panel and NCI panel for microsatellite instability detection in patients with colorectal adenocarcinoma	wrong outcomes not reported survival and recurrence data for primary outcome	round II
rayyan-661155521	Distinct Molecular Profiles of Sporadic Early-Onset Colorectal Cancer: A Population-Based Cohort and Systematic Review	wrong outcomes not reported survival and recurrence data for primary outcome	round II
rayyan-661155563	Clinical Utility of KRAS and BRAF Mutations in a Cohort of Patients With Colorectal Neoplasms Submitted for Microsatellite Instability Testing	wrong outcomes not reported survival and recurrence data for primary outcome	round II
rayyan-661155830	Microsatellite instability and BRAF mutation testing in colorectal cancer prognostication.	wrong outcomes not	round II

		reported survival and recurrence data for primary outcome	
rayyan-661155837	The prognostic role of microsatellite instability in colorectal cancer patients.	wrong outcomes not reported survival and recurrence data for primary outcome	round II
rayyan-661155838	Same-Cell Co-Occurrence of RAS Hotspot and BRAF V600E Mutations in Treatment-Naive Colorectal Cancer.	wrong outcomes not reported survival and recurrence data for primary outcome	round II
rayyan-661155846	Combination of microsatellite instability and BRAF mutation status for subtyping colorectal cancer.	wrong population including stage IV, I or other neoplasia diagnosis	round II
rayyan-661155847	The prognostic significance of KRAS and BRAF mutation status in Korean colorectal cancer patients.	wrong population including stage IV, I or other neoplasia diagnosis	round II
rayyan-661155849	The impact of KRAS mutation, microsatellite instability, and tumor laterality on the prognosis of nonmetastatic colon cancer.	wrong population including stage IV, I or other neoplasia diagnosis	round II
rayyan-661155854	Colorectal cancer molecular classification using BRAF, KRAS, microsatellite instability and CIMP status: Prognostic implications and response to chemotherapy.	wrong population including stage IV, I or other neoplasia diagnosis	round II
rayyan-661155859	CMS-dependent prognostic impact of KRAS and BRAFV600E mutations in primary colorectal cancer.	wrong population including stage IV, I or other neoplasia diagnosis	round II
rayyan-661155860	Association of KRAS mutation with tumor deposit status and overall survival of colorectal cancer.	wrong population including stage	round II

		IV, I or other neoplasia diagnosis	
rayyan-661155861	Prognostic Value of BRAFÂ andÂ KRASÂ Mutations in MSI and MSS Stage III Colon Cancer.	wrong outcomes not reported survival and recurrence data for primary outcome	round II
rayyan-661155864	The prognostic role of microsatellite instability, codon-specific KRAS, and BRAF mutations in colon cancer.	wrong population including stage IV, I or other neoplasia diagnosis	round II
rayyan-661155868	BRAF mutation may have different prognostic implications in early- and late-stage colorectal cancer.	wrong population including stage IV, I or other neoplasia diagnosis	round II
rayyan-661155873	Microsatellite instability and mutations in BRAF and KRAS are significant predictors of disseminated disease in colon cancer.	wrong population including stage IV, I or other neoplasia diagnosis	round II
rayyan-661155875	Prognostic Value of BRAF and KRAS Mutation in Relation to Colorectal Cancer Survival in Iranian Patients: Correlated to Microsatellite Instability.	wrong population including stage IV, I or other neoplasia diagnosis	round II
rayyan-661155876	Fusion Kinases Identified by Genomic Analyses of Sporadic Microsatellite Instability-High Colorectal Cancers.	wrong outcomes not reported survival and recurrence data for primary outcome	round II
rayyan-661155878	Prognostic significance of BRAF mutation alone and in combination with microsatellite instability in stage III colon cancer.	wrong outcomes not reported survival and recurrence data for primary outcome	round II
rayyan-661155883	Clinicopathological and protein characterization of BRAF- and K-RAS-mutated colorectal cancer and implications for prognosis.	wrong population including stage IV, I or other	round II

		neoplasia diagnosis	
rayyan-661155892	PD-L1 Expression in colorectal carcinoma and its correlation with clinicopathological parameters, microsatellite instability and BRAF mutation.	wrong population including stage IV, I or other neoplasia diagnosis	round II
rayyan-661155894	BRAF mutation status and survival after colorectal cancer diagnosis according to patient and tumor characteristics.	wrong outcomes not reported survival and recurrence data for primary outcome	round II
rayyan-661155899	Combined microsatellite instability and BRAF gene status as biomarkers for adjuvant chemotherapy in stage III colorectal cancer.	wrong outcomes not reported survival and recurrence data for primary outcome	round II
rayyan-661155900	Mutation Status and Prognostic Value of KRAS and BRAF in Southeast Iranian Colorectal Cancer Patients: First Report from Southeast of Iran.	wrong population including stage IV, I or other neoplasia diagnosis	round II
rayyan-661155901	TGFBR2 and BAX mononucleotide tract mutations, microsatellite instability, and prognosis in 1072 colorectal cancers.	wrong population including stage IV, I or other neoplasia diagnosis	round II
rayyan-661155921	Associations of defect mismatch repair genes with prognosis and heredity in sporadic colorectal cancer.	wrong population including stage IV, I or other neoplasia diagnosis	round II
rayyan-661155926	BRAF mutations in colorectal cancer are associated with distinct clinical characteristics and worse prognosis.	wrong population including stage IV, I or other neoplasia diagnosis	round II
rayyan-661155928	Alternative splicing expands the prognostic impact of KRAS in microsatellite stable primary colorectal cancer.	wrong population including stage IV, I or other neoplasia diagnosis	round II

rayyan-661155936	KRAS mutation and microsatellite instability: two genetic markers of early tumor development that influence the prognosis of colorectal cancer.	wrong population including stage IV, I or other neoplasia diagnosis	round II
rayyan-661155941	The interaction between BRAF mutation and microsatellite instability (MSI) status in determining survival outcomes after adjuvant 5FU based chemotherapy in stage III colon cancer.	wrong outcomes not reported survival and recurrence data for primary outcome	round II
rayyan-661155959	Genetic and epigenetic classifications define clinical phenotypes and determine patient outcomes in colorectal cancer.	wrong population including stage IV, I or other neoplasia diagnosis	round II
rayyan-661155965	Characterization of Chilean patients with sporadic colorectal cancer according to the three main carcinogenic pathways: Microsatellite instability, CpG island methylator phenotype and Chromosomal instability.	wrong outcomes not reported survival and recurrence data for primary outcome	round II
rayyan-661155966	Combined analysis of specific KRAS mutation, BRAF and microsatellite instability identifies prognostic subgroups of sporadic and hereditary colorectal cancer.	wrong outcomes not reported survival and recurrence data for primary outcome	round II
rayyan-661155974	PD-L1 expression in colorectal cancer is associated with microsatellite instability, BRAF mutation, medullary morphology and cytotoxic tumor-infiltrating lymphocytes.	wrong outcomes not reported survival and recurrence data for primary outcome	round II
rayyan-661155976	Mutational Analysis of Patients With Colorectal Cancer in CALGB/SWOG 80405 Identifies New Roles of Microsatellite Instability and Tumor Mutational Burden for Patient Outcome.	wrong population including stage IV, I or other neoplasia diagnosis	round II
rayyan-661155980	Molecular classification of colorectal cancer using the gene expression profile of tumor samples.	wrong outcomes not reported survival and recurrence data	round II

		for primary outcome	
rayyan-661155981	Evaluating the Combination ofÂ Microsatellite Instability andÂ Mutation in BRAF as PrognosticÂ Factors for Patients WithÂ Colorectal Cancer.	wrong article type including book chapter, review or molecoular phase I and II study	round II
rayyan-661155988	Clinicopathological characteristics and prognostic impact of colorectal cancers with NRAS mutations.	wrong population including stage IV, I or other neoplasia diagnosis	round II
rayyan-661155989	Prognostic significance of alterations in KRAS isoforms KRAS-4A/4B and KRAS mutations in colorectal carcinoma.	wrong population including stage IV, I or other neoplasia diagnosis	round II
rayyan-661155995	Prognostic, predictive, and pharmacogenomic assessments of CDX2 refine stratification of colorectal cancer.	wrong population including stage IV, I or other neoplasia diagnosis	round II
rayyan-661156008	The long-term survival characteristics of a cohort of colorectal cancer patients and baseline variables associated with survival outcomes with or without time-varying effects.	wrong population including stage IV, I or other neoplasia diagnosis	round II
rayyan-661156027	f	wrong outcomes not reported survival and recurrence data for primary outcome	round II
rayyan-661156029	Association between molecular subtypes of colorectal cancer and patient survival.	wrong population including stage IV, I or other neoplasia diagnosis	round II
rayyan-661156044	Combined methylation of p16 and hMLH1 (CMETH2) discriminates a subpopulation with better prognosis in colorectal cancer patients with microsatellite instability tumors.	wrong population including stage IV, I or other neoplasia diagnosis	round II

rayyan-661156045	Association Between Molecular Subtypes of Colorectal Tumors and Patient Survival, Based on Pooled Analysis of 7 International Studies.	wrong population including stage IV, I or other neoplasia diagnosis	round II
rayyan-661156071	High-frequency microsatellite instability and BRAF mutation (V600E) in unselected Serbian patients with colorectal cancer.	wrong population including stage IV, I or other neoplasia diagnosis	round II
rayyan-661156081	Associations between colorectal cancer molecular markers and pathways with clinicopathologic features in older women.	wrong population including stage IV, I or other neoplasia diagnosis	round II
rayyan-661156086	Prognostic significance of AMP-activated protein kinase expression and modifying effect of MAPK3/1 in colorectal cancer.	wrong population including stage IV, I or other neoplasia diagnosis	round II
rayyan-661156091	Coexistence of MSI with KRAS mutation is associated with worse prognosis in colorectal cancer.	wrong population including stage IV, I or other neoplasia diagnosis	round II
rayyan-661156115	Molecular analysis of colorectal tumors within a diverse patient cohort at a single institution.	wrong population including stage IV, I or other neoplasia diagnosis	round II
rayyan-661156117	DNA methylation predicts recurrence from resected stage III proximal colon cancer.	wrong population including stage IV, I or other neoplasia diagnosis	round II
rayyan-661156119	Marine omega-3 fatty acid intake and survival of stage III colon cancer according to tumor molecular markers in NCCTG Phase III trial N0147 (Alliance).	wrong population including stage IV, I or other neoplasia diagnosis	round II
rayyan-661156134	Use of multivariate analysis to suggest a new molecular classification of colorectal cancer.	wrong population including stage IV, I or other	round II

		neoplasia diagnosis	
rayyan-661156136	Influence of Molecular Status on Recurrence Site in Patients Treated for a Stage III Colon Cancer: a Post Hoc Analysis of the PETACC-8 Trial.	wrong outcomes not reported survival and recurrence data for primary outcome	round II
rayyan-661156138	Cancer risk and overall survival in mismatch repair proficient hereditary non-polyposis colorectal cancer, Lynch syndrome and sporadic colorectal cancer.	wrong outcomes not reported survival and recurrence data for primary outcome	round II
rayyan-661156154	Promoter methylation of Wnt5a is associated with microsatellite instability and BRAF V600E mutation in two large populations of colorectal cancer patients.	wrong outcomes not reported survival and recurrence data for primary outcome	round II
rayyan-661156160	Integrated analysis of molecular and clinical prognostic factors in stage II/III colon cancer.	wrong outcomes not reported survival and recurrence data for primary outcome	round II
rayyan-661156168	High MACC1 expression in combination with mutated KRAS G13 indicates poor survival of colorectal cancer patients.	wrong outcomes not reported survival and recurrence data for primary outcome	round II
rayyan-661156170	A prognostic CpG score derived from epigenome-wide profiling of tumor tissue was independently associated with colorectal cancer survival.	wrong outcomes not reported survival and recurrence data for primary outcome	round II
rayyan-661156174	Clinicopathologic characteristics, CpG island methylator phenotype, and BRAF mutations in microsatellite-stable colorectal cancers without chromosomal instability.	wrong population including stage IV, I or other neoplasia diagnosis	round II
rayyan-661156184	Prognostic implication of the CpG island methylator phenotype in colorectal cancers depends on tumour location.	wrong population	round II

		including stage IV, I or other neoplasia diagnosis	
rayyan-661156185	Deficient DNA mismatch repair is associated with favorable prognosis in Thai patients with sporadic colorectal cancer.	wrong population including stage IV, I or other neoplasia diagnosis	round II
rayyan-661156191	DNA mismatch repair status and colon cancer recurrence and survival in clinical trials of 5-fluorouracil-based adjuvant therapy.	wrong outcomes not reported survival and recurrence data for primary outcome	round II
rayyan-661156192	Clinical meaning of BRAF mutation in Korean patients with advanced colorectal cancer.	wrong population including stage IV, I or other neoplasia diagnosis	round II
rayyan-661156201	Mutations in the RAS-MAPK, PI(3)K (phosphatidylinositol-3-OH kinase) signaling network correlate with poor survival in a population-based series of colon cancers.	wrong outcomes not reported survival and recurrence data for primary outcome	round II
rayyan-661156208	The BRAF V600E mutation is an independent prognostic factor for survival in stage II and stage III colon cancer patients.	wrong outcomes not reported survival and recurrence data for primary outcome	round II
rayyan-661156223	Is KRAS mutation associated with interval colorectal cancers?	wrong population including stage IV, I or other neoplasia diagnosis	round II
rayyan-661156225	Prognostic implications of CpG island hypermethylator phenotype in colorectal cancers.	wrong population including stage IV, I or other neoplasia diagnosis	round II
rayyan-661156254	Clinicopathological and biomolecular characteristics of stage IIB/IIC and stage IIIA colon cancer: Insight into the survival paradox.	wrong outcomes not reported	round II

		survival and recurrence data for primary outcome	
rayyan-661156261	Hypermethylator phenotype in sporadic colon cancer: study on a population-based series of 582 cases.	wrong outcomes not reported survival and recurrence data for primary outcome	round II
rayyan-661156277	BRAFV600E immunohistochemistry in conjunction with mismatch repair status predicts survival in patients with colorectal cancer.	wrong population including stage IV, I or other neoplasia diagnosis	round II
rayyan-661156285	ASO Visual Abstract: Conditional Survival After Diagnosis of Non-metastatic Colon Cancer: Impact of Laterality, MSI, and KRAS Status.	study not retrival	round II

Table S2. Newcastle-Ottawa scale of selected studies[a]

**Reference**	**Selection domain**				**Comparability domain**	Outcome domain			**Quality score**
	Representativeness of the exposed cohort	Non-exposed cohort drawn from the same community	Secure ascertainment of exposure	Outcome not present at start	Adjustment for confounders in multivariate analysis	Adequate assessment of outcome	Adequate length of follow-up	Completeness of the follow up	
**Hutchins G, 2011**	*	*	*	*	No	*	*	*	8
**Taieb J, 2023**	*	*	*	*	*	*	*	*	8
**Domingo E, 2018**	*	*	*	*	*	*	*	*	8
**Maestro M L, 2006**	*	*	*	*	No	*	*	No	6
**Batur S, 2016**	*	*	No	*	No	*	*	No	5
**De Cuba E M V, 2016**	*	*	*	*	No	*	*	No	6
**Kadowaki S, 2015**	*	*	No	*	No	No	*	*	5

**Nakaji Y, 2017**	*	*	No	*	No	*	*	No	5

[a] Good quality: > 7 stars; Fair quality: 5-7 stars; Poor quality: < 5 sta
